# Analgesic drug efficacy in mouse postoperative pain: a systematic review and meta-analysis

**DOI:** 10.1038/s41684-026-01750-5

**Published:** 2026-06-23

**Authors:** Roberta Giusti Schran, Patrícia Rodrigues, Gabriela Trevisan, Juliano Ferreira

**Affiliations:** 1 Graduate Program in Pharmacology, Federal University of Santa Catarina, Florianópolis, Brazil; 2 Graduate Program in Pharmacology, Federal University of Santa Maria, Santa Maria, Brazil

**Keywords:** Pharmacology, Animal behaviour

## Abstract

The management of postoperative pain is a critical ethical concern and a challenge in laboratory animal research, particularly for mice. Surgical procedures are routinely conducted in mice, but the use of analgesic drugs is underreported and the assessment of their efficacy is limited. This systematic review assesses the efficacy of analgesic drugs for postoperative pain in mice, addressing the influence of various factors, including sex, surgical invasiveness, pain modalities and analgesic classes. A systematic literature search resulted in 48 eligible studies included in the qualitative analysis and 43 in the quantitative analysis. The overall pooled standardized mean difference (SMD) for analgesic drugs was 0.46 (95% confidence interval 0.31 to 0.60), indicating a positive pain-reducing effect. Subgroup analysis revealed that analgesic treatment was significantly more effective in male (SMD 0.84; 0.60 to 1.08) than in female mice, in mild (SMD 0.57; 0.38 to 0.75) than in severe surgical procedures and in reducing evoked pain (SMD 1.12; 0.83 to 1.41) than in reducing spontaneous pain. In addition, almost all analgesic drug classes tested, including opioids, nonsteroidal anti-inflammatory drugs, acetaminophen and local anesthetics were effective in reducing evoked pain but not spontaneous pain (SMD 0.12; −0.03 to 0.27). However, most studies present limitations that could produce a high risk of bias. Taken together, our results indicate that, although analgesic drugs can reduce postoperative pain in mice, their efficacy is reduced in females, severely invasive surgeries and spontaneous pain. Thus, high-quality studies are still needed to answer ethical concerns and to guarantee full analgesia in laboratory mice submitted to surgical procedures.

## Abstract (web summary)

This study systematically reviews analgesic efficacy for postoperative pain in mice, showing overall pain reduction but reduced effects in females, severe surgeries and spontaneous pain, stressing the need for higher-quality studies.

## Main

The management of postoperative pain (POP) poses a clinical challenge not only in humans but also in laboratory animals^1,2^. Laboratory mice, the most used species in research^3,4^, undergo intentional surgical procedures, such as skin and muscle incision, laparotomy and thoracotomy, to induce diseases (that is, traumatic neuropathy) and implant medical devices (that is, cannulas and transmitters)^5^. However, in a review that evaluated the animal survival rate after surgeries, most of the studies did not even mention the use of analgesic drugs^5^. Of note, untreated or undertreated pain can cause distress and affect research results^6^.

In this context, POP occurrence in laboratory animals is a substantial concern in biomedical research, as animal welfare is an important ethical consideration^7,8^. Analgesic drug classes are outlined for managing POP in mice, and regulations require the use of analgesics after surgeries (unless their use would confound the outcome of the study)^9^. However, the overall analgesic efficacy remains unconfirmed^10,11^. The lack of analgesic treatment facilitates the appearance of oligoanalgesia, that is, undertreatment of acute pain^12^. In fact, a systematic scoping review of analgesia in rodents suggested that inadequate analgesia and oligoanalgesia are persistent issues associated with experimental intracranial surgery^13^. In addition, several factors may influence the analgesic efficacy in POP, including the animal’s sex, type of surgery, surgical invasiveness and pain modalities assessed (evoked versus spontaneous)^14,15^. Therefore, there is a lack of guidelines and protocols for the pharmacological treatment of POP in mice^5^.

There are few guidelines that indicate what analgesic drugs must be used to treat POP in mice^16–20^. These guidelines have many limitations, including the fact that they are not specific to POP and are based on low levels of clinical evidence (usually expert opinions not backed by studies)^16^. Therefore, there is a need for improved practices for POP management in mice based on more robust evidence of analgesic effectiveness, ensuring ethical limits are met, enhancing the refinement of experimental models and improving translational outcomes^21,22^.

To answer the research question ‘Which analgesic drugs are effective in the treatment of mouse POP?’, we systematically analyzed the literature considering factors such as sex, surgical invasiveness and pain modality. Our goal was to provide robust evidence on analgesic efficacy in mouse POP, thereby refining experimental models, enhancing animal welfare and improving the quality of research outcomes. We hypothesize that a systematic review and meta-analysis focusing on the effectiveness of analgesics in mice will provide evidence of the current need for improvement of POP management practices.

## Results

### Study selection

**Fig. 1:**
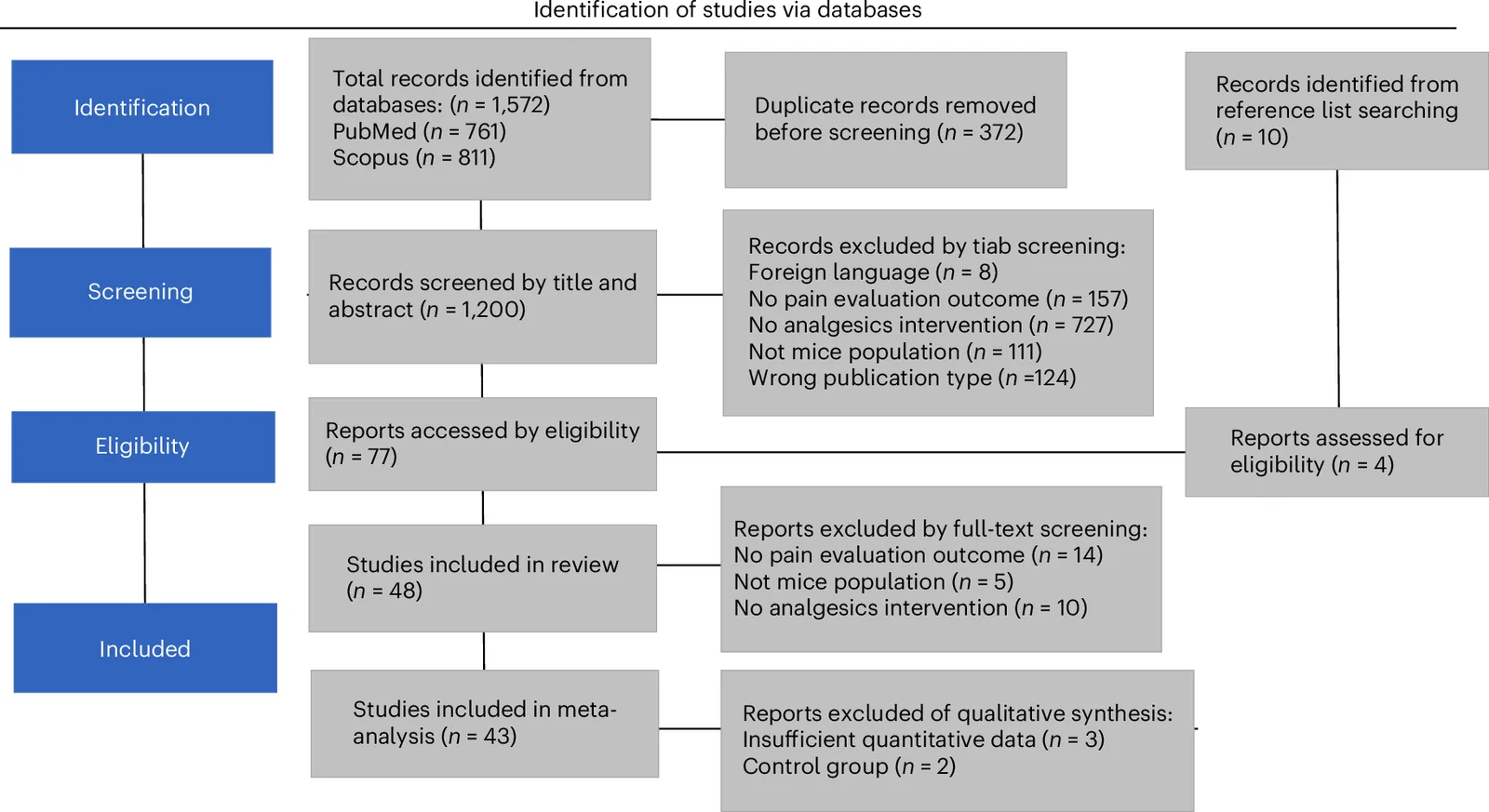
PRISMA flow diagram illustrating the article search and screening process. The diagram summarizes the identification, screening, eligibility assessment and inclusion of studies. A total of 1,572 records were initially retrieved, 1,200 remained after removing duplicates, and 1,127 were excluded after abstract screening. Seventy-three full texts were assessed, and 4 additional studies were identified via manual searching, resulting in 48 studies included in the qualitative synthesis and 43 included in the meta-analysis. Source data

The flow diagram in Fig. 1 illustrates the study selection process. An initial database search identified 1,572 citations. After duplicate entries were removed, 1,200 citations remained. Subsequently, 1,127 of these citations were excluded based on a review of their abstracts, as they did not meet the criteria for inclusion outlined in Table 1. Thus, 73 studies were retrieved. Through manual searching, we identified ten potential studies from reference list searching, six of which were excluded with valid justification, retrieving four more studies. A more comprehensive examination was conducted of the full texts of the remaining 77 citations. Finally, 48 studies were included in the qualitative synthesis, but 5 studies were excluded from the meta-analysis as those were missing a control group (2 studies) and insufficient quantitative data (3 studies). Among these, 43 studies that conducted further experimentation to gain an understanding of POP in mice in conjunction with the detection of any pain modality, that is the specific type or dimension of pain assessed (for example, evoked or spontaneous pain, measured through behavioral tests) were included in the meta-analysis.

**Table 1:** Search strategy syntax representing the utilized search strings in the databases

Clinical question	Search strategy^a^	Inclusion criteria	Exclusion criteria
Operated mice	(Mice [mesh terms], mice [all fields], mouse [all fields], murine [all fields]) AND (Operated [all fields] OR Surgery [all fields] OR Operative [all fields] OR Postoperative [all fields] OR Post-operative [all fields] OR Surgical Procedures, Operative [mesh terms], OR Surgical incision [tiab] OR Pain, Postoperative [mesh terms/tiab] OR Postoperative pain [tiab]	Studies that used mice as subjects Studies involving mice of any sex and age Articles conducting surgical procedures Studies that complied with guidelines or legislation to minimize animal distress	Articles that did not involve surgical procedures Animals that were subjected to pain models not stemming from surgical interventions Studies involving species other than mice Studies that did not consider ethical principles Studies that did not report the sample size
Analgesic drug	Analgesics [mesh terms] OR Analgesics, Non-Narcotic [mesh terms/all fields] OR Anti-inflammatory Agents, Non-Steroidal [mesh terms] OR – all fields OR Analgesics, Opioid [mesh terms] OR Analgesics, Opioid [Pharmacological Action], OR Anesthetics, Local [mesh terms/tiab] OR Anesthetics, Local [Pharmacological Action] OR Anesthetics [mesh terms]	Various routes of administration, including oral, subcutaneous, intraperitoneal, intraneural (regional block), intrathecal and intravenous Articles assessing the effect of treatment timing (pre-, trans- and postoperative) were considered	Articles that did not assess the analgesic-like effects following treatment Studies that did not report the administered dosage Research that did not focus on POP treatment
Analgesic-like effect	Pain [Mesh terms] OR Pain measurement [mesh/all fields] OR Hyperalgesia [mesh terms/all fields] OR Nociception [mesh terms/all fields]	Studies that assessed and documented the effectiveness of analgesic intervention Articles that included a control group (operated without treatment or treated with vehicle or saline)	Studies that did not evaluate or adequately describe pain Articles lacking control groups Studies with insufficient quantitative data

^a^The same strategy was conducted in Scopus with adaptations to the database.

### Characteristics of studies included in the meta-analysis

Studies eligible for both qualitative and quantitative synthesis were categorized according to pain modality, and these are summarized in Table 2. The assessment of evoked pain predominantly relied on the von Frey test (mechanical hyperalgesia, 20/43). Thermal hyperalgesia was evaluated exclusively through the Hargreaves test (heat hyperalgesia, 8/43), and cold hyperalgesia was not reported across all studies. Spontaneous pain was assessed by the mouse grimace scale (MGS, 7/43), by clinical and/or behavioral investigation (including ethogram scores) (12/43), as well as by the assessment of guarding behavior (protective postures or movements that animals exhibit to minimize pain or discomfort, particularly around the operated site) (4/43). A smaller subset of studies also explored behaviors suppressed by pain, such as burrowing (4/43), locomotor activity (3/43) and nest building (5/43). Individual studies could involve more than one drug treatment, pain evaluation outcome, drug administration route or frequency of administration.

**Table 2:** Categorization of studies included in the systematic review based on modalities, evaluated behaviors and outcome measures for pain assessment

Pain modality	Test/behavior evaluated	Outcome measure	Number of studies	Number of Comparisons	References
Evoked pain	von Frey	Paw withdrawal threshold	20	154	^30,56–76^
	Hargreaves	Withdrawal latency	8	67	^57,59,63,66,68,69,75,77,78^
Spontaneous pain	Grimace	Scores	7	185	^10,22,32,44,79–81^
	Guarding	Scores	4	13	^56,58,59,72^
	Clinical and behavioral investigation	Scores	12	55	^79,80,82–91^
Behaviors suppressed by pain	Nest building	Nest score	5	10	^21,79,80,84,86^
	Burrowing	Dig latency	4	7	^11,79,85,92^
	Locomotor activity	Open field, running-wheel activity	3	11	^58,82,86^
Total number of comparisons^a^				502	

^a^Number of comparisons by pain modality.

The studies and comparisons covered by this study are detailed in Table 3. Most studies were conducted in male mice (25 studies), 14 studies were conducted in female mice and only 4 studies investigated both sexes. Regarding mouse strains, the most common were C57BL/6 (17/43), CD1 (6/43), ICR (5/43), Swiss (4/43), C3H, BalbC and Kunming (2/43); FVB, C3H and DDY strains were each represented once (1/43). Studies that investigated more than one strain were mainly represented by C57BL/6 and C3H (3/43) and C57BL/6 and DBA (1/43). In terms of administration route, the subcutaneous (SC) route was the most used method (24/43), followed by oral (PO) administration (4/43) and intraperitoneal (IP) administration (5/43). To a lesser extent, locoregional (NB) (1/43) and intrathecal (IT) (1/43) administration methods were also observed. Five studies used SC and PO routes of administration (5/43), and one study used IP and SC routes (1/43).

**Table 3:** Summary of study characteristics and subgroups included in the systematic review

Characteristics	Subgroups	*N* of studies	*N* of comparisons	References
Sex	Male	25	243	^10,21,32,56–59,62–68,70,71,73–76,79,87,88,90,91^
	Female	14	182	^10,44,69,72,77,79,81–86,89,92^
	Both	4	77	^11,22,30,93^
Mouse strain	C57BL/6	17	156	^11,30,44,58,62,63,66,69,74,75,79–81,85,89,92,93^
	CD1	6	190	^10,22,32,57,70,77^
	ICR	5	54	^21,58,76,83,86^
	Swiss	4	35	^56,59,71,72^
	C3H	1	5	^68^
	Kunming	2	12	^73,94^
	BalbC	2	4	^67,88^
	FVB	1	18	^82^
	DDY	1	8	^65^
	C57BL/6-DBA	1	4	^93^
	C57BL/6-C3H	3	16	^87,90,91^
Route	SC	24	67	^11,21,22,32,44,56,58,59,62,63,66,68,70,72,77,82–85,88–92^
	PO	4	74	^56,57,65,80^
	IP	5	18	^64,67,71,74,76^
	NB	1	2	^75^
	IT	1	4	^73^
	SC/PO	5	166	^10,30,79,93^
	SC/IP	1	18	^69^
Surgery type | invasiveness	Plantar incision | mild	17	196	^56–59,62,64–73,75,76^
	Laparotomy | moderate	15	154	^11,21,30,62,77,79,80,83–86,88,89,92,93^
	Vasectomy/laparotomy | moderate	5	16	^79,87,90,91,95^
	Osteotomy | moderate	2	7	^63,81^
	Vasectomy/scrotal | mild	1	4	^32^
	Craniotomy | mild	1	100	^10^
	Mammary fat pad removal | mild	1	18	^82^
	SMIR | moderate	1	3	^74^
	Thoracotomy | severe	1	1	^44^
	Splenectomy | moderate	1	3	^58^

IP, intraperitoneal; IT, intrathecal; NB, nerve block; PO, oral; SC, subcutaneous; SMIR, skin/muscle incision and retraction model.

Among the surgical procedures, the most frequently used was the plantar incision model (mild severity, 17/43), followed by laparotomy (moderate severity, 15/43). Among the ten types of surgical procedures identified, 4/10 were classified as mildly invasive, and 5 were classified as moderate. Only one surgical procedure was classified as severely invasive (thoracotomy). Further details about surgical procedures and invasiveness categorization are provided in Table 3. Most studies compared the analgesic treatment effects of drugs with those of vehicle-treated animals (35/43; Supplementary Table 1).

The main classes of analgesic drugs used to treat POP were opioids (29/43), nonsteroidal anti-inflammatory drugs (NSAIDs) (21/43) and antipyretic/analgesic drugs, represented by acetaminophen (paracetamol, 3/43), used alone. However, metamizole did not meet the criteria for inclusion in the meta-analysis and was not included in the quantitative synthesis (Table 4). Some studies also treated POP with local anesthetics (two studies) and gabapentinoids (two studies), used alone. Among the opioids, buprenorphine (15 studies) and morphine (7 studies) were the most used drugs. Regarding NSAIDs, carprofen (seven studies) and meloxicam (five studies) were the most frequently tested. Only six studies investigated the analgesic potential of multimodal therapy using acetaminophen (three studies) or local anesthetic combinations (three studies). Regarding the frequency of administration, most of the articles reported a single administration (26/43).

**Table 4:** Summary of treatment characteristics and subgroups included in the review

Subgroup/classification	Characteristics/dose range (route)	Frequency (1, 2 or >2)^a^	*N* of studies	*N* of comparisons	References
Opioid	Buprenorphine 0.001–2.2 mg/kg (SC) 1.93 mg/kg (PO)	1	8	56	^10,32,44,66,81,85,86,91^
		2	2	6	^77,82^
		>2	8	57	^10,22,30,58,62,69,85,86^
	Morphine 1–10 mg/kg (SC) 3–10 nmol/site (IT)	1	6	40	^58,59,63,68,72,73^
		2	1	3	^58^
		>2	2	7	^58,73^
	Oxycodone 3 mg/kg (IP)	1	1	1	^64^
	Oxymorphone 2 mg/kg (SC)	>2	1	1	^95^
	Tramadol 25 mg/kg (SC) 21.8–166 mg/kg (PO)	1	3	5	^70,81,92^
		>2	1	3	^89^
NSAID	Carprofen 5–50 mg/kg (SC) 10–25 mg/kg (PO)	1	7	30	^10,11,22,30,82,84,93^
		>2	1	28	^10^
	Celecoxib 1 mg/kg–10 mg/kg (PO)	1	1	8	^65^
	Dexketoprofen 100 mg/kg (SC)	1	1	3	^70^
	Diclofenac 10–30 mg/kg (SC)	>2	1	3	^62^
	Firocoxibe 10 mg/kg–20 mg/kg (IP)	>2	1	12	^69^
	Flunixin meglumine 2.5 mg/kg (SC)	1	1	4	^83^
		>2	1	4	^83^
	Ibuprofen 100–300 mg/kg (IP)	1	1	6	^71^
	Indomethacin 1–10 mg/kg (PO)	1	1	14	^56^
	Ketoprofen 1–50 mg/kg (SC)	1	2	7	^22,63^
	Meloxicam 2–20 mg/kg (SC) 2–5 mg/kg (PO)	1	5	39	^10,32,87,88,90^
		>2	1	20	^10^
Antipyretic/analgesic	Paracetamol 50–400 mg/kg (PO) 100–450 mg/kg (SC) 30–360 mg/kg (IP)	1	2	76	^57,79^
		>2	1	25	^76^
Gabapentinoid	Gabapentin 20–50 mg/kg (IP)	1	1	6	^74^
	Pregabalin Not specified	1	1	1	^67^
Local anesthetic	Bupivacaine 5–10 mg/kg (SC)	1	1	2	^94^
	Ropivacaine 5%/site (NB)	1	1	4	^75^
Multimodal analgesia	Multimodal analgesia	1	3	43	^70,79,80^
		2	2	9	^77,82^
		>2	1	2	^21^

^a^Administration frequency: 1, single ; 2, two; >2, multiple.

### Quality and risk-of-bias assessment of the included studies

**Fig. 2:**
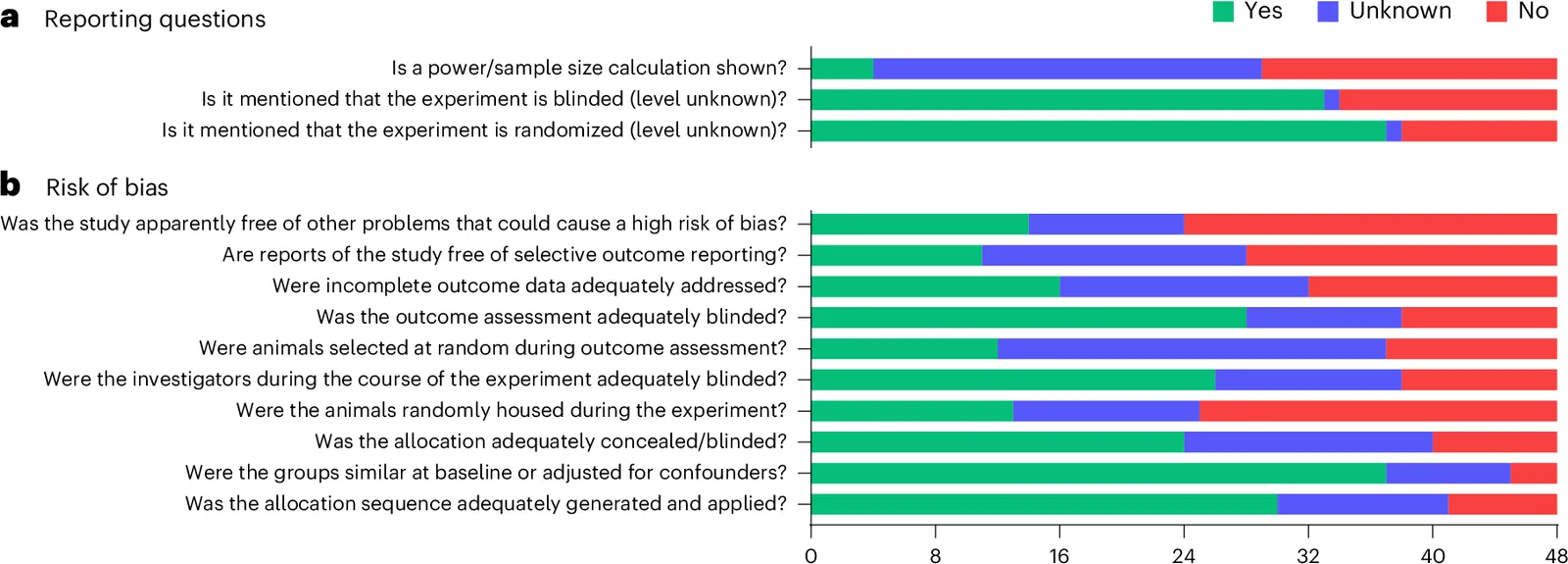
Risk-of-bias assessment of the studies included in the systematic review. **a**, Reporting of methodological practices relevant to internal validity, showing the proportion of studies that explicitly mentioned randomization, blinding at any level, sample size calculation and other reporting components derived from the Systematic Review Center for Laboratory Animal Experimentation (SYRCLE) tool. Each bar represents the percentage of the 48 included studies that reported each item. **b**, Risk-of-bias ratings across all SYRCLE domains, including selection, performance, detection, attrition, reporting and other sources of bias. Each domain is classified as low, unclear or high risk, and the bars represent the distribution of these ratings across the 48 studies. Together, **a** and **b** illustrate the limited reporting and substantial methodological variability among studies included in the review. Source data

Incomplete reporting limited the assessment of study quality and its impact on the magnitude of the standardized mean differences (SMDs) (Fig. 2). Only four of the studies reported sample size calculation (4/48) (Fig. 2a). The majority of the studies mentioned that experiments were blinded (33/48; Fig. 2a) or randomized at any level (37/48; Fig. 2a). Regarding selection bias, approximately half of the studies described sequence generation (30/48), baseline characteristics (37/48) and allocation concealment (24/48) (Fig. 2b). Regarding performance bias, 13 of 48 studies described the conditions used to house animals during the experiments, and 26 of 48 reported that caregivers and/or investigators were blinded to treatment allocation (Fig. 2b). For detection bias, 12 of 48 studies stated that animals were randomly selected for outcome assessment, while only 28 adequately blinded the outcome assessors (Fig. 2b). Attrition bias was appropriately addressed in 16 of 48 studies (Fig. 2b). Reporting bias was low in most studies, with approximately three-quarters judged to be free of selective outcome reporting (11/48), and only 14 of 48 studies appeared to be free from other potential sources of bias (Fig. 2b).

### Meta-analysis of all analgesic treatments in all pain modalities

**Fig. 3:**
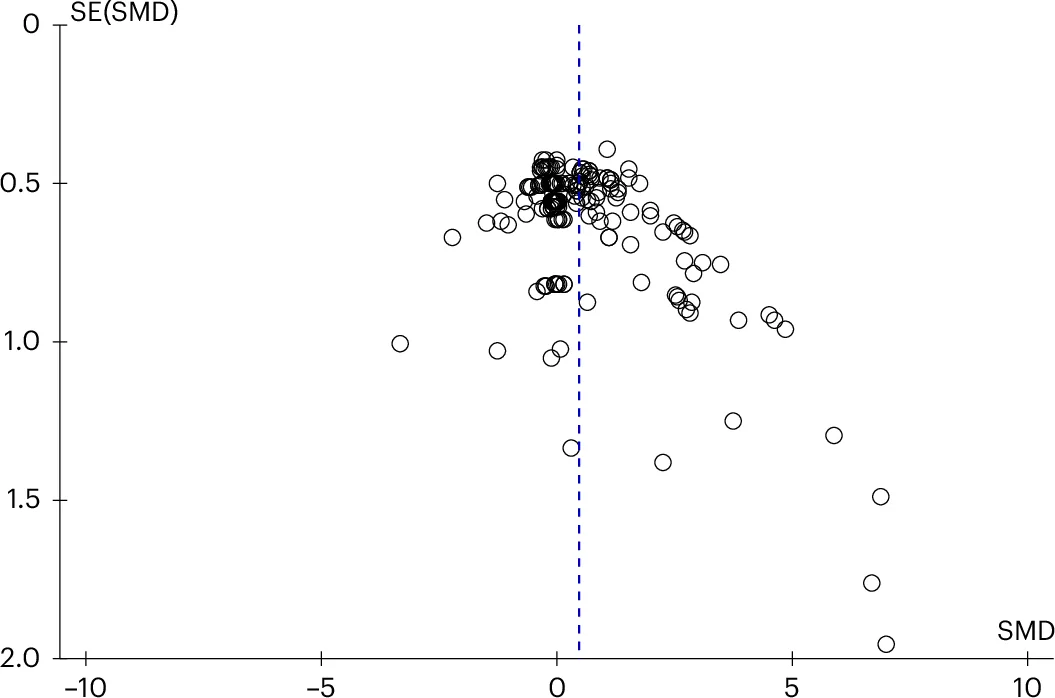
Funnel plot of all studies included in the meta-analysis. The *x* axis presents the SMD, and the *y* axis presents the standard error (SE) of SMD. Funnel plot displaying the relationship between effect size (SMD) and its SE for all comparisons included in the meta-analysis. Each point represents one comparison, allowing visual inspection of potential bias in the aggregated data. Source data

**Fig. 4:**
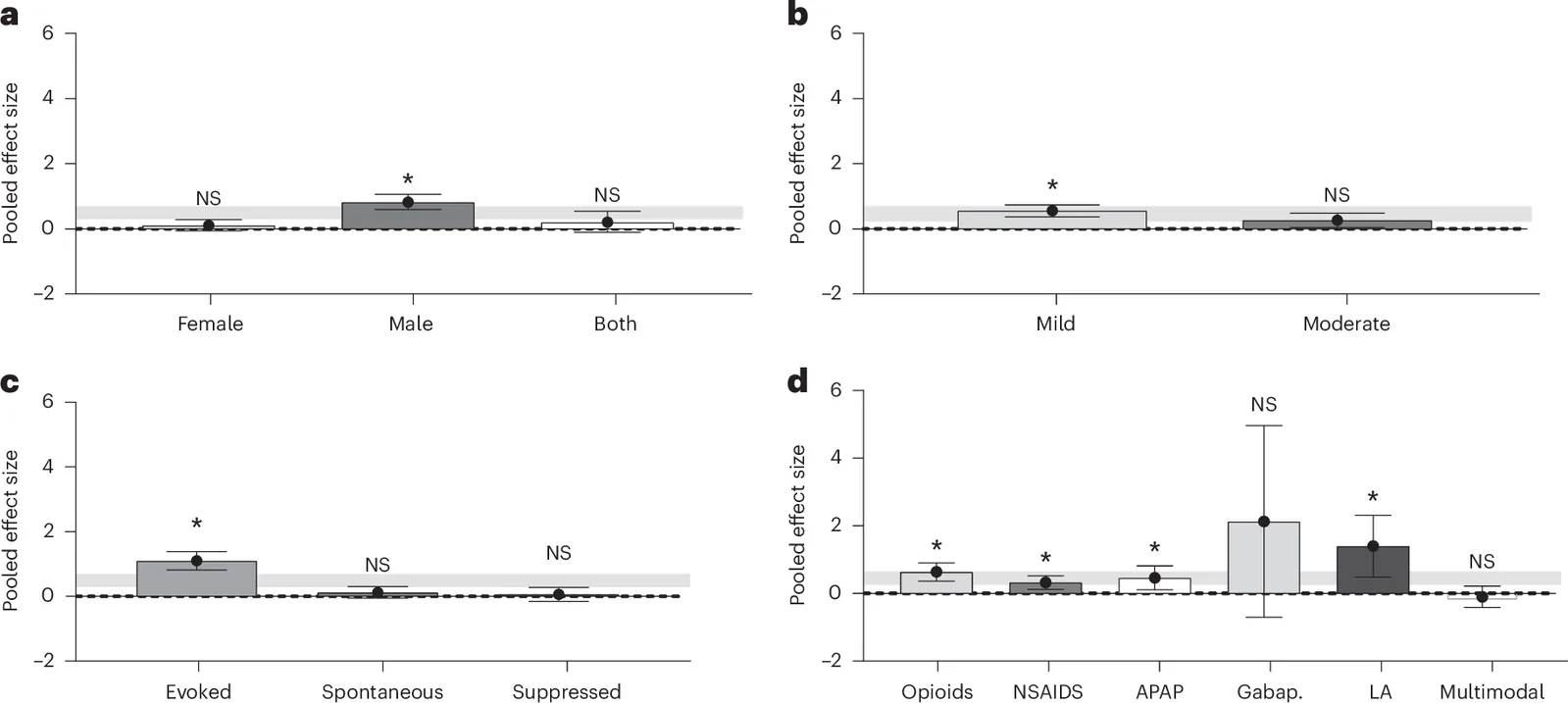
Summary of the pooled effect size of study subgroups included in the meta-analysis. Forest plots illustrating pooled SMDs (95% CI) for subgroup analyses. The gray vertical bar represents the overall effect across all comparisons. **a**, Subgroup analysis by sex: female (*n* = 66), male (*n* = 96) and both sexes (*n* = 10). **b**, Subgroup analysis by surgical severity: mild (*n* = 102) and moderate (*n* = 70). **c**, Subgroup analysis by pain modality: evoked (*n* = 72), spontaneous (*n* = 62) and suppressed behaviors (*n* = 18). **d**, Subgroup analysis by analgesic class: opioids (*n* = 65), NSAIDs (*n* = 55), acetaminophen/paracetamol (APAP, *n* = 21), gabapentinoids (Gabap., *n* = 3), local anesthetics (LA, *n* = 4) and multimodal therapy (*n* = 21). Asterisks indicate statistically significant differences between subgroups (*P* < 0.05, Bonferroni-adjusted test). NS, not significant. Source data

The analgesic treatment effect for all 502 comparisons (pooled SMD) was 0.46 (0.31 to 0.60) (Supplementary Fig. 1). This suggests that the analgesic treatments have a positive pain-reducing effect considering all pain assessment modalities, sex and surgical invasiveness (*Z* = 6.2, *P* < 0.001). Across the 502 comparisons, the funnel plot (Fig. 3) and Egger’s test (*P* < 0.001) showed publication bias due to a few studies reporting negative analgesic treatment effects. The *I*^2^ suggested a important level of heterogeneity, which reflects the diversity of study designs among the 502 comparisons (*χ*^2^ = 562.39, d.f. 185, *P* < 0.001, *I*^2^ = 67%). To investigate the high heterogeneity, we performed a subanalysis when there were at least three comparisons in each subgroup (sex, severity of the surgery, class of analgesic drug and pain modality; Fig. 4).

### Sex

Subgroup analysis based on sex was conducted for all analgesic treatments in all pain modalities (Fig. 4a and Supplementary Fig. 2). The number of comparisons in each category is presented in Table 3. The analysis indicated that the analgesic treatment effect SMD was 0.84 (0.60 to 1.08), 0.11 (−0.06 to 0.29) and 0.09 (−0.14 to 0.32) in studies carried out only with males, only with females, and with both sexes, with high (*I*^2^ = 77%), mild (*I*^2^ = 38%) and no heterogeneity (*I*^2^ = 0%) respectively. The pain treatment was significant only in studies performed in male mice, with differences detected among the subgroups (*Z* = 6.20; *P* < 0.001), indicating that sex was relevant to explaining the overall heterogeneity.

### Surgical severity

The SMDs of the analgesic treatment effect in studies conducted with mild and moderate severity surgeries were 0.57 (0.38 to 0.75) and 0.27 (0.04 to 0.49), respectively (Fig. 4b and Supplementary Fig. 3), with high heterogeneity (*I*^2^ = 69 and 61%). The treatment effect was significant only with mild procedures, and differences were detected among the two subgroups (*Z* = 6.14; *P* = 0.001), indicating that analgesic efficacy is influenced by the invasiveness of the surgical process.

### Pain modalities

**Fig. 5:**
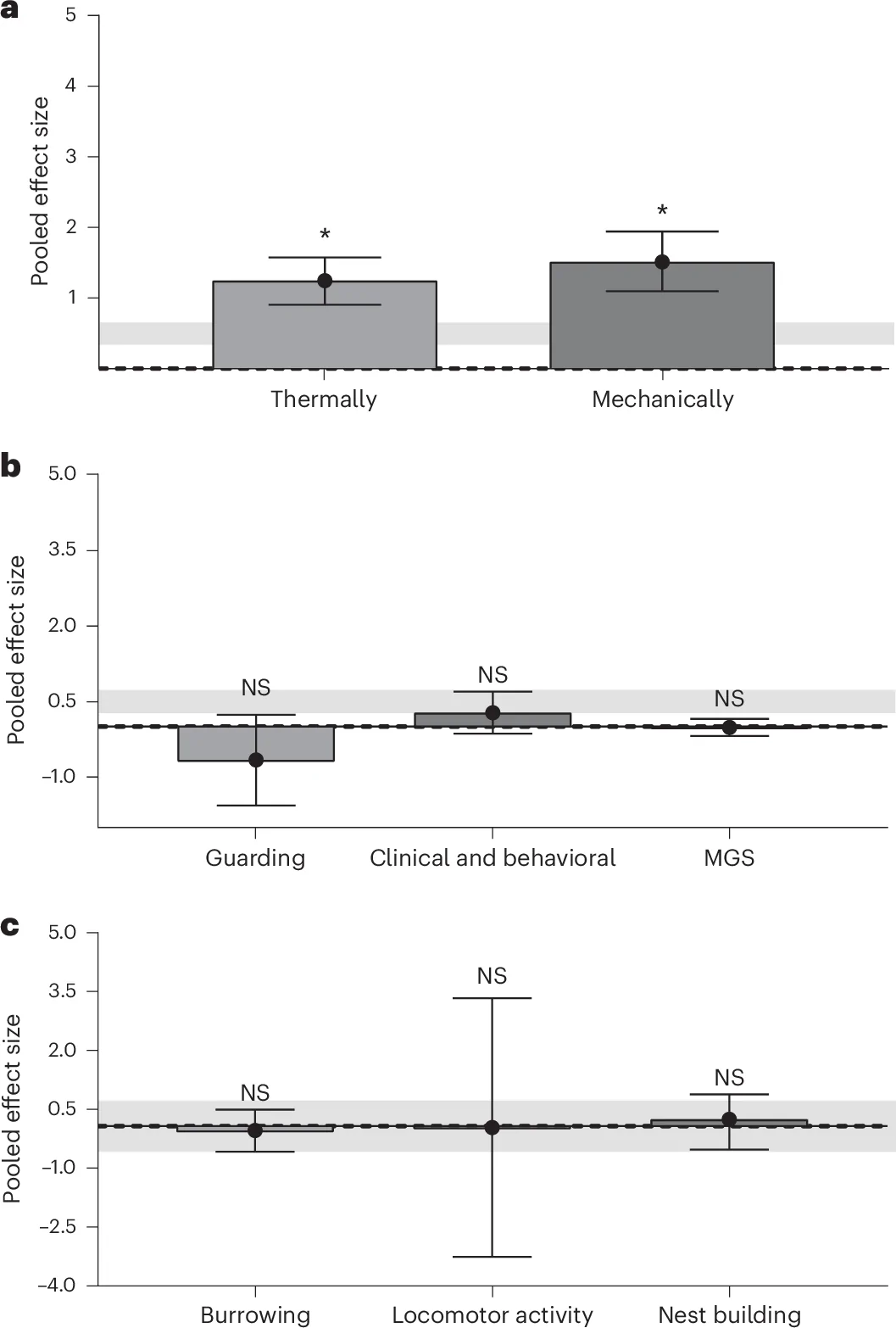
Summary of the pooled effect size of studies included in the meta-analysis of all analgesic treatments in different tests assessing pain modalities. Represented by SMD and 95% CI; the gray area represents the overall effect. **a**, Tests assessing thermally or mechanically evoked pain. **b**, Tests assessing spontaneous pain (guarding, clinical and behavioral investigation, and MGS). **c**, Tests assessing behaviors suppressed by pain (burrowing, locomotor activity and nest building). Asterisks indicate statistically significant differences between subgroups (*P* < 0.05, Bonferroni-adjusted test). Source data

We also investigated the differences between all analgesic treatments concerning the pain measurement modalities, which were divided into evoked pain, spontaneous behaviors and behaviors suppressed by pain (Fig. 4c and Supplementary Fig. 4). The SMD of the treatment effect was 1.12 (0.83 to 1.41), 0.12 (−0.03 to 0.27) and 0.06 (−0.16 to 0.28) when studies investigated evoked, spontaneous and pain suppressed behaviors, respectively, with high (*I*^2^ = 79%), mild (*I*^2^ = 29) and no (*I*^2^ = 0%) heterogeneity. The treatment effect was significant only for evoked pain, and significant differences were detected among the subgroups, with high heterogeneity (*Z* = 6.46; *P* < 0.001; *I*^2^ = 95%). Regarding the stimulus of evoked pain, the treatment produced similar analgesic effects against both thermally (Fig. 5a) (SMD 1.25 (0.91 to 1.58); *I*^2^ = 54%) and mechanically (SMD 1.52 (1.10 to 1.95), *I*^2^ = 84%) evoked pain (*Z* = 8.87; *P* < 0.001; *I*^2^ = 0.3%; Supplementary Fig. 5). We observed a similar absence of analgesic effect among the submodalities of spontaneous or suppressed behaviors (Fig. 5b,c and Supplementary Figs. 6 and 7). Together, the pain modalities assessed in different studies may explain the efficacy of analgesic treatments and the potential cause of overall heterogeneity.

### Analgesic classes

Different analgesic treatments were divided into opioids, NSAIDs, acetaminophen, gabapentinoids, local anesthetics and multimodal therapy (Fig. 4d and Supplementary Fig. 8). Treatment with opioids (SMD 0.65 (0.37 to 0.92); *I*^2^ = 76%), NSAIDs (SMD 0.33 (0.12 to 0.53); *I*^2^ = 43%), acetaminophen (SMD 0.47 (−0.11 to 0.83); *I*^2^ = 73%) and local anesthetics (SMD 1.42 (0.49 to 2.35; *I*^2^ = 69%)) produced significant analgesic effects. Meanwhile, the effect of gabapentinoids (SMD 2.16 (−0.72 to 5.04); *I*^2^ = 91%) and multimodal therapy (SMD −0.10 (−0.42 to 0.22) *I*^2^ = 93%) were not significant. However, differences were detected among the subgroups (*Z* = 6.42; *P* < 0.001). Regarding different opioid drugs, morphine treatment, but not buprenorphine and tramadol treatment, seemed to produce analgesic effects (Supplementary Fig. 9). We were unable to detect any significant differences among different NSAIDs tested (Supplementary Fig. 10). Bupivacaine and ropivacaine represent all the studies in the meta-analysis considering local anesthetic treatment. The ropivacaine treatment subgroup showed an effect size of 1.76 (0.98 to 2.54) (data not shown). However, only one study tested bupivacaine, and the effect size could not be calculated.

## Discussion

Effective management of pain in laboratory animals is required for advancing biomedical research. However, the identification of appropriate treatments is necessary to minimize animal distress^23^. Problems with postoperative analgesia involve efficacy (and safety)^24,25^. For example, NSAIDs and opioids are effective for treating POP in human patients, but their use is influenced by potential adverse effects and drug interactions^26,27^. These problems found in human patients may also occur in mice submitted to surgical procedures. In this meta-analysis, we evaluated the efficacy of analgesic treatments for POP in mice, addressing the influence of various factors, including sex, surgical invasiveness, pain modalities and analgesic classes. The meta-analysis of the analgesic treatments showed a positive POP-reducing effect in mice, considering pain assessment modalities, sex and surgical invasiveness.

A subgroup analysis based on sex indicated that the analgesic treatment effect was significant only in studies performed in male mice, indicating that sex was relevant to explaining the overall heterogeneity. In human patients, sex is well known to influence not only acute pain intensity (greater in women) but also the efficacy of different classes of analgesic drugs^28,29^. Regarding sex-based differences, the subgroup analysis showed varying treatment effects among male, female and mixed-sex studies, with significant analgesic effects observed primarily in male subjects. This shows the importance of considering sex as a factor in pain management strategies; therefore, studies performed in mice also indicate that sex is an important factor in POP occurrence^30–32^. A limitation of our results is that most studies were conducted in male mice and only a few compared sexes. It is a relevant point since many female mice are also submitted to surgical procedures in the laboratory, that is, ovariohysterectomy to study the influence of sexual hormones on different outcomes^33,34^. Considering that POP is a sex-dependent variable, more studies should evaluate the analgesic efficacy in POP using female mice^35,36^.

Similarly, the analgesic treatment effect was significant only with mild procedures, and differences were detected among the subgroups, indicating that the invasiveness of the surgical process explains the overall efficacy and heterogeneity. Similarly, in human patients, the invasiveness of surgical procedures impacts the intensity of pain and consequently the potential use of more effective analgesic drugs (that is, opioids)^37^. We previously categorized postoperative severity based on anatomical location, a detailed analysis of the tissue trauma and other intraoperative characteristics, which enables refinement actions and provides information for treatment of laboratory mice^38^. The invasiveness of surgical procedures in laboratory mice has a critical role in determining the need for human intervention (for example, analgesic administration and supportive care) to ensure effective pain management, minimizing the impact of untreated pain on research outcomes^13,38^.

Another limitation of our findings is that most of the studies we assessed involved minimally invasive procedures, with a particular emphasis on the paw incision model. The establishment of the POP model of incision in the mouse paw has considerably advanced the understanding of pain-like behaviors in mice^39^. The use of Brennan’s rodent paw incision model has contributed to the existing knowledge of POP^39^. Nevertheless, the paw incision model represents low invasiveness, with consequently low pain intensity^38^. Other surgeries, such as disease induction or medical device implantation, are more invasive and potentially more painful, changing the murine response to analgesics^40,41^. Therefore, it is important to consider the broader spectrum of surgical models and their implications for pain research and analgesic development^38^.

Pain assessment is a fundamental component of the effective safeguarding of animal welfare and practitioners working with animals require valid tools to assess pain to guide their pain management decisions^42,43^. However, the accuracy of pain assessments is directly dependent on their validity, ensuring the assessment is able to differentiate animals in pain from animals that are not experiencing pain or those experiencing other states (such as fear and anxiety)^42,43^. Of note, validated pain assessments are very limited for detecting POP in rodents. MGS has been partially validated for pain assessment post-surgery^32,44^. Moreover, suppression of nest building had strong convergent validity with MGS in operated mice^45^. From the 43 studies included in our meta-analysis, only 7 studies used MGS and 5 quantified nest building, indicating a clear limitation of the assessment of analgesic efficacy on POP in mice.

Spontaneous and evoked pain are two distinct types of pain experienced by humans and animals, each with its own relevance and implications for sensitivity to analgesics^38^. Another interesting result of our meta-analysis is that the analgesic effect is only observed in evoked pain behaviors but not in spontaneous pain-related behaviors or pain suppressed behaviors. One possible explanation for this result is the highest number of studies evaluating pain evoked by behaviors. In this context, the reliability of using solely stimuli-evoked methods for assessing pain behaviors in rodents has been questioned regarding their ability to mimic the spontaneous ongoing pain reported by many patients postoperatively^46^. Because mechanical or thermal stimuli induce spinal reflex withdrawal behavior, these methods probably represent an oversimplification of pain assessment^47^. Finally, we were unable to detect analgesic efficacy of POP-suppressed behavior (for example, nest building). Accordingly, another systematic review found controversial findings for the impact of analgesics on post-surgical nest building activity in rodents^48^. Altogether, these results emphasize the challenges in confirming analgesic efficacy in mice after surgeries and the need for different assessment methods to access POP in mice. We suggest that the evaluation of postsurgical pain should combine the assessment of different pain modalities, especially by validated methods.

We also detected that treatment with opioids, NSAIDs, acetaminophen and local anesthetics, but not gabapentinoids and multimodal therapy, produced significant analgesic effects in mice with POP. Accordingly, opioids, NSAIDs, acetaminophen and local anesthetics are also efficacious for treating POP in human patients^1^. Moreover, a systematic review and meta-analysis with human patients indicated no clinically significant analgesic effect of the perioperative use of gabapentinoids^49^. Despite evidence supporting the use of multimodal analgesic regimens to treat human patients with POP in many situations, the exact components of effective multimodal care will vary depending on the patient, setting and surgical procedure^1^. The reduced number of studies (6/48) that investigated multimodal analgesic therapy to treat POP in mice may explain the lack of efficacy of such therapy and is another limitation of our study. Finally, other factors influencing the efficacy of analgesic drugs include the patient’s sex^34,50^, the invasiveness of the procedure^38^ and the modality of pain evaluated, such as spontaneous or induced pain^51^.

Assessment of the quality and risk of bias of the included studies revealed that incomplete reporting limited the assessment of study quality and its impact on the magnitude of SMDs. The literature analyzed for this systematic review suggests that the distribution of SMD close to zero (no effect) and the inclusion of some high values for positive or weakly positive effects imply the underreporting of negative results^52,53^. In fact, traditional quality items, such as blinding, randomization and calculation of sample size, were rarely reported and failed to explain differences in the effect of analgesic treatment. Repeated observations are typically reported in each preclinical study and a single control group is often used in more than one comparison, thus reducing the use of animals. However, the inclusion of multiple observations per group and study may have been a limiting factor in our study and may have introduced bias during the meta-analysis. Another limitation of our work is that preclinical studies often did not report data in numerical format, necessitating the use of digital ruler software to estimate the reported data. The adhesion to the recommendations of the Animal Research: Reporting In Vivo Experiments (ARRIVE) guidelines may also improve this point in further studies on analgesic efficacy conducted in mice^54^. Furthermore, the limited number of articles is another limitation of our study and may influence the meta-analysis in some subgroups. Finally, the doses of the drugs used in our meta-analysis could also impact their effectiveness. Of note, all the doses of analgesic drugs tested in the included studies are within the dose range presented in the current guidelines to treat pain in laboratory mice^16–20^.

One relevant limitation of our meta-analysis could be the use of the classical random effect model. If several comparisons come from the same study, it is highly likely that the heterogeneity between these comparisons is smaller than the ones coming from other studies as methodological procedures are almost the same (same surgeon, for example). To address this point, we also submitted our data to a multilevel meta-analysis model^55^. In fact, the between-study variance (*σ*^2^ = 0.94) indicated moderate-to-high heterogeneity, whereas the within-study variance component was negligible (*σ*^2^ ≈ 0), suggesting that comparisons within the same study were highly consistent with each other. However, the results showed a significant overall effect (Hedges’ *g* = 0.57, 95% CI 0.26 to 0.89, *P* < 0.001), consistent in direction and significance with the conventional random-effects model (Hedges’ *g* = 0.46, 95% CI 0.31 to 0.60). Finally, we have also obtained similar results between random and multilevel analyses not only for the overall effect but also for the effects of subanalysis (data not shown). Thus, both random and multilevel analyses indicate a significant positive analgesic treatment effect, supporting the robustness of our conclusions.

## Conclusion

Surgical procedures are routinely conducted in mice, but the use of analgesic drugs is underreported and the assessment of their efficacy is limited. Effective management of pain in laboratory animals is indispensable for advancing biomedical research. However, the identification of appropriate treatments is necessary to minimize animal distress^23^. The use of analgesics following experimental procedures aims to aid researchers in making decisions regarding POP treatment with more robust evidence of their analgesic effectiveness, providing ethical guidelines for enhancing the refinement of experimental models and improving patient outcomes.

Our systematic review and meta-analysis assessed the influence of various factors, including sex, surgical invasiveness, pain modalities and analgesic classes used in experimental surgery. Taken together, our results indicate that, although analgesic drugs can reduce POP in mice, their efficacy is reduced in females, in moderately invasive surgeries and in spontaneous pain. Thus, high-quality studies are still needed to address ethical concerns and to guarantee full analgesia in laboratory mice submitted to surgical procedures.

## Methods

### Protocol registration

In this systematic review, we focused on the effect of POP analgesic efficacy in mice, assessed by different pain modality evaluation outcomes after surgery and treatment. We followed the PRISMA guidelines and the Systematic Review Center for Laboratory Animal Experimentation (SYRCLE) protocol. The study protocol was prospectively registered on the open science website under the identifier doi:10.17605/OSF.IO/B4DS3 (https://osf.io/b4ds3). No ethical committee approval was necessary for this review.

### Search strategy

We conducted a structured literature search to identify studies that explored POP in mice and the effect of analgesic treatments, with evidence of pain outcome evaluation (for example, evoked pain test or spontaneous pain behavior). A systematic search was performed in two databases, PubMed and Scopus, in May to July (2022). The search strategy included combining keywords from the Medical Subject Heading (MeSH) database and interplay between patient characteristics (operated mice), intervention (administration of established analgesics) and outcome (assessment of treatment effectiveness through pain behavior evaluation). The full search strategy is provided in Table 1.

Following the removal of duplicate entries using the Rayyan website, we initiated the process of screening the titles and abstracts of identified studies within the databases. Subsequently, the full text was obtained and independently assessed for eligibility by two members of the review team (R.G.S. and P.R.). Any disagreement between them over the eligibility of studies was resolved through discussion with a third reviewer (J.F.). The manual search was made by reading the full text of selected articles and reviewing the reference list to identify articles not found in the original search (P.R.).

### Inclusion and exclusion criteria

To identify relevant studies, we retrieved titles and/or abstracts using a designated search strategy and screened additional sources independently by two review authors by following the PICO elements. Population (P): we included studies involving animals subjected to a pain model of operative origin. Studies involving different species or models not of operative origin were excluded. Intervention (I): we considered studies that assessed pain management interventions. Studies that did not assess or describe pain management were excluded. Comparison (C): we included studies with controlled designs. Studies that were not controlled or used controls from another study were excluded. Outcome (O): we focused on studies reporting quantitative data on pain assessment. Studies with insufficient quantitative data, those that did not report the sample number, or those did not comply with ethical principles and ARRIVE guidelines were excluded. Additional limitations included only English-language studies or articles published after 2002 with full-text availability. Case reports, reviews, congress abstracts, preprints, book chapters and studies with insufficient quantitative data were also excluded.

### Quality assessment and publication bias

To understand all possible forms of bias, the studies included in the qualitative analysis were evaluated according to the Syrcle RoB tool^96^. The articles were categorized into selection, detection and interpretation bias; reporting bias; and other sources of bias. The risk of bias was assessed by two independent reviewers (R.G.S. and P.R.), and the differences were solved by consensus or a third reviewer (J.F.). To address specific concerns about methodological reporting in the studies and based in the literature^96^, we also added three additional reporting questions: whether randomization was reported in any level (level unknown); whether blinding was reported in any level (level unknown); and whether a power calculation was reported (yes/no).

### Data extraction

The values of the analgesic tests used to assess pain modalities and related outcome types were extracted with treatment, baseline and control data and are presented in Table 2. The graphical data were obtained using a digital ruler, WebPlotDigitizer (RRID: SCR_013996). When necessary, standard errors of the mean were transformed into standard deviations. When the animal group size was reported as a range, a conservative approach was chosen, and the lowest number of animals was used in the analysis. Articles that described multiple independent experiments in different groups of animals were included as independent comparisons. When a single study contained a number of comparisons, we corrected the number of animals reported by dividing the reported number by the number of treatment groups served to give a ‘true number of control animals’^97^. In the case of repeated measures, the largest effect size was selected for each comparison. In cases where a specific outcome was assessed by different methods, all the necessary data were collected for outcome comparison and to explore all pain modalities. If the study presented more than one method to evaluate analgesic efficacy, we collected data from all pain evaluative tests. Data extraction for the meta-analysis was also conducted by two independent reviewers (R.G.S. and P.R.).

### Data synthesis

Treatment types were categorized by the guidelines for the assessment and management of pain in rodents^16^, such as opioids, typical and atypical NSAIDs, gabapentinoids and local anesthetics. Administration was listed as single (1), double (2) or multiple (>2), and the route of administration was listed as IP, oral, SC, infiltrative, intravenous or nerve block. The times evaluated in the acute behavioral tests, from 0 to 72 h, were considered for the meta-analysis. The methodology used to assess pain was categorized into modalities, described by assessment types as evoked, spontaneous, or behaviors suppressed by pain, as shown in Table 2. Postoperative severity was categorized as mild, moderate and severe following the WWHow concept^38^.

The data were synthesized in this Article using a qualitative systematic approach (Supplementary Table 1), evaluating the effectiveness of the treatments and duration of the analgesic effect per the risk of bias (Fig. 2). For the meta-analysis, the treatments were subcategorized according to strain, class, invasiveness of the surgical procedure, and sex. The characteristics of the studies included comparisons according to the treated and control groups of mice undergoing surgery and the analgesic effect of the treatment over a specific period. Studies with a high risk of bias were excluded from the meta-analysis. For a study to be classified as having a high risk of bias, it needed to be rated as high risk in at least 12 out of the 13 criteria evaluated by the reviewers^97^.

### Data analysis

The forest plot graphics were used to obtain a general indication of the efficacy of POP relief, and it initially pooled all behavioral pain experiments (referred to as pain modalities) and all treatment outcomes regardless of surgery type or modality. The difference between treatments was used in the analysis as a SMD using the random-effects model^97^. Specifically, we used the SMD with the inverse variance method, which provides a pooled estimate of the effect size and its 95% CI. Tau squared was calculated using the random-effects model in RevMan 5.4 by estimating the variance between studies and quantifying heterogeneity. The forest plot represented the mean of the behavior test result, the standard deviation and the total number of animals in each group. The subanalyses included in this review were the comparison of sex to determine the influence of sex on POP analgesic response, pain severity following previous classification^38^, pain modality of outcome and analgesic type. For each study, Hedges *g* and 95% CIs were calculated and presented in a forest plot. We used the *I*^2^ statistic to classify the heterogeneity as no heterogeneity (<25%), mild heterogeneity (25–50%), moderate heterogeneity (50–75%) and high heterogeneity (>75%)^98^. The results of the subgroup analyses were interpreted only through forest plots or pooled effect sizes and were presented graphically when necessary. Statistical significance was set at *P* < 0.05. For subgroup analyses, we adjusted our significance level according to the conservative Bonferroni method to account for multiple analyses (*P* × number of comparisons) performed in RStudio software for Windows^98,99^ (RRID: SCR_000432). The meta-analysis was performed using Cochrane’s Review Manager (RevMan), Version 5.4 for Windows^100^ (RRID: SCR_003581).

## Online content

Any methods, additional references, Nature Portfolio reporting summaries, source data, extended data, supplementary information, acknowledgements, peer review information; details of author contributions and competing interests; and statements of data and code availability are available at 10.1038/s41684-026-01750-5.

## Supplementary information

Supplementary Information Supplementary Figs. 1–10 and Table 1 (full dataset).

Supplementary Data 1 Full dataset used for the multilevel meta analysis.

Supplementary Code 1 R script used for the multilevel meta analysis.

## Source data

Source Data Fig. 1–5 Source Data Fig. 1. Source data for PRISMA flow diagram. Source Data Fig. 2 Counts of studies rated as low, unclear or high risk of bias of reporting frequency for randomization, blinding and sample size calculation (Fig. 2a) and across all SYRCLE domains (Fig. 2b). Data correspond to the numeric values used to generate the bar plots in GraphPad Prism. Source Data Fig. 3. Statistical source data (SMD and SE for all studies included in the meta-analysis; same dataset used to generate the funnel plot). Source Data Fig. 4. Summary of pooled effect sizes across subgroup categories. This figure summarizes the SMDs and corresponding 95% CIs derived directly from the RevMan forest plots. The values plotted represent the pooled SMDs for each subgroup, sex (female, male, both/unspecified) (a), surgical severity (mild versus moderate) (b), pain modality (evoked, spontaneous, behaviors suppressed by pain) (c) and analgesic class (opioids, NSAIDs, acetaminophen, gabapentinoids, local anesthetics and multimodal) (d). No additional analyses were conducted outside RevMan; the graph illustrates only the numerical results obtained from the meta-analyses. Source Data Fig. 5. Summary of pooled effect sizes for all pain modality subanalyses. Numerical values (SMD and 95% CI) were extracted directly from RevMan forest plots and plotted in Prism for visualization. Modality-specific evoked tests (thermal versus mechanical) (a), spontaneous and clinically relevant behaviors (guarding, clinical/behavioral scoring and MGS) (b) and behaviors suppressed by pain (burrowing, locomotor activity and nest building) (c). No additional analyses were conducted outside RevMan; the graph illustrates only the numerical results obtained from the meta-analyses.

Source Data Supplementary Figs. 1–10 Dataset used to generate the forest plot summarizing the pooled SMDs and 95% CIs for all individual comparisons without subgroup stratification. Source Data Supplementary Fig. 2. Statistical source data for Extended Data Fig. 2. Dataset containing subgroup-level SMD calculations stratified by sex (female, male and both) for all analgesic treatments across all pain modalities. Source Data Supplementary Fig. 3. Dataset used to calculate SMDs stratified by surgical severity (mild versus moderate) including all analgesic treatments and pain modalities. Source Data Supplementary Fig. 4. Dataset containing pooled SMDs for all analgesic treatments grouped by pain modality (evoked, spontaneous and behaviors suppressed by pain). Source Data Supplementary Fig. 5. Dataset used to calculate pooled SMDs for all analgesic treatments evaluated under the evoked pain modality. Source Data Supplementary Fig. 6. Dataset used to calculate pooled SMDs for all analgesic treatments evaluated in spontaneous pain-like behaviors. Source Data Supplementary Fig. 7. Dataset used to calculate pooled SMDs for all analgesic treatments assessed using behaviors suppressed by pain. Source Data Supplementary Fig. 8. Dataset used to calculate pooled SMDs for all analgesic classes (opioids, NSAIDs, acetaminophen, gabapentinoids, local anesthetics and multimodal therapy) across all pain modalities. Source Data Supplementary Fig. 9. Dataset used to calculate pooled SMDs for all opioid treatments (buprenorphine, morphine, oxycodone, oxymorphone and tramadol). Source Data Supplementary Fig. 10. Dataset used to calculate pooled SMDs for all NSAID treatments (carprofen, meloxicam, flunixin, diclofenac, ibuprofen, ketoprofen and celecoxib).

## Data Availability

The data that support the findings of this study are available from the corresponding author upon request. Source data are provided with this paper.
