# Supplementary material for: Analgesic drug efficacy in mouse postoperative pain: a systematic review and meta-analysis
Source: Lab Anim (NY). 2026 Jun 23;55(9):334–43. doi: 10.1038/s41684-026-01750-5 (PMC13518225; doi:10.1038/s41684-026-01750-5)
Supplement: Supplementary file 1 [file 41684_2026_1750_MOESM1_ESM.pdf]

https://doi.org/10.1038/s41684-026-01750-5

Analgesic drug efficacy in mouse postoperative pain: a systematic review and meta-analysis

In the format provided by the authors and unedited

Study or Subgroup	Treatment Mean	Treatment SD	Treatment Total	Control Mean	Control SD	Control Total	Weight	Std. Mean Difference IV, Random, 95% CI
**1.1.1 All studies**								
ACE+LID+BUV (Durst, M. S. 2021) BR	0.28	1.3914	3	0.77	2.15	3	0.4%	-0.22 [-1.83, 1.40]
ACE+LID+BUV (Durst, M. S. 2021) BR	0.7	1.27	3	1.11	1.38	3	0.4%	-0.25 [-1.87, 1.37]
ACE+LID+BUV (Durst, M. S. 2021) MGS	101.81	187.83	3	63	203.1	3	0.4%	0.16 [-1.45, 1.77]
ACE+LID+BUV (Durst, M. S. 2021) MGS	101.2	153.3	3	68.7	156.8	3	0.4%	0.17 [-1.44, 1.78]
ACE+LID+BUV (Durst, M. S. 2021) NEST	0.58	5.8	3	0.59	6.3	3	0.4%	-0.00 [-1.60, 1.60]
ACE+LID+BUV (Durst, M. S. 2021) NEST	0.61	4.88	3	0.75	4.4	3	0.4%	-0.02 [-1.62, 1.58]
ACE100 (Cabañero & Maldonado. 2021) HT	4.65	1.35	10	3.58	1.69	10	0.6%	0.67 [-0.24, 1.58]
ACE100 (Cabañero & Maldonado. 2021) VF	0.58	0.21	10	0.38	0.14	10	0.6%	1.07 [0.12, 2.03]
ACE 100 (Matsumya L. C, et al. 2012) MGS	0.18	0.44	16	0.48	7.68	4	0.6%	-0.09 [-1.19, 1.01]
ACE120 (Zhu, Q. et al. 2016) VF	33.28	18.22	10	100	314	10	0.6%	-0.29 [-1.17, 0.59]
ACE200 (Cabañero & Maldonado. 2021) HT	6.3	2.94	10	3.58	1.69	10	0.6%	1.09 [0.13, 2.04]
ACE200 (Cabañero & Maldonado. 2021) VF	0.89	0.24	10	0.38	0.14	10	0.5%	2.49 [1.26, 3.71]
ACE200 (Durst, M. S. et al. 2021) BR	126.06	149.48	10	63.03	203.14	10	0.6%	0.34 [-0.55, 1.22]
ACE200 (Durst, M. S. et al. 2021) BR	90.36	180.6	8	68.67	156.75	8	0.6%	0.12 [-0.86, 1.10]
ACE200 (Durst, M. S. et al. 2021) MGS	0.63	6.3	10	0.73	6.3	10	0.6%	-0.02 [-0.89, 0.86]
ACE200 (Durst, M. S. et al. 2021) MGS	0.54	4.32	8	0.62	4.4	8	0.6%	-0.02 [-1.00, 0.96]
ACE200 (Durst, M. S. et al. 2021) NEST	0.52	1.55	10	0.77	2.15	10	0.6%	-0.13 [-1.01, 0.75]
ACE200 (Durst, M. S. et al. 2021) NEST	0.5	1.5	8	1.11	1.38	8	0.6%	-0.40 [-1.39, 0.59]
ACE240 (Zhu, Q. et al. 2016) VF	43.21	33.36	10	100	314	10	0.6%	-0.24 [-1.12, 0.64]
ACE30 (Zhu, Q. et al. 2016) VF	21.6	21.23	10	100	314	10	0.6%	-0.34 [-1.22, 0.55]
ACE300 (Cabañero & Maldonado. 2021) HT	6.02	2.29	10	3.58	1.69	10	0.6%	1.16 [0.20, 2.13]
ACE300 (Cabañero & Maldonado. 2021) VF	1.03	0.28	10	0.38	0.14	10	0.5%	2.81 [1.50, 4.12]
ACE 300 (Matsumya L. C, et al. 2012) MGS	0.37	0.72	16	0.48	7.68	4	0.6%	-0.03 [-1.13, 1.06]
ACE360 (Zhu, Q. et al. 2016) VF	60.14	29.34	10	100	314	10	0.6%	-0.17 [-1.05, 0.71]
ACE400 (Cabañero & Maldonado. 2021) HT	6.49	2.94	10	3.58	1.69	10	0.6%	1.16 [0.20, 2.13]
ACE400 (Cabañero & Maldonado. 2021) VF	1.28	0.21	10	0.38	0.14	10	0.3%	4.83 [2.94, 6.72]
ACE 450 (Matsumya L. C, et al. 2012) MGS	0.32	0.68	16	0.48	7.68	4	0.6%	-0.05 [-1.14, 1.05]
ACE50 (Cabañero & Maldonado. 2021) HT	3.2	2.01	10	3.58	1.69	10	0.6%	-0.20 [-1.08, 0.68]
ACE50 (Cabañero & Maldonado. 2021) VF	0.52	0.24	10	0.38	0.14	10	0.6%	0.68 [-0.23, 1.59]
ACE60 (Zhu, Q. et al. 2016) VF	28.32	37.41	10	100	314	10	0.6%	-0.31 [-1.19, 0.58]
BUP+CAR (Adamsom, T, W) 2010 RW	0.17	0.18	2	0.15	0.13	2	0.3%	0.07 [-1.93, 2.08]
BUP+CAR (Adamson, T. W. 2010) VS	1.4	0.55	2	1.15	0.37	2	0.3%	0.30 [-2.31, 2.92]
BUP+MEL+BUV (Furumoto, K. 2021) NEST	1.3	0.47	3	3.17	1.61	3	0.3%	-1.26 [-3.27, 0.75]
BUP0.001 (Matsumya L. C, et al. 2012) MGS	0.37	0.68	16	0.48	7.68	4	0.6%	-0.03 [-1.13, 1.06]
BUP0.009 (Jirkof, P. et al. 2019) MGS	3.85	15.4	8	1.28	12.4	4	0.5%	0.16 [-1.04, 1.37]
BUP0.009 (Jirkof, P. et al. 2019) MGS	4.8	19.2	8	2.06	19.04	4	0.5%	0.13 [-1.07, 1.33]
BUP0.01 (Matsumya L. C, et al. 2012) MGS	0.22	0.44	16	0.48	7.68	4	0.6%	-0.08 [-1.17, 1.02]
BUP0.01 (Wright-Williams, S. L. 2013) VS	1	0.79	8	4.97	2.26	8	0.4%	-2.22 [-3.54, -0.90]
BUP 0.024 (Faller, K.M.E, 2015)	3.17	1.48	13	1.39	0.58	13	0.6%	1.53 [0.64, 2.43]
BUP0.05 (Matsumya L. C, et al. 2012) MGS	0.18	2.88	16	0.48	7.68	4	0.6%	-0.07 [-1.17, 1.03]
BUP0.05 (Wright-Williams, S. L. 2013) VS	2	2.72	8	4.97	2.26	8	0.6%	-1.12 [-2.20, -0.05]
BUP0.1 (Cho, C. et al. 2019) MGS	0.18	1.44	8	0.42	3.36	8	0.6%	-0.09 [-1.07, 0.89]
BUP0.1 (Cho, C. et al. 2019) MGS	0.13	1.04	8	0.38	3.04	8	0.6%	-0.10 [-1.08, 0.88]
BUP0.1 (Cho, C. et al. 2019) MGS	0.07	0.56	8	0.28	2.24	8	0.6%	-0.12 [-1.10, 0.86]
BUP0.1 (Cho C. et al. 2019) MGS	0.04	0.32	8	0.25	2	8	0.6%	-0.14 [-1.12, 0.84]
BUP0.1 (Furumoto, K. et al. 2021) NEST	1.3	0.47	7	3.17	1.61	7	0.5%	-1.48 [-2.71, -0.25]
BUP0.1 (Jirkof, P. et al. 2015) BR	3.02	1.33	8	10.3	26.3	8	0.6%	-0.37 [-1.36, 0.62]
BUP0.1 (Jirkof, P. et al. 2015) VS	0.37	0.76	8	0.88	0.84	8	0.6%	-0.60 [-1.61, 0.41]
BUP0.1 (Matsumya L. C, et al. 2012) MGS	0.11	1.76	16	0.48	7.68	4	0.6%	-0.10 [-1.20, 1.00]
BUP0.1(Oliver V; Thurston S; Logfren J. 2018) VF	4.31	1.35	8	3.05	1.39	8	0.6%	0.87 [-0.17, 1.91]
BUP0.1-0.15(MolinaCimadevila M. J. et al. 2014) HT	13.76	29.75	6	63	199	6	0.6%	-0.32 [-1.46, 0.82]
BUP0.1-0.3(MolinaCimadevila, M. J. et al. 2014) HT	35.2	36.25	6	63	199	6	0.6%	-0.18 [-1.31, 0.96]
BUP0.1 HCl (Kendal, L. V. et al. 2016) NEST	69.3	41.5	7	62.9	46.8	7	0.6%	0.14 [-0.91, 1.18]
BUP0.1 HCl (Kendal, L. V. et al. 2016) RW	21.1	26.1	7	43.2	33.2	7	0.6%	-0.69 [-1.78, 0.40]
BUP0.1 HCl (Kendal, L. V. et al. 2016) VS	1.25	0.5	7	1	0.7	7	0.6%	0.38 [-0.68, 1.45]
BUP0.2 (Adamson, T. W. et al. 2010) RW	0.5	0.87	7	0.15	0.13	7	0.6%	0.53 [-0.55, 1.60]
BUP0.2 (Adamson, T. W. et al. 2010) VS	1.71	1.08	7	1.15	0.37	7	0.6%	0.65 [-0.44, 1.73]
BUP0.2 (Clark, M. D. et al. 2004) RW	46.71	32.82	10	34.6	10.6	6	0.6%	0.42 [-0.60, 1.45]
BUP0.2 (Clark, M. D. et al. 2004) VS	46.71	32.82	10	34.6	10.6	6	0.6%	0.42 [-0.60, 1.45]
BUP0.2 (Reddyjarugu, B. et al. 2015) HT	4.76	4.35	10	2.97	1.66	9	0.6%	0.51 [-0.41, 1.43]
BUP0.2 (Reddyjarugu, B. et al. 2015) VF	2.83	0.91	10	1.1	3.12	9	0.6%	0.74 [-0.20, 1.68]
BUP0.6 (Kendal, L. V. et al. 2016) NEST	94.1	8.6	6	62.8	46.8	7	0.6%	0.83 [-0.32, 1.99]
BUP0.6 (Kendal, L. V. et al. 2016) RW	9.5	16.1	6	43.2	33.2	7	0.5%	-1.17 [-2.39, 0.05]
BUP0.6 (Kendal, L. V. et al. 2016) VS	1.7	0.3	6	1	0.7	7	0.5%	1.17 [-0.04, 2.39]
BUP1.67x10^3(Lu, F. et al. 2022) VF	3.99	0.08	11	3.99	0.12	11	0.7%	0.00 [-0.84, 0.84]
BUP2 (Goecke, J. C. et al. 2005) VS	0.59	0.16	7	0.18	0.09	6	0.4%	2.87 [1.16, 4.58]
BUP2 (Goecke, J. C. et al. 2005) VS	0.38	0.27	6	0.18	0.09	6	0.5%	0.92 [-0.30, 2.13]
BUP-SR1 (Navarro K, et al. 2020) HT	6.45	3.76	12	3.22	5.29	8	0.6%	0.70 [-0.23, 1.63]
BUP-SR1 (Navarro K, et al. 2020) VF	5.35	2	12	6.26	3.07	8	0.6%	-0.35 [-1.26, 0.55]
BUP-SR2.2 (Jirkof, P. et al. 2015) BR	1.32	1.95	8	0.88	0.84	8	0.6%	0.28 [-0.71, 1.26]
BUP-SR2.2 (Jirkof, P. et al. 2015) VS	4.08	2.57	8	10.3	26.3	8	0.6%	-0.31 [-1.30, 0.67]
BUP-XR3.25 (Navarro K, et al. 2020) HT	9.07	6.72	12	3.22	5.29	8	0.6%	0.90 [-0.04, 1.85]
BUP-XR3.25 (Navarro K, et al. 2020) VF	5.45	2.84	12	6.26	3.07	8	0.6%	-0.26 [-1.16, 0.63]
BUP-XR6.25 (Navarro K, et al. 2020) HT	8.47	3.76	12	3.22	5.29	8	0.6%	1.14 [0.16, 2.12]
BUP-XR6.25 (Navarro K, et al. 2020) VF	6.29	2.28	12	6.26	3.07	8	0.6%	0.01 [-0.88, 0.91]
BUV5 (Leach, M. C. et al. 2012) MGS	1.99	11.94	6	3.86	23.16	6	0.6%	-0.09 [-1.23, 1.04]
CAR10 (Cho, C. et al. 2019) MGS	0.18	1.44	8	0.42	3.36	8	0.6%	-0.09 [-1.07, 0.89]
CAR10 (Cho, C. et al. 2019) MGS	0.24	1.92	8	0.38	3.04	8	0.6%	-0.05 [-1.03, 0.93]
CAR10 (Cho, C. et al. 2019) MGS	0.01	0.001	8	0.25	2	8	0.6%	-0.16 [-1.14, 0.82]
CAR10 (Cho, C. et al. 2019) MGS	0.17	1.36	8	0.28	2.24	8	0.6%	-0.06 [-1.04, 0.92]
CAR 10 (Matsumya L. C, et al. 2012) MGS	0.52	0.2	16	0.48	7.68	4	0.6%	0.01 [-1.08, 1.11]
CAR 15 (Matsumya L. C, et al. 2012) MGS	0.55	0.36	16	0.48	7.68	4	0.6%	0.02 [-1.07, 1.12]
CAR20 (Matsumya L. C, et al. 2012) MGS	0.07	1.12	16	0.48	7.68	4	0.6%	-0.12 [-1.22, 0.98]
CAR25 (Cho, C. et al. 2019) MGS	0.11	0.88	8	0.38	3.04	8	0.6%	-0.11 [-1.10, 0.87]
CAR25 (Cho, C. et al. 2019) MGS	0.09	0.72	8	0.28	2.24	8	0.6%	-0.11 [-1.09, 0.87]
CAR25 (Cho, C. et al. 2019) MGS	0.06	0.48	8	0.25	2	8	0.6%	-0.12 [-1.10, 0.86]
CAR25 (Cho, C. et al. 2019) MGS	0.06	0.48	8	0.25	2	8	0.6%	-0.12 [-1.10, 0.86]
CAR25 (Matsumya L. C, et al. 2012) MGS	0.15	2.4	16	0.48	7.68	4	0.6%	-0.08 [-1.18, 1.01]
CAR30 (Oliver V; Thurston S; Logfren J. 2018) VF	3.9	1.63	8	3.05	1.39	8	0.6%	0.53 [-0.47, 1.53]
CAR5 (Adamson, T. W. et al. 2010) RW	0.31	0.26	7	0.15	0.13	7	0.6%	0.73 [-0.37, 1.82]
CAR5 (Adamson, T. W. et al. 2010) VS	0.96	0.48	7	1.15	0.37	7	0.6%	-0.42 [-1.48, 0.65]
CAR5 (Jirkof, P. et al. 2010) BR	35	41	7	10.41	5.06	8	0.6%	0.82 [-0.25, 1.89]
CAR5 (Jirkof, P. et al. 2012) VS	248.38	110.34	8	191.93	136.44	8	0.6%	0.43 [-0.56, 1.43]
CAR5 (Jirkof, P. et al. 2013) NEST	0.04	0.23	8	0.1	0.002	8	0.6%	-0.35 [-1.34, 0.64]
CAR 5 (Matsumya L. C, et al. 2012) MGS	0.43	0.4	16	0.48	7.68	4	0.6%	-0.02 [-1.11, 1.08]
CAR50 (Jirkof, P. et al. 2013) NEST	0.68	1.27	8	0.1	0.002	8	0.6%	0.61 [-0.40, 1.62]
CEL1 (Muratani T. et al. 2005) VF	1.43	0.24	6	1.19	0.13	5	0.5%	1.10 [-0.22, 2.42]
CEL1 (Muratani T. et al. 2005) VF	5.65	2.34	6	5.89	3.24	6	0.6%	-0.08 [-1.21, 1.05]
CEL10 (Muratani T. et al. 2005) VF	5.96	1.62	6	5.89	3.24	6	0.6%	0.03 [-1.11, 1.16]
CEL10 (Muratani T. et al. 2005) VF	6.2	3	6	8.58	3.9	6	0.5%	-0.63 [-1.80, 0.54]
DEX100 (Romero-Alejo, E. et al. 2016) VF	0.9	0.11	10	0.36	0.18	10	0.4%	3.47 [1.98, 4.95]
DIC0.25 (Lu, F. et al. 2022) VF	3.84	0.69	11	3.99	0.12	11	0.7%	-0.29 [-1.13, 0.55]
DIC0.25 (Lu, F. et al. 2022) VF	1.29	0.8	11	1.57	1.56	11	0.7%	-0.22 [-1.06, 0.62]
FIR10 (Reddyjarugu, B. et al. 2015) HT	5.59	4.61	9	2.97	1.66	9	0.6%	0.72 [-0.24, 1.68]
FIR10 (Reddyjarugu, B. et al. 2015) VF	2.41	1.39	9	1.1	3.12	9	0.6%	0.52 [-0.43, 1.46]
FIR20 (Reddyjarugu, B. et al. 2015) HT	7.14	3.91	10	2.97	1.66	9	0.6%	1.30 [0.29, 2.31]
FIR20 (Reddyjarugu, B. et al. 2015) VF	2.24	0.47	10	1.1	3.12	9	0.6%	0.50 [-0.42, 1.42]
FLU2.5 (Goecke, J. C. et al. 2005) VS	0.23	0.13	7	0.18	0.09	6	0.6%	0.41 [-0.70, 1.52]
FLU2.5 (Goecke, J. C. et al. 2005) VS	0.27	0.15	6	0.18	0.09	6	0.5%	0.67 [-0.51, 1.85]
GABA20 (Yang J. et al. 2019) VF	0.28	0.15	10	0.05	0.03	9	0.5%	1.98 [0.83, 3.12]
GABA50 (Yang J. et al. 2019) VF	0.53	0.08	6	0.05	0.03	5	0.1%	6.98 [3.15, 10.81]
IBU100 (Saad, S. S. T. et al. 2016) VF	2.58	0.97	9	0.74	0.46	8	0.5%	2.25 [0.97, 3.54]
IBU300 (Saad, S. S. T. et al. 2016) VF	2.98	2.29	9	0.74	0.46	8	0.6%	1.25 [0.18, 2.32]
INDO1 (Brusco, I. et al. 2017) VF	3.23	0.79	6	1.44	1.27	6	0.5%	1.56 [0.20, 2.93]
INDO10 (Brusco, I. et al. 2017) SP	5.5	21.9	6	8.94	6.89	6	0.6%	-0.20 [-1.33, 0.94]
INDO10 (Brusco, I. et al. 2017) SP	0.93	12.47	6	1.98	0.49	6	0.6%	-0.11 [-1.24, 1.02]
INDO10 (Brusco, I. et al. 2017) VF	4.23	0.59	6	1.44	1.27	6	0.4%	2.60 [0.90, 4.30]
INDO10 (Brusco, I. et al. 2017) VF	4.3	0.48	6	1.77	1.07	6	0.4%	2.82 [1.04, 4.60]
INDO3 (Brusco, I. et al. 2017) VF	4.06	0.48	6	1.44	1.27	6	0.4%	2.52 [0.85, 4.19]
KET20 (Matsumya L. C, et al. 2012) MGS	0.19	3.04	16	0.48	7.68	4	0.6%	-0.07 [-1.16, 1.03]
KET50 (Minville, V. et al. 2008) HT	11.91	1.24	10	5.95	2.88	10	0.5%	2.57 [1.33, 3.82]
KET50 (Minville, V. et al. 2008) VF	3.99	7.34	10	1.03	1.83	10	0.6%	0.53 [-0.37, 1.43]
KETO 1 (Matsumya L. C, et al. 2012) MGS	0.53	0.56	16	0.48	7.68	4	0.6%	0.02 [-1.08, 1.11]
KETO 10 (Matsumya L. C, et al. 2012) MGS	0.64	0.6	16	0.48	7.68	4	0.6%	0.05 [-1.05, 1.14]
KETO 15 (Matsumya L. C, et al. 2012) MGS	0.18	0.44	16	0.48	7.68	4	0.6%	-0.09 [-1.19, 1.01]
KETO 5 (Matsumya L. C, et al. 2012) MGS	0.43	0.2	16	0.48	7.68	4	0.6%	-0.02 [-1.11, 1.08]
LID+BUV (Durst, M. S. 2021) BR	130.03	203.14	3	0.63	103.1	3	0.4%	0.64 [-1.07, 2.36]
LID+BUV (Durst, M. S. 2021) BR	26.5	95.43	3	68.7	156.8	3	0.4%	-0.26 [-1.88, 1.36]
LID+BUV (Durst, M. S. 2021) MGS	0.41	4.1	3	0.58	6.3	3	0.4%	-0.03 [-1.63, 1.57]
LID+BUV (Durst, M. S. 2021) MGS	0.26	2.08	3	0.35	4.4	3	0.4%	-0.02 [-1.62, 1.58]
LID+BUV (Durst, M. S. 2021) NEST	0.89	2.18	3	0.77	2.15	3	0.4%	0.04 [-1.56, 1.65]
LID+BUV (Durst, M. S. 2021) NEST	0.4	1.41	3	1.11	1.38	3	0.4%	-0.41 [-2.06, 1.24]
MEL+BUP (Molina-Cimadevila, M. J. 2014) HT	35.36	37.48	2	63	199	2	0.3%	-0.11 [-2.17, 1.95]
MEL10 (Roughan, V. J. et al. 2009) VS	24.27	26.33	8	10.97	27.26	8	0.6%	0.47 [-0.53, 1.47]
MEL10 (Roughan, V. J. et al. 2016) VS	6.37	4.48	8	3.76	5.13	9	0.6%	0.51 [-0.46, 1.48]
MEL2(Cho, C. et al. 2019) MGS	0.01	0.04	8	0.25	2	8	0.6%	-0.16 [-1.14, 0.82]
MEL2 (Cho, C. et al. 2019) MGS	0.11	0.88	8	0.42	3.36	8	0.6%	-0.12 [-1.10, 0.86]
MEL2 (Cho, C. et al. 2019) MGS	0.14	1.12	8	0.38	3.04	8	0.6%	-0.10 [-1.08, 0.88]
MEL2 (Cho, C. et al. 2019) MGS	0.08	0.64	8	0.38	3.04	8	0.6%	-0.13 [-1.11, 0.85]
MEL20 (Leach, M. C. et al. 2012) MGS	2.35	14.1	6	3.86	23.16	6	0.6%	-0.07 [-1.20, 1.06]
MEL20 (Roughan, V. J. et al. 2009) VS	16.95	14.11	8	10.97	27.26	8	0.6%	0.26 [-0.72, 1.25]
MEL20 (Wright-Williams, S. L. 2009) VS	8.5	9.8	8	17.1	30.8	8	0.6%	-0.36 [-1.35, 0.63]
MEL5 (Cho, C. et al. 2019) MGS	0.2	1.6	8	0.42	3.36	8	0.6%	-0.08 [-1.06, 0.90]
MEL5 (Cho, C. et al. 2019) MGS	0.08	0.64	8	0.38	3.04	8	0.6%	-0.13 [-1.11, 0.85]
MEL5 (Cho, C. et al. 2019) MGS	0.06	0.48	8	0.28	2.24	8	0.6%	-0.13 [-1.11, 0.85]
MEL5 (Cho, C. et al. 2019) MGS	0.12	0.96	8	0.25	2	8	0.6%	-0.08 [-1.06, 0.90]
MEL5 (Roughan, V. J. et al. 2009) VS	50.54	20.7	8	10.97	27.26	8	0.5%	1.55 [0.39, 2.70]
MOR1 (Clark, J. D. et al. 2007) VF	2.47	0.72	5	0.22	0.26	5	0.2%	3.75 [1.30, 6.20]
MOR1 (Dallazen, J. L et al. 2022) HT	12.27	3.74	6	4.48	1.35	6	0.4%	2.56 [0.87, 4.24]
MOR1 (Dallazen, J. L et al. 2022) SP	6.01	16.9	6	6.01	16.04	6	0.6%	0.00 [-1.13, 1.13]
MOR1 (Dallazen, J. L et al. 2022) VF	0.97	0.28	8	0.04	0.16	8	0.4%	3.86 [2.03, 5.68]
MOR10 (Clark, J. D. et al. 2007) VF	0.66	0.18	5	0.22	0.26	5	0.4%	1.78 [0.18, 3.37]
MOR10 (Minville, V. et al. 2008) HT	11.95	0.88	10	5.95	2.88	10	0.5%	2.70 [1.42, 3.98]
MOR10 (Minville, V. et al. 2008) VF	8	0.91	10	1.03	1.83	10	0.4%	4.62 [2.80, 6.44]
MOR10 (Pogatzki, E. M. & Raja, S. N. 2003) HT	13.27	7.83	15	1.89	1.13	9	0.6%	1.75 [0.76, 2.74]
MOR10 (Pogatzki, E. M & Raja, S. N. 2003) VF	7.81	0.001	15	2.16	7.35	15	0.7%	1.06 [0.29, 1.83]
MOR10 (Tonello, R. et al. 2020) SP	0.14	0.29	6	7.63	2.94	6	0.3%	-3.31 [-5.28, -1.34]
MOR10 (Tonello, R. et al. 2020) VF	7.99	0.49	6	2.7	0.91	6	0.1%	6.68 [3.23, 10.13]
MOR10* (Wei, S. et al. 2021) VF	5.52	0.95	8	0.32	0.35	8	0.2%	6.87 [3.96, 9.78]
MOR3 (Minville, V. et al. 2008) HT	11.96	1.06	10	5.95	2.88	10	0.5%	2.65 [1.38, 3.92]
MOR3 (Minville, V. et al. 2008) VF	3.98	6.65	10	1.03	1.83	10	0.6%	0.58 [-0.32, 1.48]
MOR3 (Pogatzki, E. M. & Raja, S. N. 2003) HT	6.33	3.42	15	1.89	1.13	9	0.6%	1.52 [0.57, 2.48]
MOR3 (Tonello, R. et al. 2020) SP	2.62	5.68	6	7.63	2.94	6	0.5%	-1.02 [-2.26, 0.21]
MOR3 (Tonello, R. et al. 2020) VF	6.05	1.3	6	2.7	0.91	6	0.4%	2.76 [1.00, 4.51]
MOR3* (Wei, S. et al. 2021) VF	2.79	0.44	8	0.32	0.35	8	0.2%	5.87 [3.33, 8.41]
MOR5* (Wei, S. et al. 2021) VF	3.49	1.52	8	0.32	0.35	8	0.5%	2.72 [1.26, 4.18]
OXY3 (Mueller, A. et al. 2019) VF	1.43	0.24	6	1.19	0.13	5	0.5%	1.10 [-0.22, 2.42]
OXYM-LE 2 (Clark, M. D. et al. 2004) VS	104.67	27.35	10	34.6	10.6	6	0.4%	2.90 [1.36, 4.44]
PRE30 (Park, J. H et al. 2020) VF	6.5	4	8	8.59	2.12	8	0.6%	-0.62 [-1.63, 0.39]
ROP5% (Yamada, T. et al. 2016) HT	18.11	7.21	9	10.54	3.22	9	0.6%	1.29 [0.25, 2.33]
ROP5% (Yamada, T. et al. 2016) HT	20.4	2.88	9	9.77	3.61	9	0.4%	3.10 [1.63, 4.57]
ROP5% (Yamada, T. et al. 2016) VF	1.97	0.42	9	0.16	1.15	9	0.5%	1.99 [0.81, 3.17]
ROP5% (Yamada, T. et al. 2016) VF	1.88	1.95	9	0.1	0.76	9	0.6%	1.15 [0.13, 2.16]
TRA+ACE (Jirkof, P. 2018) MGS	2.3	18.4	8	3.1	24.8	8	0.6%	-0.03 [-1.01, 0.95]
TRA+ACE (Jirkof, P. 2018) NEST	2.97	1.32	8	2.57	0.84	8	0.6%	0.34 [-0.65, 1.33]
TRA+ACE (Jirkof, P. 2018) VS	3.36	2.17	8	4.54	2	8	0.6%	-0.53 [-1.54, 0.47]
TRA+ACE (Jirkof, P. 2018) VS	2.35	1.89	8	3.86	3.1	8	0.6%	-0.56 [-1.56, 0.45]
TRA+DEXK (Romero-Alejo, E. 2016) VF	0.82	0.14	3	0.36	0.183	3	0.2%	2.26 [-0.45, 4.96]
TRA0.1 (Jirkof, P. et al. 2019) MGS	3.2	12.8	8	3.49	15.2	4	0.5%	-0.02 [-1.22, 1.18]
TRA0.1 (Jirkof, P. et al. 2019) MGS	2.93	11.72	8	2.21	14.72	4	0.5%	0.05 [-1.15, 1.25]
TRA1 (Jirkof, P. et al. 2019) MGS	3.66	14.64	8	3.22	10.08	4	0.5%	0.03 [-1.17, 1.23]
TRA1 (Jirkof, P. et al. 2019) MGS	3	12	8	3.18	19.36	4	0.5%	-0.01 [-1.21, 1.19]
TRA100 (Romero-Alejo, E. et al. 2016) VF	1.14	0.15	10	0.36	0.18	10	0.4%	4.51 [2.72, 6.30]
TRA25 (Evangelista-Vaz, R. et al. 2018) BR	2.13	3.42	8	3.64	6.05	8	0.6%	-0.29 [-1.28, 0.70]
TRA25 (Evangelista-Vaz, R. et al. 2018) VS	214	383.82	8	191	156.41	8	0.6%	0.07 [-0.91, 1.05]
TRA80 (Wolfe, A. M. et al. 2015) VS	0.7	0.66	10	1.65	0.79	10	0.6%	-1.25 [-2.23, -0.27]
**Subtotal (95% CI)**			**1602**			**1319**	**100.0%**	**0.46 [0.31, 0.60]**

Heterogeneity: Tau² = 0.64; Chi² = 561.39, df = 185 (P < 0.00001); I² = 67%
Test for overall effect: Z = 6.20 (P < 0.00001)

**Total (95% CI)**			**1602**			**1319**	**100.0%**	**0.46 [0.31, 0.60]**

Heterogeneity: Tau² = 0.64; Chi² = 561.39, df = 185 (P < 0.00001); I² = 67%
Test for overall effect: Z = 6.20 (P < 0.00001)
Test for subgroup differences: Not applicable

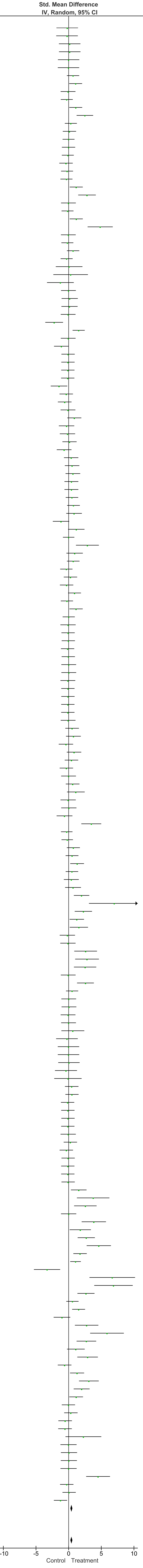

Supplementary figure 1. Summary of the effect size of all studies included in the meta analysis without subgroup analysis. Forest plot presenting the pooled standardized mean difference (SMD) and 95% confidence intervals (CI) for all individual comparisons included in the meta-analysis, without subgroup stratification.

Study or Subgroup	Treatment Mean	Treatment SD	Treatment Total	Control Mean	Control SD	Control Total	Weight	Std. Mean Difference IV, Random, 95% CI
**1.2.1 Male**								
ACE50 (Cabañero & Maldonado. 2021) HT	6.49	2.94	10	3.58	1.69	10	0.6%	1.16 [0.20, 2.13]
ACE50 (Cabañero & Maldonado. 2021) VF	1.28	0.2	10	0.38	0.13	10	0.3%	5.11 [3.14, 7.08]
ACE100 (Cabañero & Maldonado. 2021) HT	3.2	2	10	3.58	1.69	10	0.6%	-0.20 [-1.08, 0.68]
ACE100 (Cabañero & Maldonado. 2021) VF	0.52	0.24	10	0.38	0.13	10	0.6%	0.69 [-0.21, 1.60]
ACE200 (Cabañero & Maldonado. 2021) HT	4.65	1.351	10	3.58	1.69	10	0.6%	0.67 [-0.24, 1.58]
ACE200 (Cabañero & Maldonado. 2021) VF	0.58	0.2	10	0.38	0.138	10	0.6%	1.11 [0.16, 2.07]
ACE200 (Durst, M. S. et al. 2021) MGS	0.63	6.3	10	0.73	6.3	10	0.6%	-0.02 [-0.89, 0.86]
ACE200 (Durst, M. S. et al. 2021) BR	126.06	149.48	10	63.03	203.14	10	0.6%	0.34 [-0.55, 1.22]
ACE200 (Durst, M. S. et al. 2021) NEST	0.52	1.54	10	0.77	2.15	10	0.6%	-0.13 [-1.01, 0.75]
BUP1.67x10³(Lu, F. et al. 2022) VF	3.99	0.08	11	3.99	0.12	11	0.7%	0.00 [-0.84, 0.84]
ACE300 (Cabañero & Maldonado. 2021) HT	6.3	2.94	10	3.58	1.69	10	0.6%	1.09 [0.13, 2.04]
ACE300 (Cabañero & Maldonado. 2021) VF	0.89	0.24	10	0.38	0.13	10	0.5%	2.53 [1.29, 3.77]
ACE400 (Cabañero & Maldonado. 2021) HT	6.02	2.28	10	3.58	1.69	10	0.6%	1.16 [0.20, 2.13]
ACE400 (Cabañero & Maldonado. 2021) VF	1.03	0.27	10	0.38	0.13	10	0.5%	2.94 [1.60, 4.28]
ACE+LID+BUV (Durst, M. S. 2021) BR	101.81	187.839	3	63.03	203.145	3	0.4%	0.16 [-1.45, 1.77]
ACE+LID+BUV (Durst, M. S. 2021) MGS	0.58	5.8	3	0.59	6.3	3	0.4%	-0.00 [-1.60, 1.60]
ACE+LID+BUV (Durst, M. S. 2021) NEST	0.28	1.39	3	0.77	2.15	3	0.4%	-0.22 [-1.83, 1.40]
BUP0.1 (Cho, C. et al. 2019) MGS	0.18	1.44	8	0.42	3.36	8	0.6%	-0.09 [-1.07, 0.89]
BUP0.1 (Cho, C. et al. 2019) MGS	0.07	0.56	8	0.28	2.24	8	0.6%	-0.12 [-1.10, 0.86]
BUP0.2 (Clark, M. D. et al. 2004) RW	46.71	32.82	10	34.6	10.6	6	0.6%	0.42 [-0.60, 1.45]
BUP0.2 (Clark, M. D. et al. 2004) VS	46.71	32.82	10	34.6	10.6	6	0.6%	0.42 [-0.60, 1.45]
CAR10 (Cho, C. et al. 2019) MGS	0.18	1.44	8	0.42	3.36	8	0.6%	-0.09 [-1.07, 0.89]
CAR10 (Cho, C. et al. 2019) MGS	0.05	0.4	8	0.42	3.36	8	0.6%	-0.15 [-1.13, 0.84]
CAR25 (Cho, C. et al. 2019) MGS	0.17	1.36	8	0.28	2.24	8	0.6%	-0.06 [-1.04, 0.92]
CAR25 (Cho, C. et al. 2019) MGS	0.09	0.72	8	0.28	2.24	8	0.6%	-0.11 [-1.09, 0.87]
INDO1 (Brusco, I. et al. 2017) VF	3.23	0.79	6	1.44	1.27	6	0.5%	1.56 [0.20, 2.93]
INDO3 (Brusco, I. et al. 2017) VF	4.06	0.48	6	1.44	1.27	6	0.4%	2.52 [0.85, 4.19]
INDO10 (Brusco, I. et al. 2017) SP	5.5	21.89	6	8.94	6.89	6	0.6%	-0.20 [-1.33, 0.94]
INDO10 (Brusco, I. et al. 2017) SP	0.93	12.46	6	1.98	0.49	6	0.6%	-0.11 [-1.24, 1.02]
INDO10 (Brusco, I. et al. 2017) VF	4.23	0.59	6	1.44	1.27	6	0.4%	2.60 [0.90, 4.30]
INDO10 (Brusco, I. et al. 2017) VF	4.3	0.48	6	1.77	1.07	6	0.4%	2.82 [1.04, 4.60]
MEL2(Cho, C. et al. 2019) MGS	0.11	0.88	8	0.42	3.36	8	0.6%	-0.12 [-1.10, 0.86]
MEL2 (Cho, C. et al. 2019) MGS	0.2	1.6	8	0.42	3.36	8	0.6%	-0.08 [-1.06, 0.90]
MEL5 (Cho, C. et al. 2019) MGS	0.08	0.64	8	0.28	2.24	8	0.6%	-0.11 [-1.10, 0.87]
MEL5 (Cho, C. et al. 2019) MGS	0.06	0.48	8	0.28	2.24	8	0.6%	-0.13 [-1.11, 0.85]
MOR1 (Clark, J. D. et al. 2007) VF	2.47	0.72	5	0.22	0.25	5	0.2%	3.77 [1.31, 6.23]
MOR10 (Clark, J. D. et al. 2007) VF	0.66	0.18	5	0.22	0.25	5	0.4%	1.82 [0.21, 3.43]
MOR1 (Dallazen, J. L et al. 2022) HT	12.27	3.74	6	4.48	1.35	6	0.4%	2.56 [0.87, 4.24]
MOR1 (Dallazen, J. L et al. 2022) SP	6.01	16.9	6	6.01	16.04	6	0.6%	0.00 [-1.13, 1.13]
MOR1 (Dallazen, J. L et al. 2022) VF	0.97	0.28	6	0.04	0.16	6	0.3%	3.86 [2.03, 5.68]
OXYM-LE 2 (Clark, M. D. et al. 2004) VS	104.67	27.35	10	34.6	10.6	6	0.4%	2.90 [1.36, 4.44]
LID+BUV (Durst, M. S. 2021) BR	103.03	203.145	3	63.03	203.145	3	0.4%	0.16 [-1.45, 1.77]
LID+BUV (Durst, M. S. 2021) MGS	0.41	4.1	3	0.58	6.3	3	0.4%	-0.03 [-1.63, 1.57]
BUV5 (Leach, M. C. et al. 2012) MGS	1.99	11.94	6	3.86	23.16	6	0.6%	-0.09 [-1.23, 1.04]
BUP+MEL+BUV (Furumoto, K. 2021) NEST	1.3	0.47	3	3.17	1.61	3	0.3%	-1.26 [-3.27, 0.75]
LID+BUV (Durst, M. S. 2021) NEST	0.89	2.18	3	0.77	2.15	3	0.4%	0.04 [-1.56, 1.65]
ACE60 (Zhu, Q. et al. 2016) VF	28.32	37.41	10	100	346.41	10	0.6%	-0.28 [-1.16, 0.60]
ACE30 (Zhu, Q. et al. 2016) VF	21.6	21.23	10	100	346.41	10	0.6%	-0.31 [-1.19, 0.58]
BUP0.05 (Wright-Williams, S. L. 2013) VS	2	2.71	8	4.97	2.26	8	0.6%	-1.13 [-2.20, -0.05]
ACE120 (Zhu, Q. et al. 2016) VF	33.28	18.22	10	100	346.41	10	0.6%	-0.26 [-1.14, 0.62]
ACE240 (Zhu, Q. et al. 2016) VF	43.21	33.35	10	100	346.41	10	0.6%	-0.22 [-1.10, 0.66]
ACE360 (Zhu, Q. et al. 2016) VF	60.14	29.341	10	100	346.41	10	0.6%	-0.16 [-1.03, 0.72]
DIC0.25 (Lu, F. et al. 2022) VF	3.84	0.69	11	3.99	0.12	11	0.7%	-0.29 [-1.13, 0.55]
BUP-SR1 (Navarro K, et al. 2020) HT	6.45	3.76	12	3.22	5.29	8	0.6%	0.70 [-0.23, 1.63]
DIC0.25 (Lu, F. et al. 2022) VF	1.29	0.79	11	1.57	1.55	11	0.7%	-0.22 [-1.06, 0.62]
KET50 (Minville, V. et al. 2008) HT	11.91	1.24	10	5.95	2.88	10	0.5%	2.57 [1.33, 3.82]
KET50 (Minville, V. et al. 2008) VF	3.99	7.33	10	1.03	1.82	10	0.6%	0.53 [-0.36, 1.43]
CEL1 (Muratani T. et al. 2005) VF	5.65	2.34	6	5.89	3.24	6	0.6%	-0.08 [-1.21, 1.05]
CEL1 (Muratani T. et al. 2005) VF	5.96	1.62	6	5.89	3.24	6	0.6%	0.03 [-1.11, 1.16]
CEL10 (Muratani T. et al. 2005) VF	6.2	3	6	8.58	3.9	6	0.6%	-0.63 [-1.80, 0.54]
CEL10 (Muratani T. et al. 2005) VF	6.5	2.31	6	8.58	3.9	6	0.6%	-0.60 [-1.77, 0.57]
BUP-SR1 (Navarro K, et al. 2020) VF	5.35	2	12	6.26	3.07	8	0.6%	-0.35 [-1.26, 0.55]
BUP-XR3.25 (Navarro K, et al. 2020) HT	9.07	6.72	12	3.22	5.29	8	0.6%	0.90 [-0.04, 1.85]
BUP0.01 (Wright-Williams, S. L. 2013) VS	1	0.79	8	4.97	2.26	8	0.5%	-2.22 [-3.54, -0.90]
BUP-XR3.25 (Navarro K, et al. 2020) VF	5.45	2.84	12	6.26	3.07	8	0.6%	-0.26 [-1.16, 0.63]
DEX100 (Romero-Alejo, E. et al. 2016) VF	0.9	0.1	10	0.36	0.18	10	0.4%	3.55 [2.04, 5.06]
BUP-XR6.25 (Navarro K, et al. 2020) HT	8.47	3.76	12	3.22	5.29	8	0.6%	1.14 [0.16, 2.12]
BUP-XR6.25 (Navarro K, et al. 2020) VF	6.29	2.28	12	6.26	3.07	8	0.6%	0.01 [-0.88, 0.91]
GABA20 (Yang J. et al. 2019) VF	0.28	0.14	10	0.051	0.02	9	0.5%	2.13 [0.95, 3.31]
GABA50 (Yang J. et al. 2019) VF	0.53	0.08	6	0.051	0.02	5	0.1%	7.17 [3.25, 11.09]
IBU100 (Saad, S. S. T. et al. 2016) VF	2.58	0.96	9	0.74	0.45	8	0.4%	2.28 [0.99, 3.57]
IBU300 (Saad, S. S. T. et al. 2016) VF	2.98	2.28	9	0.74	0.45	8	0.6%	1.26 [0.19, 2.32]
MEL5 (Roughan, V. J. et al. 2009) VS	16.95	14.11	8	10.97	27.26	8	0.6%	0.26 [-0.72, 1.25]
MEL10 (Roughan, V. J. et al. 2009) VS	24.27	26.33	8	10.97	27.26	8	0.6%	0.47 [-0.53, 1.47]
MEL10 (Roughan, V. J. et al. 2016) VS	6.37	4.48	8	3.76	5.13	9	0.6%	0.51 [-0.46, 1.48]
MEL20 (Roughan, V. J. et al. 2009) VS	50.54	20.7	8	10.97	27.26	8	0.6%	1.55 [0.39, 2.70]
MEL20 (Wright-Williams, S. L. 2009) VS	8.5	9.8	8	17.1	30.8	8	0.6%	-0.36 [-1.35, 0.63]
MEL20 (Leach, M. C. et al. 2012) MGS	2.35	14.1	6	3.86	23.16	6	0.6%	-0.07 [-1.20, 1.06]
MOR3 (Minville, V. et al. 2008) HT	11.96	1.05	10	5.95	2.8	10	0.4%	2.72 [1.44, 4.01]
MOR3 (Minville, V. et al. 2008) VF	3.98	6.64	10	1.03	1.82	10	0.6%	0.58 [-0.32, 1.48]
MOR3 (Pogatzki, E. M & Raja, S. N. 2003) HT	6.33	3.41	15	1.89	1.13	9	0.6%	1.53 [0.58, 2.48]
MOR3* (Wei, S. et al. 2021) VF	2.79	0.44	8	0.32	0.34	8	0.2%	5.94 [3.37, 8.50]
MOR5* (Wei, S. et al. 2021) VF	3.49	1.51	8	0.32	0.34	8	0.4%	2.74 [1.27, 4.21]
MOR10* (Wei, S. et al. 2021) VF	5.52	0.94	8	0.32	0.34	8	0.2%	6.96 [4.01, 9.90]
MOR10 (Minville, V. et al. 2008) HT	11.95	0.87	10	5.95	2.88	10	0.4%	2.70 [1.42, 3.98]
MOR10 (Minville, V. et al. 2008) VF	8	0.91	10	1.03	1.82	10	0.4%	4.64 [2.81, 6.47]
MOR10 (Pogatzki, E. M & Raja, S. N. 2003) VF	7.81	0.01	15	2.16	7.35	15	0.7%	1.06 [0.29, 1.83]
MOR10 (Pogatzki, E. M & Raja, S. N. 2003) HT	13.27	7.82	15	1.89	1.13	9	0.6%	1.75 [0.76, 2.74]
OXY3 (Mueller, A. et al. 2019) VF	1.43	0.24	6	1.19	0.12	5	0.5%	1.12 [-0.20, 2.44]
PRE30 (Park, J. H et al. 2020) VF	6.5	4	8	8.59	2.12	8	0.6%	-0.62 [-1.63, 0.39]
ROP5% (Yamada, T. et al. 2016) HT	20.4	2.885	9	9.77	3.6	9	0.5%	3.10 [1.63, 4.58]
ROP5% (Yamada, T. et al. 2016) HT	18.11	7.219	9	10.54	3.22	9	0.6%	1.29 [0.25, 2.33]
ROP5% (Yamada, T. et al. 2016) VF	1.97	0.42	9	0.16	1.14	9	0.6%	2.01 [0.82, 3.19]
ROP5% (Yamada, T. et al. 2016) VF	1.88	1.95	9	0.1	0.76	9	0.6%	1.15 [0.13, 2.16]
TRA100 (Romero-Alejo, E. et al. 2016) VF	1.14	0.14	10	0.36	0.18	10	0.4%	4.63 [2.81, 6.46]
TRA+DEXK (Romero-Alejo, E. 2016) VF	0.82	0.14	3	0.36	0.18	3	0.2%	2.28 [-0.44, 5.01]
**Subtotal (95% CI)**			**810**			**758**	**51.2%**	**0.84 [0.60, 1.08]**
Heterogeneity: Tau² = 1.05; Chi² = 411.55, df = 95 (P < 0.00001); I² = 77%								
Test for overall effect: Z = 6.85 (P < 0.00001)								
**1.2.2 Female**								
ACE+LID+BUV (Durst, M. S. 2021) BR	101.2	153.35	3	68.67	156.75	3	0.4%	0.17 [-1.44, 1.78]
ACE+LID+BUV (Durst, M. S. 2021) MGS	0.61	4.88	3	0.75	4.4	3	0.4%	-0.02 [-1.62, 1.58]
ACE+LID+BUV (Durst, M. S. 2021) NEST	0.7	1.27	3	1.11	1.38	3	0.4%	-0.25 [-1.87, 1.37]
ACE200 (Durst, M. S. et al. 2021) BR	90.36	180.59	8	68.67	156.75	8	0.6%	0.12 [-0.86, 1.10]
ACE200 (Durst, M. S. et al. 2021) MGS	0.54	4.32	8	0.62	4.4	8	0.6%	-0.02 [-1.00, 0.96]
ACE200 (Durst, M. S. et al. 2021) NEST	0.5	1.49	8	1.11	1.38	8	0.6%	-0.40 [-1.39, 0.59]
BUP+CAR (Adamsom, T. W) 2010 RW	0.17	0.18	2	0.15	0.13	2	0.3%	0.07 [-1.93, 2.08]
BUP+CAR (Adamsom, T. W. 2010) VS	1.4	0.55	2	1.15	0.37	2	0.2%	0.30 [-2.31, 2.92]
BUP0.009 (Jirkof, P. et al. 2019) MGS	4.8	19.2	8	2.06	19.04	4	0.5%	0.13 [-1.07, 1.33]
BUP0.009 (Jirkof, P. et al. 2019) MGS	3.85	15.4	8	1.28	12.4	4	0.5%	0.16 [-1.04, 1.37]
BUP0.1 (Cho, C. et al. 2019) MGS	0.13	1.04	8	0.38	3.04	8	0.6%	-0.10 [-1.08, 0.88]
BUP0.1 (Cho, C. et al. 2019) MGS	0.04	0.32	8	0.25	2	8	0.6%	-0.14 [-1.12, 0.84]
BUP0.1 (Jirkof, P. et al. 2015) BR	4.08	2.57	8	10.3	26.3	8	0.6%	-0.31 [-1.30, 0.67]
BUP0.1 (Jirkof, P. et al. 2015) VS	1.32	1.95	8	0.88	0.84	8	0.6%	0.28 [-0.71, 1.26]
BUP0.1-0.15(MolinaCimadevila M. J. et al. 2014) HT	35.2	36.24	6	63	199	6	0.6%	-0.18 [-1.31, 0.96]
BUP0.1-0.3(MolinaCimadevila M. J. et al. 2014) HT	13.76	29.74	6	63	199	6	0.6%	-0.32 [-1.46, 0.82]
BUP0.1 HCl (Kendal, L. V. et al. 2016) NEST	94.1	8.6	7	62.8	46.8	7	0.6%	0.87 [-0.24, 1.99]
BUP0.1 HCl (Kendal, L. V. et al. 2016) RW	9.5	16.1	6	43.2	33.2	7	0.5%	-1.17 [-2.39, 0.05]
BUP0.1 HCl (Kendal, L. V. et al. 2016) VS	1.7	0.3	6	1	0.7	7	0.5%	1.17 [-0.04, 2.39]
BUP0.2 (Adamson, T. W. et al. 2010) RW	0.5	0.87	7	0.15	0.13	7	0.6%	0.53 [-0.55, 1.60]
BUP0.2 (Adamson, T. W. et al. 2010) VS	1.71	1.08	7	1.15	0.37	7	0.6%	0.65 [-0.44, 1.73]
BUP0.2 (Reddyjarugu, B. et al. 2015) HT	4.76	4.34	10	2.97	1.66	9	0.6%	0.51 [-0.41, 1.43]
BUP0.2 (Reddyjarugu, B. et al. 2015) VF	2.83	0.91	10	1.1	3.11	9	0.6%	0.74 [-0.20, 1.68]
BUP0.6 (Kendal, L. V. et al. 2016) NEST	69.3	41.5	7	62.9	46.8	7	0.6%	0.14 [-0.91, 1.18]
BUP0.6 (Kendal, L. V. et al. 2016) RW	21.1	26.1	7	43.2	33.2	7	0.6%	-0.69 [-1.78, 0.40]
BUP0.6 (Kendal, L. V. et al. 2016) VS	1.25	0.5	7	1	0.7	7	0.6%	0.38 [-0.68, 1.45]
BUP2 (Goecke, J. C. et al. 2005) VS	0.59	0.15	7	0.18	0.09	6	0.4%	3.02 [1.25, 4.79]
BUP2 (Goecke, J. C. et al. 2005) VS	0.38	0.26	6	0.18	0.09	6	0.5%	0.95 [-0.27, 2.17]
BUP-SR2.2 (Jirkof, P. et al. 2015) BR	3.02	1.32	8	10.3	26.3	8	0.6%	-0.37 [-1.36, 0.62]
BUP-SR2.2 (Jirkof, P. et al. 2015) VS	0.37	0.76	8	0.88	0.84	8	0.6%	-0.60 [-1.61, 0.41]
CAR10 (Cho, C. et al. 2019) MGS	0.24	1.92	8	0.38	3.04	8	0.6%	-0.05 [-1.03, 0.93]
CAR10 (Cho, C. et al. 2019) MGS	0.11	0.88	8	0.38	3.04	8	0.6%	-0.11 [-1.10, 0.87]
CAR25 (Cho, C. et al. 2019) MGS	0.01	0.1	8	0.25	2	8	0.6%	-0.16 [-1.14, 0.82]
CAR25 (Cho, C. et al. 2019) MGS	0.06	0.48	8	0.25	2	8	0.6%	-0.12 [-1.10, 0.86]
CAR5 (Adamson, T. W. et al. 2010) RW	0.31	0.26	7	0.15	0.13	7	0.6%	0.73 [-0.37, 1.82]
CAR5 (Adamson, T. W. et al. 2010) VS	0.96	0.47	7	1.15	0.37	7	0.6%	-0.42 [-1.48, 0.64]
CAR5 (Jirkof, P. et al. 2012) VS	248.38	110.33	8	191.93	136.44	8	0.6%	0.43 [-0.56, 1.43]
FIR10 (Reddyjarugu, B. et al. 2015) HT	5.59	4.6	9	2.97	1.66	9	0.6%	0.72 [-0.24, 1.68]
FIR10 (Reddyjarugu, B. et al. 2015) VF	2.41	1.38	9	1.1	3.11	9	0.6%	0.52 [-0.43, 1.46]
FIR20 (Reddyjarugu, B. et al. 2015) HT	7.14	3.9	10	2.97	1.66	9	0.6%	1.30 [0.29, 2.32]
FIR20 (Reddyjarugu, B. et al. 2015) VF	2.24	0.47	10	1.1	3.11	9	0.6%	0.50 [-0.41, 1.42]
FLU2.5 (Goecke, J. C. et al. 2005) VS	0.23	0.13	7	0.18	0.09	6	0.6%	0.41 [-0.70, 1.52]
FLU2.5 (Goecke, J. C. et al. 2005) VS	0.27	0.14	6	0.18	0.09	6	0.6%	0.71 [-0.48, 1.89]
LID+BUV (Durst, M. S. 2021) BR	26.5	93.43	3	68.67	156.75	3	0.4%	-0.26 [-1.88, 1.36]
LID+BUV (Durst, M. S. 2021) MGS	0.26	2.08	3	0.35	4.4	3	0.4%	-0.02 [-1.62, 1.58]
LID+BUV (Durst, M. S. 2021) NEST	0.4	1.41	3	1.11	1.38	3	0.4%	-0.41 [-2.06, 1.24]
MEL+BUP (Molina-Cimadevila, M. J. 2014) HT	35.36	37.48	2	63	199	2	0.3%	-0.11 [-2.17, 1.95]
MEL2(Cho, C. et al. 2019) MGS	0.14	1.12	8	0.38	3.04	8	0.6%	-0.10 [-1.08, 0.88]
MEL2 (Cho, C. et al. 2019) MGS	0.08	0.64	8	0.38	3.04	8	0.6%	-0.13 [-1.11, 0.85]
MEL5 (Cho, C. et al. 2019) MGS	0.005	0.04	8	0.25	2	8	0.6%	-0.16 [-1.15, 0.82]
MEL5 (Cho, C. et al. 2019) MGS	0.12	0.96	8	0.25	2	8	0.6%	-0.08 [-1.06, 0.90]
MOR10 (Tonello, R. et al. 2020) SP	0.14	0.29	6	7.63	2.93	6	0.3%	-3.32 [-5.30, -1.34]
MOR10 (Tonello, R. et al. 2020) VF	7.99	0.48	6	2.7	0.9	6	0.1%	6.77 [3.28, 10.26]
MOR3 (Tonello, R. et al. 2020) SP	2.62	5.68	6	7.63	2.93	6	0.6%	-1.02 [-2.26, 0.21]
MOR3 (Tonello, R. et al. 2020) VF	6.05	1.29	6	2.7	0.9	6	0.4%	2.78 [1.01, 4.55]
TRA+ACE (Jirkof, P. 2018) MGS	2.3	18.4	8	3.1	24.8	8	0.6%	-0.03 [-1.01, 0.95]
TRA+ACE (Jirkof, P. 2018) NEST	2.97	1.32	8	2.57	0.84	8	0.6%	0.34 [-0.65, 1.33]
TRA+ACE (Jirkof, P. 2018) VS	3.36	2.17	8	4.54	2	8	0.6%	-0.53 [-1.54, 0.47]
TRA+ACE (Jirkof, P. 2018) VS	2.35	1.89	8	3.86	3.1	8	0.6%	-0.56 [-1.56, 0.45]
TRA0.1 (Jirkof, P. et al. 2019) MGS	3.2	12.8	8	3.49	15.2	4	0.5%	-0.02 [-1.22, 1.18]
TRA0.1 (Jirkof, P. et al. 2019) MGS	3.66	14.64	8	3.22	10.08	4	0.5%	0.03 [-1.17, 1.23]
TRA1 (Jirkof, P. et al. 2019) MGS	2.93	11.72	8	2.21	14.72	4	0.5%	0.05 [-1.15, 1.25]
TRA1 (Jirkof, P. et al. 2019) MGS	3	12	8	3.18	19.36	4	0.5%	-0.01 [-1.21, 1.19]
TRA25 (Evangelista-Vaz, R. et al. 2018) BR	2.13	3.42	8	3.64	6.05	8	0.6%	-0.29 [-1.28, 0.70]
TRA25 (Evangelista-Vaz, R. et al. 2018) VS	214	383.81	8	191	156.41	8	0.6%	0.07 [-0.91, 1.05]
TRA80 (Wolfe, A. M. et al. 2015) VS	0.7	0.66	10	1.65	0.79	10	0.6%	-1.25 [-2.23, -0.27]
BUP 0.024 (Faller, K.M.E. 2015)	3.17	1.48	13	1.39	0.58	13	0.6%	1.53 [0.64, 2.43]
**Subtotal (95% CI)**			**475**			**447**	**36.2%**	**0.11 [-0.06, 0.29]**
Heterogeneity: Tau² = 0.20; Chi² = 107.22, df = 66 (P = 0.001); I² = 38%								
Test for overall effect: Z = 1.28 (P = 0.20)								
**1.2.3 Both sexes**								
BUP0.05 (Matsumya L. C, et al. 2012) MGS	0.18	2.88	16	0.48	7.68	4	0.6%	-0.07 [-1.17, 1.03]
BUP0.1 (Matsumya L. C, et al. 2012) MGS	0.11	1.76	16	0.48	7.68	4	0.6%	-0.10 [-1.20, 1.00]
BUP0.1(Oliver V; Thurston S; Logfren J. 2018) VF	4.31	1.351	8	3.05	1.38	8	0.6%	0.87 [-0.17, 1.91]
CAR5 (Jirkof, P. et al. 2010) BR	35	41	7	10.41	5.06	8	0.6%	0.82 [-0.25, 1.89]
CAR5 (Jirkof, P. et al. 2013) NEST	0.04	0.22	8	0.1	0.002	8	0.6%	-0.36 [-1.36, 0.63]
CAR20 (Matsumya L. C, et al. 2012) MGS	0.07	1.12	16	0.48	7.68	4	0.6%	-0.12 [-1.22, 0.98]
CAR25 (Matsumya L. C, et al. 2012) MGS	0.15	2.4	16	0.48	7.68	4	0.6%	-0.08 [-1.18, 1.01]
CAR30 (Oliver V; Thurston S; Logfren J. 2018) VF	3.9	1.62	8	3.05	1.38	8	0.6%	0.53 [-0.47, 1.53]
CAR50 (Jirkof, P. et al. 2013) NEST	0.68	1.27	8	0.1	0.002	8	0.6%	0.61 [-0.40, 1.62]
KET20 (Matsumya L. C, et al. 2012) MGS	0.19	3.04	16	0.48	7.68	4	0.6%	-0.07 [-1.16, 1.03]
BUP0.001 (Matsumya L. C, et al. 2012) MGS	0.37	0.68	16	0.48	7.68	4	0.6%	-0.03 [-1.13, 1.06]
BUP0.01 (Matsumya L. C, et al. 2012) MGS	0.22	0.44	16	0.48	7.68	4	0.6%	-0.08 [-1.17, 1.02]
CAR 10 (Matsumya L. C, et al. 2012) MGS	0.52	0.32	16	0.48	7.68	4	0.6%	0.01 [-1.08, 1.11]
CAR 15 (Matsumya L. C, et al. 2012) MGS	0.55	0.36	16	0.48	7.68	4	0.6%	0.02 [-1.07, 1.12]
CAR 5 (Matsumya L. C, et al. 2012) MGS	0.43	0.52	16	0.48	7.68	4	0.6%	-0.02 [-1.11, 1.08]
KETO 1 (Matsumya L. C, et al. 2012) MGS	0.53	0.56	16	0.48	7.68	4	0.6%	0.02 [-1.08, 1.11]
KETO 10 (Matsumya L. C, et al. 2012) MGS	0.64	0.6	16	0.48	7.68	4	0.6%	0.05 [-1.05, 1.14]
KETO 15 (Matsumya L. C, et al. 2012) MGS	0.18	0.44	16	0.48	7.68	4	0.6%	-0.09 [-1.19, 1.01]
KETO 5 (Matsumya L. C, et al. 2012) MGS	0.43	0.2	16	0.48	7.68	4	0.6%	-0.02 [-1.11, 1.08]
ACE 100 (Matsumya L. C, et al. 2012) MGS	0.56	0.57	16	0.48	7.68	4	0.6%	0.02 [-1.07, 1.12]
ACE 300 (Matsumya L. C, et al. 2012) MGS	0.37	0.72	16	0.48	7.68	4	0.6%	-0.03 [-1.13, 1.06]
ACE 450 (Matsumya L. C, et al. 2012) MGS	0.32	0.68	16	0.48	7.68	4	0.6%	-0.05 [-1.14, 1.05]
**Subtotal (95% CI)**			**311**			**108**	**12.6%**	**0.09 [-0.14, 0.32]**
Heterogeneity: Tau² = 0.00; Chi² = 7.57, df = 21 (P = 1.00); I² = 0%								
Test for overall effect: Z = 0.79 (P = 0.43)								
**Total (95% CI)**			**1596**			**1313**	**100.0%**	**0.46 [0.32, 0.60]**
Heterogeneity: Tau² = 0.64; Chi² = 559.38, df = 184 (P < 0.00001); I² = 67%								
Test for overall effect: Z = 6.24 (P < 0.00001)								
Test for subgroup differences: Chi² = 26.75, df = 2 (P < 0.00001), I² = 92.5%								

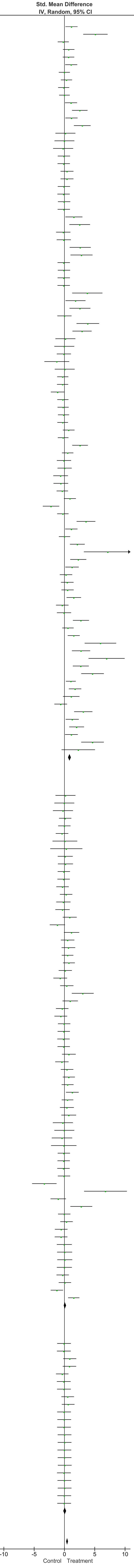

Supplementary figure 2. Subgroup analysis based on sex considering all analgesic treatments in all pain modalities. Forest plot showing pooled SMDs (95% CI) for studies grouped by sex category (female, male, and mixed/unspecified). The analysis includes all analgesic treatments across all pain modalities.

Study or Subgroup	Treatment Mean	Treatment SD	Treatment Total	Control Mean	Control SD	Control Total	Weight	Std. Mean Difference IV, Random, 95% CI
**1.4.1 Mild**								
ACE200 (Cabañero & Maldonado. 2021) VF	0.89	0.24	10	0.38	0.13	10	0.5%	2.53 [1.29, 3.77]
ACE30 (Zhu, Q. et al. 2016) VF	21.6	21.23	10	100	346.41	10	0.6%	-0.31 [-1.19, 0.58]
ACE50 (Cabañero & Maldonado. 2021) HT	3.2	2	10	3.58	1.69	10	0.6%	-0.20 [-1.08, 0.68]
ACE50 (Cabañero & Maldonado. 2021) VF	0.52	0.24	10	0.38	0.13	10	0.6%	0.69 [-0.21, 1.60]
ACE60 (Zhu, Q. et al. 2016) VF	28.32	37.41	10	100	346.41	10	0.6%	-0.28 [-1.16, 0.60]
ACE100 (Cabañero & Maldonado. 2021) HT	4.65	1.35	10	3.58	1.69	10	0.6%	0.67 [-0.24, 1.58]
ACE100 (Cabañero & Maldonado. 2021) VF	0.58	0.2	10	0.38	0.13	10	0.6%	1.14 [0.18, 2.10]
ACE120 (Zhu, Q. et al. 2016) VF	33.28	18.22	10	100	346.41	10	0.6%	-0.26 [-1.14, 0.62]
ACE200 (Cabañero & Maldonado. 2021) HT	6.3	2.94	10	3.58	1.69	10	0.6%	1.09 [0.13, 2.04]
ACE240 (Zhu, Q. et al. 2016) VF	43.21	33.35	10	100	346.41	10	0.6%	-0.22 [-1.10, 0.66]
ACE300 (Cabañero & Maldonado. 2021) HT	6.02	2.28	10	3.58	1.69	10	0.6%	1.16 [0.20, 2.13]
ACE300 (Cabañero & Maldonado. 2021) VF	1.03	0.27	10	0.38	0.13	10	0.5%	2.94 [1.60, 4.28]
ACE360 (Zhu, Q. et al. 2016) VF	60.14	29.34	10	100	346.41	10	0.6%	-0.16 [-1.03, 0.72]
ACE400 (Cabañero & Maldonado. 2021) HT	6.49	2.94	10	3.58	1.69	10	0.6%	1.16 [0.20, 2.13]
ACE400 (Cabañero & Maldonado. 2021) VF	1.28	0.2	10	0.38	0.13	10	0.3%	5.11 [3.14, 7.08]
BUP0.1 (Cho, C. et al. 2019) MGS	0.18	1.44	8	0.42	3.36	8	0.6%	-0.09 [-1.07, 0.89]
BUP0.1 (Cho, C. et al. 2019) MGS	0.13	1.04	8	0.38	3.04	8	0.6%	-0.10 [-1.08, 0.88]
BUP0.1 (Cho, C. et al. 2019) MGS	0.07	0.56	8	0.28	2.24	8	0.6%	-0.12 [-1.10, 0.86]
BUP0.1 (Cho C. et al. 2019) MGS	0.04	0.32	8	0.25	2	8	0.6%	-0.14 [-1.12, 0.84]
BUP0.2 (Adamson, T. W. et al. 2010) RW	0.5	0.87	7	0.15	0.13	7	0.6%	0.53 [-0.55, 1.60]
BUP0.2 (Adamson, T. W. et al. 2010) VS	1.71	1.08	7	1.15	0.37	7	0.6%	0.65 [-0.44, 1.73]
BUP1.67x10³(Lu, F. et al. 2022) VF	3.99	0.08	11	3.99	0.12	11	0.7%	0.00 [-0.84, 0.84]
BUP+CAR (Adamsom, T. W. 2010) RW	0.17	0.18	2	0.15	0.13	2	0.3%	0.07 [-1.93, 2.08]
BUP+CAR (Adamsom, T. W. 2010) VS	1.4	0.55	2	1.15	0.37	2	0.2%	0.30 [-2.31, 2.92]
BUV5 (Leach, M. C. et al. 2012) MGS	1.99	11.94	6	3.86	23.16	6	0.6%	-0.09 [-1.23, 1.04]
CAR5 (Adamson, T. W. et al. 2010) VS	0.96	0.47	7	1.15	0.37	7	0.6%	-0.42 [-1.48, 0.64]
CAR5 (Adamson, T. W. et al. 2010) RW	0.31	0.26	7	0.15	0.13	7	0.6%	0.73 [-0.37, 1.82]
CAR10 (Cho, C. et al. 2019) MGS	0.18	1.44	8	0.42	3.36	8	0.6%	-0.09 [-1.07, 0.89]
CAR10 (Cho, C. et al. 2019) MGS	0.24	1.92	8	0.38	3.04	8	0.6%	-0.05 [-1.03, 0.93]
CAR10 (Cho, C. et al. 2019) MGS	0.17	1.36	8	0.28	2.24	8	0.6%	-0.06 [-1.04, 0.92]
CAR20 (Matsumya L. C, et al. 2012) MGS	0.07	1.12	16	0.48	7.68	4	0.6%	-0.12 [-1.22, 0.98]
CAR10 (Cho, C. et al. 2019) MGS	0.01	0.01	8	0.25	2	8	0.6%	-0.16 [-1.14, 0.82]
CAR25 (Cho, C. et al. 2019) MGS	0.11	0.88	8	0.38	3.04	8	0.6%	-0.11 [-1.10, 0.87]
CAR25 (Cho, C. et al. 2019) MGS	0.05	0.4	8	0.42	3.36	8	0.6%	-0.15 [-1.13, 0.84]
CAR25 (Cho, C. et al. 2019) MGS	0.06	0.48	8	0.25	2	8	0.6%	-0.12 [-1.10, 0.86]
CAR25 (Cho, C. et al. 2019) MGS	0.09	0.72	8	0.28	2.24	8	0.6%	-0.11 [-1.09, 0.87]
DIC0.25 (Lu, F. et al. 2022) VF	1.29	0.79	11	1.57	1.55	11	0.7%	-0.22 [-1.06, 0.62]
DIC0.25 (Lu, F. et al. 2022) VF	3.84	0.69	11	3.99	0.12	11	0.7%	-0.29 [-1.13, 0.55]
CAR25 (Matsumya L. C, et al. 2012) MGS	0.15	2.4	16	0.48	7.68	4	0.6%	-0.08 [-1.18, 1.01]
BUP0.05 (Matsumya L. C, et al. 2012) MGS	0.18	2.88	16	0.48	7.68	4	0.6%	-0.07 [-1.17, 1.03]
BUP0.1 (Matsumya L. C, et al. 2012) MGS	0.11	1.76	16	0.48	7.68	4	0.6%	-0.10 [-1.20, 1.00]
CEL1 (Muratani T. et al. 2005) VF	5.96	1.62	6	5.89	3.24	6	0.6%	0.03 [-1.11, 1.16]
CEL1 (Muratani T. et al. 2005) VF	5.65	2.34	6	5.89	3.24	6	0.6%	-0.08 [-1.21, 1.05]
INDO1 (Brusco, I. et al. 2017) VF	3.23	0.79	6	1.44	1.27	6	0.5%	1.56 [0.20, 2.93]
INDO3 (Brusco, I. et al. 2017) VF	4.06	0.48	6	1.44	1.27	6	0.4%	2.52 [0.85, 4.19]
INDO10 (Brusco, I. et al. 2017) SP	5.5	21.8	6	8.94	6.89	6	0.6%	-0.20 [-1.33, 0.94]
INDO10 (Brusco, I. et al. 2017) SP	0.93	12.46	6	1.98	0.49	6	0.6%	-0.11 [-1.24, 1.02]
INDO10 (Brusco, I. et al. 2017) VF	4.23	0.59	6	1.44	1.27	6	0.4%	2.60 [0.90, 4.30]
INDO10 (Brusco, I. et al. 2017) VF	4.3	0.48	6	1.77	1.07	6	0.4%	2.82 [1.04, 4.60]
MEL2(Cho, C. et al. 2019) MGS	0.11	0.88	8	0.42	3.36	8	0.6%	-0.12 [-1.10, 0.86]
MEL5 (Cho, C. et al. 2019) MGS	0.2	1.6	8	0.42	3.36	8	0.6%	-0.08 [-1.06, 0.90]
MEL2 (Cho, C. et al. 2019) MGS	0.14	1.12	8	0.38	3.04	8	0.6%	-0.10 [-1.08, 0.88]
MEL5 (Cho, C. et al. 2019) MGS	0.08	0.64	8	0.38	3.04	8	0.6%	-0.13 [-1.11, 0.85]
MEL2 (Cho, C. et al. 2019) MGS	0.08	0.64	8	0.28	2.24	8	0.6%	-0.11 [-1.10, 0.87]
MEL5 (Cho, C. et al. 2019) MGS	0.06	0.48	8	0.28	2.24	8	0.6%	-0.13 [-1.11, 0.85]
MEL2 (Cho, C. et al. 2019) MGS	0.005	0.04	8	0.25	2	8	0.6%	-0.16 [-1.15, 0.82]
MEL5 (Cho, C. et al. 2019) MGS	0.05	0.2	8	0.25	2	8	0.6%	-0.08 [-1.06, 0.90]
MOR1 (Clark, J. D. et al. 2007) VF	2.47	0.72	5	0.22	0.25	5	0.2%	3.77 [1.31, 6.23]
MOR10 (Clark, J. D. et al. 2007) VF	0.66	0.18	5	0.22	0.25	5	0.4%	1.82 [0.21, 3.43]
MOR1 (Dallazen, J. L et al. 2022) VF	0.97	0.28	8	0.04	0.16	8	0.4%	3.86 [2.03, 5.68]
MOR1 (Dallazen, J. L et al. 2022) VF	12.27	3.74	6	4.48	1.35	6	0.4%	2.56 [0.87, 4.24]
MOR1 (Dallazen, J. L et al. 2022) SP	6.01	16.9	6	6.01	16.04	6	0.6%	0.00 [-1.13, 1.13]
MEL20 (Leach, M. C. et al. 2012) MGS	2.35	14.1	6	3.86	23.16	6	0.6%	-0.07 [-1.20, 1.06]
KET20 (Matsumya L. C, et al. 2012) MGS	0.19	3.04	16	0.48	7.68	4	0.6%	-0.07 [-1.16, 1.03]
OXY3 (Mueller, A. et al. 2019) VF	1.43	0.242487	6	1.19	0.126491	5	0.5%	1.10 [-0.22, 2.42]
CEL10 (Muratani T. et al. 2005) VF	6.2	3	6	8.58	3.9	6	0.6%	-0.63 [-1.80, 0.54]
CEL10 (Muratani T. et al. 2005) VF	6.5	2.31	6	8.58	3.9	6	0.6%	-0.60 [-1.77, 0.57]
BUP-SR1 (Navarro K, et al. 2020) HT	6.45	3.76	12	3.22	5.29	8	0.6%	0.70 [-0.23, 1.63]
BUP-XR3.25 (Navarro K, et al. 2020) HT	9.07	6.72	12	3.22	5.29	8	0.6%	0.90 [-0.04, 1.85]
BUP0.2 (Reddyjarugu, B. et al. 2015) HT	4.76	4.34	10	2.97	1.66	9	0.6%	0.51 [-0.41, 1.43]
BUP-XR6.25 (Navarro K, et al. 2020) HT	8.47	3.76	12	3.22	5.29	8	0.6%	1.14 [0.16, 2.12]
BUP-SR1 (Navarro K, et al. 2020) VF	5.35	2	12	6.26	3.07	8	0.6%	-0.35 [-1.26, 0.55]
BUP-XR3.25 (Navarro K, et al. 2020) VF	5.45	2.84	12	6.26	3.07	8	0.6%	-0.26 [-1.16, 0.63]
BUP-XR6.25 (Navarro K, et al. 2020) VF	6.29	2.28	12	6.26	3.07	8	0.6%	0.01 [-0.88, 0.91]
DEX100 (Romero-Alejo, E. et al. 2016) VF	0.9	0.1	10	0.36	0.18	10	0.4%	3.55 [2.04, 5.06]
FIR10 (Reddyjarugu, B. et al. 2015) HT	5.59	4.6	9	2.97	1.66	9	0.6%	0.72 [-0.24, 1.68]
FIR20 (Reddyjarugu, B. et al. 2015) HT	7.14	3.9	10	2.97	1.66	9	0.6%	1.30 [0.29, 2.32]
IBU100 (Saad, S. S. T. et al. 2016) VF	50.54	20.7	8	10.97	27.26	8	0.5%	1.55 [0.39, 2.70]
IBU300 (Saad, S. S. T. et al. 2016) VF	24.27	26.33	8	10.97	27.26	8	0.6%	0.47 [-0.53, 1.47]
PRE30 (Park, J. H et al. 2020) VF	6.5	4	8	8.59	2.12	8	0.6%	-0.62 [-1.63, 0.39]
MOR3 (Pogatzki, E. M. & Raja, S. N. 2003) HT	6.33	3.41	15	1.89	1.13	9	0.6%	1.53 [0.58, 2.48]
MOR10 (Pogatzki, E. M. & Raja, S. N. 2003) HT	13.27	7.82	15	1.89	1.13	9	0.6%	1.75 [0.76, 2.74]
MOR10 (Pogatzki, E. M & Raja, S. N. 2003) VF	7.81	0.01	15	2.16	7.35	15	0.7%	1.06 [0.29, 1.83]
BUP0.2 (Reddyjarugu, B. et al. 2015) VF	2.83	0.91	10	1.1	3.11	9	0.6%	0.74 [-0.20, 1.68]
FIR10 (Reddyjarugu, B. et al. 2015) VF	2.41	1.38	9	1.1	3.11	9	0.6%	0.52 [-0.43, 1.46]
FIR20 (Reddyjarugu, B. et al. 2015) VF	2.24	0.47	10	1.1	3.11	9	0.6%	0.50 [-0.41, 1.42]
MEL5 (Roughan, V. J. et al. 2009) VS	16.95	14.11	8	10.97	27.26	8	0.6%	0.26 [-0.72, 1.25]
MEL10 (Roughan, V. J. et al. 2016) VS	2.58	0.96	9	0.74	0.45	8	0.5%	2.28 [0.99, 3.57]
MEL20 (Roughan, V. J. et al. 2009) VS	2.98	2.28	9	0.74	0.45	8	0.6%	1.26 [0.19, 2.32]
MOR3 (Tonello, R. et al. 2020) SP	2.62	5.68	6	7.63	2.93	6	0.5%	-1.02 [-2.26, 0.21]
MOR10 (Tonello, R. et al. 2020) SP	0.14	0.29	6	7.63	2.93	6	0.3%	-3.32 [-5.30, -1.34]
MOR10 (Tonello, R. et al. 2020) VF	6.05	1.29	6	2.7	0.9	6	0.4%	2.78 [1.01, 4.55]
MOR3 (Tonello, R. et al. 2020) VF	7.99	0.48	6	2.7	0.9	6	0.1%	6.77 [3.28, 10.26]
MOR3* (Wei, S. et al. 2021) VF	2.79	0.44	8	0.32	0.34	8	0.2%	5.94 [3.37, 8.50]
MOR5* (Wei, S. et al. 2021) VF	3.49	1.51	8	0.32	0.34	8	0.4%	2.74 [1.27, 4.21]
MOR10* (Wei, S. et al. 2021) VF	5.52	0.94	8	0.32	0.34	8	0.2%	6.96 [4.01, 9.90]
ROP5% (Yamada, T. et al. 2016) HT	20.4	2.88	9	9.77	3.6	9	0.4%	3.11 [1.63, 4.58]
ROP5% (Yamada, T. et al. 2016) HT	18.11	7.21	9	10.54	3.22	9	0.6%	1.29 [0.25, 2.33]
ROP5% (Yamada, T. et al. 2016) VF	1.97	0.42	9	0.16	1.14	9	0.5%	2.01 [0.82, 3.19]
ROP5% (Yamada, T. et al. 2016) VF	1.88	1.95	9	0.1	0.76	9	0.6%	1.15 [0.13, 2.16]
TRA100 (Romero-Alejo, E. et al. 2016) VF	1.14	0.14	10	0.36	0.18	10	0.4%	4.63 [2.81, 6.46]
TRA+DEXK (Romero-Alejo, E. 2016) VF	0.82	0.14	3	0.36	0.18	3	0.2%	2.28 [-0.44, 5.01]
BUP0.001 (Matsumya L. C, et al. 2012) MGS	0.37	0.68	16	0.48	7.68	4	0.6%	-0.03 [-1.13, 1.06]
BUP0.01 (Matsumya L. C, et al. 2012) MGS	0.22	0.44	16	0.48	7.68	4	0.6%	-0.08 [-1.17, 1.02]
CAR 10 (Matsumya L. C, et al. 2012) MGS	0.52	0.2	16	0.48	7.68	4	0.6%	0.01 [-1.08, 1.11]
CAR 15 (Matsumya L. C, et al. 2012) MGS	0.55	0.36	16	0.48	7.68	4	0.6%	0.02 [-1.07, 1.12]
CAR 5 (Matsumya L. C, et al. 2012) MGS	0.43	0.52	16	0.48	7.68	4	0.6%	-0.02 [-1.11, 1.08]
KETO 1 (Matsumya L. C, et al. 2012) MGS	0.53	0.56	16	0.48	7.68	4	0.6%	0.02 [-1.08, 1.11]
KETO 10 (Matsumya L. C, et al. 2012) MGS	0.64	0.6	16	0.48	7.68	4	0.6%	0.05 [-1.05, 1.14]
KETO 15 (Matsumya L. C, et al. 2012) MGS	0.18	0.44	16	0.48	7.68	4	0.6%	-0.09 [-1.19, 1.01]
KETO 5 (Matsumya L. C, et al. 2012) MGS	0.43	0.2	16	0.48	7.68	4	0.6%	-0.02 [-1.11, 1.08]
ACE 100 (Matsumya L. C, et al. 2012) MGS	0.57	0.56	16	0.48	7.68	4	0.6%	0.03 [-1.07, 1.12]
ACE 300 (Matsumya L. C, et al. 2012) MGS	0.37	0.72	16	0.48	7.68	4	0.6%	-0.03 [-1.13, 1.06]
ACE 450 (Matsumya L. C, et al. 2012) MGS	0.32	0.68	16	0.48	7.68	4	0.6%	-0.05 [-1.14, 1.05]
**Subtotal (95% CI)**			**1087**			**840**	**62.8%**	**0.57 [0.38, 0.75]**
Heterogeneity: Tau² = 0.69; Chi² = 369.60, df = 113 (P < 0.00001); I² = 69%								
Test for overall effect: Z = 5.92 (P < 0.00001)								
**1.4.2 Moderate**								
ACE200 (Durst, M. S. et al. 2021) NEST	0.52	1.54	10	0.77	2.15	10	0.6%	-0.13 [-1.01, 0.75]
ACE200 (Durst, M. S. et al. 2021) NEST	0.5	1.49	8	1.11	1.38	8	0.6%	-0.40 [-1.39, 0.59]
ACE200 (Durst, M. S. et al. 2021) BR	90.36	180.59	8	68.67	0.01	8	0.6%	0.16 [-0.82, 1.14]
ACE200 (Durst, M. S. et al. 2021) BR	126.06	149.48	10	63.03	203.14	10	0.6%	0.34 [-0.55, 1.22]
ACE200 (Durst, M. S. et al. 2021) MGS	0.63	6.3	10	0.73	6.3	10	0.6%	-0.02 [-0.89, 0.86]
ACE200 (Durst, M. S. et al. 2021) MGS	0.54	4.32	8	0.62	4.4	8	0.6%	-0.02 [-1.00, 0.96]
ACE+LID+BUV (Durst, M. S. 2021) NEST	0.28	1.39	3	0.77	2.15	3	0.4%	-0.22 [-1.83, 1.40]
ACE+LID+BUV (Durst, M. S. 2021) NEST	0.7	1.27	3	1.11	1.38	3	0.4%	-0.25 [-1.87, 1.37]
ACE+LID+BUV (Durst, M. S. 2021) BR	101.81	187.83	3	63.03	203.14	3	0.4%	0.16 [-1.45, 1.77]
ACE+LID+BUV (Durst, M. S. 2021) BR	101.2	153.35	3	68.67	156.75	3	0.4%	0.17 [-1.44, 1.78]
ACE+LID+BUV (Durst, M. S. 2021) MGS	0.58	5.8	3	0.59	6.3	3	0.4%	-0.00 [-1.60, 1.60]
ACE+LID+BUV (Durst, M. S. 2021) MGS	0.61	4.88	3	0.75	4.4	3	0.4%	-0.02 [-1.62, 1.58]
BUP0.009 (Jirkof, P. et al. 2019) MGS	3.85	15.4	8	1.28	12.4	4	0.5%	0.16 [-1.04, 1.37]
BUP0.009 (Jirkof, P. et al. 2019) MGS	4.8	19.2	8	2.06	19.04	4	0.5%	0.13 [-1.07, 1.33]
BUP0.01 (Wright-Williams, S. L. 2013) VS	1	0.79	8	4.97	2.26	8	0.5%	-2.22 [-3.54, -0.90]
BUP0.05 (Wright-Williams, S. L. 2013) VS	2	2.71	8	4.97	2.26	8	0.6%	-1.13 [-2.20, -0.05]
BUP0.1(Oliver V; Thurston S; Logfren J. 2018) VF	4.31	1.35	8	3.05	1.38	8	0.6%	0.87 [-0.17, 1.91]
BUP0.1 (Jirkof, P. et al. 2015) BR	3.02	1.32	8	10.3	26.3	8	0.6%	-0.37 [-1.36, 0.62]
BUP0.1 (Jirkof, P. et al. 2015) VS	0.37	0.76	8	0.88	0.84	8	0.6%	-0.60 [-1.61, 0.41]
BUP0.1 HCl (Kendal, L. V. et al. 2016) NEST	9.5	16.1	6	43.2	33.2	7	0.5%	-1.17 [-2.39, 0.05]
BUP0.1 HCl (Kendal, L. V. et al. 2016) RW	94.1	8.6	7	62.8	46.8	7	0.6%	0.87 [-0.24, 1.99]
BUP0.1 HCl (Kendal, L. V. et al. 2016) VS	1.7	0.3	6	1	0.7	7	0.5%	1.17 [-0.04, 2.39]
BUP0.1-0.15(MolinaCimadevila M. J. et al. 2014) HT	35.2	36.24	6	63	199	6	0.6%	-0.18 [-1.31, 0.96]
BUP0.1-0.3(MolinaCimadevila, M. J. et al. 2014) HT	13.76	29.74	6	63	199	6	0.6%	-0.32 [-1.46, 0.82]
BUP0.2 (Clark, M. D. et al. 2004) BW	46.71	32.8	10	34.6	10.6	6	0.6%	0.42 [-0.60, 1.45]
BUP0.2 (Clark, M. D. et al. 2004) VS	46.71	32.8	10	34.6	10.6	6	0.6%	0.42 [-0.60, 1.45]
BUP0.6 (Kendal, L. V. et al. 2016) NEST	21.1	26.1	7	43.2	33.2	7	0.6%	-0.69 [-1.78, 0.40]
BUP0.6 (Kendal, L. V. et al. 2016) RW	1.25	0.5	7	1	0.7	7	0.6%	0.38 [-0.68, 1.45]
BUP0.6 (Kendal, L. V. et al. 2016) VS	69.3	41.5	7	62.9	46.8	7	0.6%	0.14 [-0.91, 1.18]
BUP2 (Goecke, J. C. et al. 2005) VS	0.59	0.15	7	0.18	0.09	6	0.4%	3.02 [1.25, 4.79]
BUP2 (Goecke, J. C. et al. 2005) VS	0.38	0.26	6	0.18	0.09	6	0.5%	0.95 [-0.27, 2.17]
BUP-SR2.2 (Jirkof, P. et al. 2015) BR	4.08	2.57	8	10.3	26.3	8	0.6%	-0.31 [-1.30, 0.67]
BUP-SR2.2 (Jirkof, P. et al. 2015) VS	1.2	1.95	8	0.88	0.84	8	0.6%	0.28 [-0.71, 1.26]
BUP+MEL+BUV (Furumoto, K. 2021) NEST	1.3	0.47	3	3.17	1.61	3	0.3%	-1.26 [-3.27, 0.75]
CAR5 (Jirkof, P. et al. 2010) BR	35	41	7	10.41	5.06	8	0.6%	0.82 [-0.25, 1.89]
CAR5 (Jirkof, P. et al. 2012) VS	248.38	110.33	8	191.93	136.44	8	0.6%	0.43 [-0.56, 1.43]
CAR5 (Jirkof, P. et al. 2013) NEST	0.04	0.22	8	0.1	0.002	8	0.6%	-0.36 [-1.36, 0.63]
CAR30 (Oliver V; Thurston S; Logfren J. 2018) VF	3.9	1.62	8	3.05	1.38	8	0.6%	0.53 [-0.47, 1.54]
CAR50 (Jirkof, P. et al. 2013) NEST	0.68	1.27	8	0.1	0.002	8	0.6%	0.61 [-0.40, 1.62]
FLU2.5 (Goecke, J. C. et al. 2005) VS	0.23	0.13	7	0.18	0.09	6	0.6%	0.41 [-0.70, 1.52]
FLU2.5 (Goecke, J. C. et al. 2005) VS	0.27	0.14	6	0.18	0.09	6	0.6%	0.71 [-0.48, 1.89]
GABA20 (Yang J. et al. 2019) VF	0.28	0.14	10	0.051	0.02	9	0.5%	2.13 [0.95, 3.31]
GABA50 (Yang J. et al. 2019) VF	0.53	0.08	6	0.051	0.02	5	0.1%	7.17 [3.25, 11.09]
KET50 (Minville, V. et al. 2008) VF	11.91	1.2	10	5.95	2.8	10	0.5%	2.65 [1.38, 3.92]
KET50 (Minville, V. et al. 2008) HT	3.99	7.33	10	1.03	1.82	10	0.6%	0.53 [-0.36, 1.43]
LID+BUV (Durst, M. S. 2021) NEST	0.89	2.18	3	0.77	2.15	3	0.4%	0.04 [-1.56, 1.65]
LID+BUV (Durst, M. S. 2021) NEST	0.4	1.41	3	1.11	1.38	3	0.4%	-0.41 [-2.06, 1.24]
LID+BUV (Durst, M. S. 2021) BR	103.03	203.14	3	63.03	203.14	3	0.4%	0.16 [-1.45, 1.77]
LID+BUV (Durst, M. S. 2021) BR	26.5	95.43	3	68.67	156.75	3	0.4%	-0.26 [-1.88, 1.36]
LID+BUV (Durst, M. S. 2021) MGS	0.41	4.1	3	0.58	6.3	3	0.4%	-0.03 [-1.63, 1.57]
LID+BUV (Durst, M. S. 2021) MGS	0.26	2.08	3	0.35	4.4	3	0.4%	-0.02 [-1.62, 1.58]
MOR3 (Minville, V. et al. 2008) HT	11.96	1.05	10	5.95	2.88	10	0.5%	2.66 [1.39, 3.93]
MOR3 (Minville, V. et al. 2008) VF	3.98	6.64	10	1.03	1.82	10	0.6%	0.58 [-0.32, 1.48]
MOR10 (Minville, V. et al. 2008) HT	11.95	0.87	10	5.95	2.88	10	0.5%	2.70 [1.42, 3.98]
MOR10 (Minville, V. et al. 2008) VF	8	0.91	10	1.03	1.82	10	0.4%	4.64 [2.81, 6.47]
MEL10 (Roughan, V. J. et al. 2016) VS	6.37	4.48	8	3.76	5.13	9	0.6%	0.51 [-0.46, 1.48]
MEL20 (Wright-Williams, S. L. 2009) VS	8.5	9.8	8	17.1	30.8	8	0.6%	-0.36 [-1.35, 0.63]
MEL+BUP (Molina-Cimadevila, M. J. 2014) HT	35.36	37.48	2	63	199	2	0.6%	-0.11 [-2.17, 1.95]
OXYM-LE 2 (Clark, M. D. et al. 2004) VS	104.67	27.35	10	34.6	10.6	6	0.4%	2.90 [1.36, 4.44]
TRA0.1 (Jirkof, P. et al. 2019) MGS	2.93	11.72	8	2.21	14.72	4	0.5%	0.05 [-1.15, 1.25]
TRA0.1 (Jirkof, P. et al. 2019) MGS	3.2	12.8	8	3.49	15.2	4	0.5%	-0.02 [-1.22, 1.18]
TRA1 (Jirkof, P. et al. 2019) MGS	3	12	8	3.18	19.36	4	0.5%	-0.01 [-1.21, 1.19]
TRA1 (Jirkof, P. et al. 2019) MGS	3.66	14.64	8	3.22	10.08	4	0.5%	0.03 [-1.17, 1.23]
TRA25 (Evangelista-Vaz, R. et al. 2018) BR	214	383.81	8	191	156.41	8	0.6%	0.07 [-0.91, 1.05]
TRA25 (Evangelista-Vaz, R. et al. 2018) VS	2.13	3.42	8	3.64	6.05	8	0.6%	-0.29 [-1.28, 0.70]
TRA80 (Wolfe, A. M. et al. 2015) VS	0.7	0.66	10	1.65	0.79	10	0.6%	-1.25 [-2.23, -0.27]
TRA+ACE (Jirkof, P. 2018) MGS	2.97	1.32	8	2.57	0.84	8	0.6%	0.34 [-0.65, 1.33]
TRA+ACE (Jirkof, P. 2018) NEST	2.3	18.4	8	3.1	24.8	8	0.6%	-0.03 [-1.01, 0.95]
TRA+ACE (Jirkof, P. 2018) VS	2.35	1.89	8	3.86	3.16	8	0.6%	-0.55 [-1.55, 0.46]
TRA+ACE (Jirkof, P. 2018) VS	3.36	2.17	8	4.54	2	8	0.6%	-0.53 [-1.54, 0.47]
**Subtotal (95% CI)**			**496**			**460**	**37.2%**	**0.27 [0.04, 0.49]**
Heterogeneity: Tau² = 0.54; Chi² = 177.65, df = 69 (P < 0.00001); I² = 61%								
Test for overall effect: Z = 2.34 (P = 0.02)								
**Total (95% CI)**			**1583**			**1300**	**100.0%**	**0.45 [0.31, 0.60]**
Heterogeneity: Tau² = 0.64; Chi² = 552.62, df = 183 (P < 0.00001); I² = 67%								
Test for overall effect: Z = 6.14 (P < 0.00001)								
Test for subgroup differences: Chi² = 3.97, df = 1 (P = 0.05), I² = 74.8%								

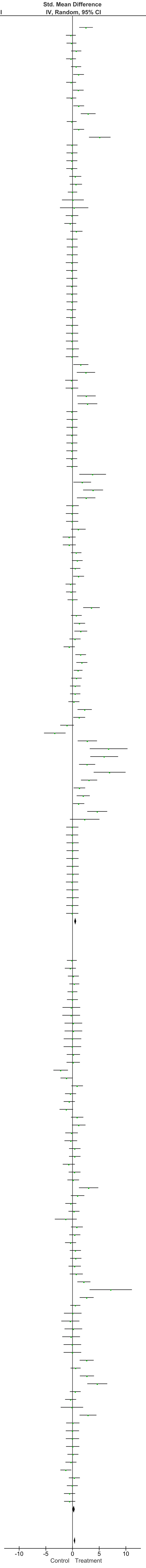

Supplementary figure 3. Subgroup analysis based on severity considering all analgesic treatments in all pain modalities. Forest plot presenting pooled SMDs (95% CI) stratified by surgical severity (mild vs. moderate). All analgesic treatment categories and pain modalities are included.

Study or Subgroup	Treatment Mean	Treatment SD	Treatment Total	Control Mean	Control SD	Control Total	Weight	Std. Mean Difference IV, Random, 95% CI
**3.1.1 Evoked**								
ACE30 (Zhu, Q. et al. 2016) VF	21.6	21.23	10	100	314	10	0.7%	-0.34 [-1.22, 0.55]
ACE50 (Cabañero & Maldonado. 2021) HT	3.2	2.01	10	3.58	1.69	10	0.7%	-0.20 [-1.08, 0.68]
ACE50 (Cabañero & Maldonado. 2021) VF	0.52	0.24	10	0.38	0.14	10	0.6%	0.68 [-0.23, 1.59]
ACE60 (Zhu, Q. et al. 2016) VF	28.32	37.41	10	100	314	10	0.7%	-0.31 [-1.19, 0.58]
ACE100 (Cabañero & Maldonado. 2021) HT	4.65	1.35	10	3.58	1.69	10	0.7%	0.67 [-0.24, 1.58]
ACE100 (Cabañero & Maldonado. 2021) VF	0.58	0.21	10	0.38	0.14	10	0.6%	1.07 [0.12, 2.03]
ACE120 (Zhu, Q. et al. 2016) VF	33.28	18.22	10	100	314	10	0.7%	-0.29 [-1.17, 0.59]
ACE200 (Cabañero & Maldonado. 2021) HT	6.3	2.94	10	3.58	1.69	10	0.6%	1.09 [0.13, 2.04]
ACE200 (Cabañero & Maldonado. 2021) VF	0.89	0.24	10	0.38	0.14	10	0.5%	2.49 [1.26, 3.71]
ACE240 (Zhu, Q. et al. 2016) VF	43.21	33.36	10	100	314	10	0.7%	-0.24 [-1.12, 0.64]
ACE300 (Cabañero & Maldonado. 2021) HT	1.03	0.28	10	0.38	0.14	10	0.5%	2.81 [1.50, 4.12]
ACE300 (Cabañero & Maldonado. 2021) VF	6.02	2.29	10	3.58	1.69	10	0.6%	1.16 [0.20, 2.13]
ACE360 (Zhu, Q. et al. 2016) VF	60.14	29.34	10	100	314	10	0.7%	-0.17 [-1.05, 0.71]
ACE400 (Cabañero & Maldonado. 2021) HT	1.28	0.21	10	0.38	0.14	10	0.4%	4.83 [2.94, 6.72]
ACE400 (Cabañero & Maldonado. 2021) VF	6.49	2.94	10	3.58	1.69	10	0.6%	1.16 [0.20, 2.13]
BUP0.1-0.15(MolinaCimadevila M. J. et al. 2014) HT	35.2	36.25	6	63	199	6	0.6%	-0.18 [-1.31, 0.96]
BUP0.1-0.3(MolinaCimadevila, M. J. et al. 2014) HT	1.3	0.47	7	3.17	1.61	7	0.5%	-1.48 [-2.71, -0.25]
BUP0.1(Oliver V; Thurston S; Logfren J. 2018) VF	13.76	29.75	6	63	199	6	0.6%	-0.32 [-1.46, 0.82]
BUP0.2 (Reddyjarugu, B. et al. 2015) HT	4.76	4.35	10	2.97	1.66	9	0.6%	0.51 [-0.41, 1.43]
BUP0.2 (Reddyjarugu, B. et al. 2015) VF	2.83	0.91	10	1.1	3.12	9	0.6%	0.74 [-0.20, 1.68]
BUP1.67x10³(Lu, F. et al. 2022) VF	5.35	2	12	6.26	3.07	8	0.7%	-0.35 [-1.26, 0.55]
BUP-SR1 (Navarro K, et al. 2020) HT	6.45	3.76	12	3.22	5.29	8	0.6%	0.70 [-0.23, 1.63]
BUP-SR1 (Navarro K, et al. 2020) VF	3.99	0.08	11	3.99	0.12	11	0.7%	0.00 [-0.84, 0.84]
BUP-XR3.25 (Navarro K, et al. 2020) HT	9.07	6.72	12	3.22	5.29	8	0.6%	0.90 [-0.04, 1.85]
BUP-XR3.25 (Navarro K, et al. 2020) VF	5.45	2.84	12	6.26	3.07	8	0.7%	-0.26 [-1.16, 0.63]
BUP-XR6.25 (Navarro K, et al. 2020) HT	8.47	3.76	12	3.22	5.29	8	0.6%	1.14 [0.16, 2.12]
BUP-XR6.25 (Navarro K, et al. 2020) VF	6.29	2.28	12	6.26	3.07	8	0.7%	0.01 [-0.88, 0.91]
CAR30 (Oliver V; Thurston S; Logfren J. 2018) VF	0.11	0.88	8	0.38	3.04	8	0.6%	-0.11 [-1.10, 0.87]
CEL1 (Muratani T. et al. 2005) VF	1.43	0.24	6	1.19	0.13	5	0.5%	1.10 [-0.22, 2.42]
CEL1 (Muratani T. et al. 2005) VF	5.65	2.34	6	5.89	3.24	6	0.6%	-0.08 [-1.21, 1.05]
CEL10 (Muratani T. et al. 2005) VF	5.96	1.62	6	5.89	3.24	6	0.6%	0.03 [-1.11, 1.16]
CEL10 (Muratani T. et al. 2005) VF	6.2	3	6	8.58	3.9	6	0.6%	-0.63 [-1.80, 0.54]
DEX100 (Romero-Alejo, E. et al. 2016) VF	0.9	0.11	10	0.36	0.18	10	0.5%	3.47 [1.98, 4.95]
DIC0.25 (Lu, F. et al. 2022) VF	3.84	0.69	11	3.99	0.12	11	0.7%	-0.29 [-1.13, 0.55]
DIC0.25 (Lu, F. et al. 2022) VF	1.29	0.8	11	1.57	1.56	11	0.7%	-0.22 [-1.06, 0.62]
FIR10 (Reddyjarugu, B. et al. 2015) HT	5.59	4.61	9	2.97	1.66	9	0.6%	0.72 [-0.24, 1.68]
FIR10 (Reddyjarugu, B. et al. 2015) VF	2.41	1.39	9	1.1	3.12	9	0.6%	0.52 [-0.43, 1.46]
FIR20 (Reddyjarugu, B. et al. 2015) HT	2.24	0.47	10	1.1	3.12	9	0.6%	0.50 [-0.42, 1.42]
FIR20 (Reddyjarugu, B. et al. 2015) VF	7.14	3.91	10	2.97	1.66	9	0.6%	1.30 [0.29, 2.31]
GABA20 (Yang J. et al. 2019) VF	0.28	0.15	10	0.05	0.03	9	0.6%	1.98 [0.83, 3.12]
GABA50 (Yang J. et al. 2019) VF	0.53	0.08	6	0.05	0.03	5	0.1%	6.98 [3.15, 10.81]
IBU100 (Saad, S. S. T. et al. 2016) VF	2.58	0.97	9	0.74	0.46	8	0.5%	2.25 [0.97, 3.54]
IBU300 (Saad, S. S. T. et al. 2016) VF	2.98	2.29	9	0.74	0.46	8	0.6%	1.25 [0.18, 2.32]
INDO1 (Brusco, I. et al. 2017) VF	3.23	0.79	6	1.44	1.27	6	0.5%	1.56 [0.20, 2.93]
INDO10 (Brusco, I. et al. 2017) VF	0.93	12.47	6	1.98	0.49	6	0.6%	-0.11 [-1.24, 1.02]
INDO10 (Brusco, I. et al. 2017) VF	4.3	0.48	6	1.77	1.07	6	0.4%	2.82 [1.04, 4.60]
INDO3 (Brusco, I. et al. 2017) VF	4.23	0.59	6	1.44	1.27	6	0.4%	2.60 [0.90, 4.30]
KET50 (Minville, V. et al. 2008) HT	0.19	3.04	16	0.48	7.68	4	0.6%	-0.07 [-1.16, 1.03]
KET50 (Minville, V. et al. 2008) VF	11.91	1.24	10	5.95	2.88	10	0.5%	2.57 [1.33, 3.82]
MOR1 (Clark, J. D. et al. 2007) VF	2.47	0.72	5	0.22	0.26	5	0.3%	3.75 [1.30, 6.20]
MOR1 (Dallazen, J. L et al. 2022) HT	12.27	3.74	6	4.48	1.35	6	0.4%	2.56 [0.87, 4.24]
MOR1 (Dallazen, J. L et al. 2022) VF	0.97	0.28	8	0.04	0.16	8	0.4%	3.86 [2.03, 5.68]
MOR3 (Minville, V. et al. 2008) HT	11.96	1.06	10	5.95	2.88	10	0.5%	2.65 [1.38, 3.92]
MOR3 (Minville, V. et al. 2008) VF	3.98	6.65	10	1.03	1.83	10	0.7%	0.58 [-0.32, 1.48]
MOR3 (Pogatzki, E. M. & Raja, S. N. 2003) HT	6.05	1.3	6	2.7	0.91	6	0.4%	2.76 [1.00, 4.51]
MOR3 (Tonello, R. et al. 2020) VF	2.62	5.68	6	7.63	2.94	6	0.5%	-1.02 [-2.26, 0.21]
MOR3* (Wei, S. et al. 2021) VF	2.79	0.44	8	0.32	0.35	8	0.2%	5.87 [3.33, 8.41]
MOR5* (Wei, S. et al. 2021) VF	3.49	1.52	8	0.32	0.35	8	0.5%	2.72 [1.26, 4.18]
MOR10 (Clark, J. D. et al. 2007) VF	0.66	0.18	5	0.22	0.26	5	0.4%	1.78 [0.18, 3.37]
MOR10* (Wei, S. et al. 2021) VF	5.52	0.95	8	0.32	0.35	8	0.2%	6.87 [3.96, 9.78]
MOR10 (Minville, V. et al. 2008) HT	11.95	0.88	10	5.95	2.88	10	0.5%	2.70 [1.42, 3.98]
MOR10 (Minville, V. et al. 2008) VF	8	0.91	10	1.03	1.83	10	0.4%	4.62 [2.80, 6.44]
MOR10 (Pogatzki, E. M. & Raja, S. N. 2003) HT	13.27	7.83	15	1.89	1.13	9	0.6%	1.75 [0.76, 2.74]
MOR10 (Pogatzki, E. M & Raja, S. N. 2003) VF	7.81	0.001	15	2.16	7.35	15	0.7%	1.06 [0.29, 1.83]
MOR10 (Tonello, R. et al. 2020) VF	7.99	0.49	6	2.7	0.91	6	0.1%	6.68 [3.23, 10.13]
MEL+BUP (Molina-Cimadevila, M. J. 2014) HT	35.36	37.48	2	63	199	2	0.3%	-0.11 [-2.17, 1.95]
OXY3 (Mueller, A. et al. 2019) VF	104.67	27.35	10	34.6	10.6	6	0.4%	2.90 [1.36, 4.44]
PRE30 (Park, J. H et al. 2020) VF	6.5	4	8	8.59	2.12	8	0.6%	-0.62 [-1.63, 0.39]
ROP5% (Yamada, T. et al. 2016) HT	18.11	7.21	9	10.54	3.22	9	0.6%	1.29 [0.25, 2.33]
ROP5% (Yamada, T. et al. 2016) HT	1.97	0.42	9	0.16	1.15	9	0.6%	1.99 [0.81, 3.17]
ROP5% (Yamada, T. et al. 2016) VF	1.88	1.95	9	0.1	0.76	9	0.6%	1.15 [0.13, 2.16]
ROP5% (Yamada, T. et al. 2016) VF	20.4	2.88	9	9.77	3.61	9	0.5%	3.10 [1.63, 4.57]
TRA100 (Romero-Alejo, E. et al. 2016) VF	2.13	3.42	8	3.64	6.05	8	0.6%	-0.29 [-1.28, 0.70]
TRA+DEXK (Romero-Alejo, E. 2016) VF	0.82	0.14	3	0.36	0.18	3	0.2%	2.28 [-0.44, 5.01]
**Subtotal (95% CI)**			**661**			**606**	**39.8%**	**1.12 [0.83, 1.41]**
Heterogeneity: Tau² = 1.20; Chi² = 348.21, df = 73 (P < 0.00001); I² = 79%								
Test for overall effect: Z = 7.56 (P < 0.00001)								
**3.1.2 Supressed**								
ACE+LID+BUV (Durst, M. S. 2021) BR	101.81	187.83	3	63	203.1	3	0.4%	0.16 [-1.45, 1.77]
ACE+LID+BUV (Durst, M. S. 2021) BR	101.2	153.35	3	68.7	156.8	3	0.4%	0.17 [-1.44, 1.78]
ACE+LID+BUV (Durst, M. S. 2021) NEST	0.7	1.27	3	1.11	1.38	3	0.4%	-0.25 [-1.87, 1.37]
ACE+LID+BUV (Durst, M. S. 2021) NEST	0.28	1.39	3	0.77	2.15	3	0.4%	-0.22 [-1.83, 1.40]
ACE200 (Durst, M. S. et al. 2021) BR	90.36	180.6	8	68.67	156.75	8	0.6%	0.12 [-0.86, 1.10]
ACE200 (Durst, M. S. et al. 2021) BR	126.06	149.48	10	63.03	203.14	10	0.7%	0.34 [-0.55, 1.22]
BUP+CAR (Adamsom, T, W) 2010 RW	0.17	0.18	2	0.15	0.13	2	0.3%	0.07 [-1.93, 2.08]
BUP+MEL+BUV (Furumoto, K. 2021) NEST	103.03	203.14	3	63	203.1	3	0.4%	0.16 [-1.45, 1.77]
BUP0.1 (Furumoto, K. et al. 2021) NEST	3.02	1.33	8	10.3	26.3	8	0.6%	-0.37 [-1.36, 0.62]
BUP0.1 HCl (Kendal, L. V. et al. 2016) NEST	69.3	41.5	7	62.9	46.8	7	0.6%	0.14 [-0.91, 1.18]
BUP0.1 HCl (Kendal, L. V. et al. 2016) RW	21.1	26.1	7	43.2	33.2	7	0.6%	-0.69 [-1.78, 0.40]
BUP0.2 (Adamson, T. W. et al. 2010) RW	0.5	0.87	7	0.15	0.13	7	0.6%	0.53 [-0.55, 1.60]
BUP0.2 (Clark, M. D. et al. 2004) RW	46.71	32.82	10	34.6	10.6	6	0.6%	0.42 [-0.60, 1.45]
BUP0.6 (Kendal, L. V. et al. 2016) NEST	94.1	8.6	6	62.8	46.8	7	0.6%	0.83 [-0.32, 1.99]
BUP0.6 (Kendal, L. V. et al. 2016) RW	9.5	16.1	6	43.2	33.2	7	0.5%	-1.17 [-2.39, 0.05]
CAR5 (Adamson, T. W. et al. 2010) RW	0.09	0.72	8	0.28	2.24	8	0.6%	-0.11 [-1.09, 0.87]
CAR5 (Jirkof, P. et al. 2013) NEST	3.9	1.63	8	3.05	1.39	8	0.6%	0.53 [-0.47, 1.53]
CAR50 (Jirkof, P. et al. 2013) NEST	0.68	1.27	8	0.1	0.002	8	0.6%	0.61 [-0.40, 1.62]
LID+BUV (Durst, M. S. 2021) NEST	0.7	1.27	3	1.11	1.38	3	0.4%	-0.25 [-1.87, 1.37]
LID+BUV (Durst, M. S. 2021) NEST	0.28	1.39	3	0.77	2.15	3	0.4%	-0.22 [-1.83, 1.40]
TRA+ACE (Jirkof, P. 2018) NEST	2.97	1.32	8	2.57	0.84	8	0.6%	0.34 [-0.65, 1.33]
BUP0.1 (Jirkof, P. et al. 2015) BR	0.37	0.76	8	0.88	0.84	8	0.6%	-0.60 [-1.61, 0.41]
BUP-SR2.2 (Jirkof, P. et al. 2015) BR	1.32	1.95	8	0.88	0.84	8	0.6%	0.28 [-0.71, 1.26]
CAR5 (Jirkof, P. et al. 2010) BR	0.06	0.48	8	0.25	2	8	0.6%	-0.12 [-1.10, 0.86]
LID+BUV (Durst, M. S. 2021) BR	103.03	203.14	3	63	203.1	3	0.4%	0.16 [-1.45, 1.77]
LID+BUV (Durst, M. S. 2021) BR	26.5	95.43	3	68.7	156.8	3	0.4%	-0.26 [-1.88, 1.36]
TRA25 (Evangelista-Vaz, R. et al. 2018) BR	214	383.82	8	191	156.41	8	0.6%	0.07 [-0.91, 1.05]
**Subtotal (95% CI)**			**162**			**160**	**14.4%**	**0.06 [-0.16, 0.28]**
Heterogeneity: Tau² = 0.00; Chi² = 14.91, df = 26 (P = 0.96); I² = 0%								
Test for overall effect: Z = 0.54 (P = 0.59)								
**3.1.3 Spontaneous**								
ACE+LID+BUV (Durst, M. S. 2021) MGS	0.58	2.18	3	0.59	6.3	3	0.4%	-0.00 [-1.60, 1.60]
ACE+LID+BUV (Durst, M. S. 2021) MGS	0.61	4.88	3	0.75	4.4	3	0.4%	-0.02 [-1.62, 1.58]
ACE200 (Durst, M. S. et al. 2021) MGS	0.63	6.3	10	0.73	6.3	10	0.7%	-0.02 [-0.89, 0.86]
ACE200 (Durst, M. S. et al. 2021) MGS	0.54	4.32	8	0.62	4.4	8	0.6%	-0.02 [-1.00, 0.96]
BUP+CAR (Adamsom, T. W. 2010) VS	1.4	0.55	2	1.15	0.37	2	0.2%	0.30 [-2.31, 2.92]
BUP0.009 (Jirkof, P. et al. 2019) MGS	4.8	19.2	8	2.06	19.04	4	0.5%	0.13 [-1.07, 1.33]
BUP0.009 (Jirkof, P. et al. 2019) MGS	3.85	15.4	8	1.28	12.4	4	0.5%	0.16 [-1.04, 1.37]
BUP0.01 (Wright-Williams, S. L. 2013) VS	0.18	2.88	16	0.48	7.68	4	0.6%	-0.07 [-1.17, 1.03]
BUP0.05 (Matsumya L. C, et al. 2012) MGS	1	0.79	16	0.48	7.68	4	0.6%	0.15 [-0.94, 1.25]
BUP0.05 (Wright-Williams, S. L. 2013) VS	2	2.72	8	4.97	2.26	8	0.6%	-1.12 [-2.20, -0.05]
BUP0.1 (Jirkof, P. et al. 2015) VS	0.01	1.76	16	0.48	7.68	4	0.6%	-0.10 [-1.20, 1.00]
BUP0.1 HCl (Kendal, L. V. et al. 2016) VS	1.25	0.5	7	1	0.7	7	0.6%	0.38 [-0.68, 1.45]
BUP0.2 (Adamson, T. W. et al. 2010) VS	1.71	1.08	7	1.15	0.37	7	0.6%	0.65 [-0.44, 1.73]
BUP0.2 (Clark, M. D. et al. 2004) VS	46.71	32.82	10	34.6	10.6	6	0.6%	0.42 [-0.60, 1.45]
BUP0.6 (Kendal, L. V. et al. 2016) VS	1.7	0.3	6	1	0.7	7	0.5%	1.17 [-0.04, 2.39]
BUP2 (Goecke, J. C. et al. 2005) VS	0.59	0.16	7	0.18	0.09	6	0.4%	2.87 [1.16, 4.58]
BUP-SR2.2 (Jirkof, P. et al. 2015) VS	4.08	2.57	8	10.3	26.3	8	0.6%	-0.31 [-1.30, 0.67]
BUV5 (Leach, M. C. et al. 2012) MGS	1.99	11.94	6	3.86	23.16	6	0.6%	-0.09 [-1.23, 1.04]
CAR10 (Cho, C. et al. 2019) MGS	8	0.38	7	8	0.13	7	0.6%	0.00 [-1.05, 1.05]
CAR10 (Cho, C. et al. 2019) MGS	8	0.25	7	8	0.37	7	0.6%	0.00 [-1.05, 1.05]
CAR10 (Cho, C. et al. 2019) MGS	248.38	110.34	8	191.93	136.44	8	0.6%	0.43 [-0.56, 1.43]
CAR10 (Cho, C. et al. 2019) MGS	35	41	7	10.41	5.06	8	0.6%	0.82 [-0.25, 1.89]
CAR20 (Matsumya L. C, et al. 2012) MGS	0.07	1.12	16	0.48	7.68	4	0.6%	-0.12 [-1.22, 0.98]
CAR25 (Cho, C. et al. 2019) MGS	0.18	1.44	8	0.42	3.36	8	0.6%	-0.09 [-1.07, 0.89]
CAR25 (Cho, C. et al. 2019) MGS	0.17	1.36	8	0.28	2.24	8	0.6%	-0.06 [-1.04, 0.92]
CAR25 (Cho, C. et al. 2019) MGS	0.24	1.92	8	0.38	3.04	8	0.6%	-0.05 [-1.03, 0.93]
CAR25 (Cho, C. et al. 2019) MGS	0.01	0.001	8	0.25	2	8	0.6%	-0.16 [-1.14, 0.82]
CAR25 (Matsumya L. C, et al. 2012) MGS	0.15	2.4	16	0.48	7.68	4	0.6%	-0.08 [-1.18, 1.01]
CAR5 (Adamson, T. W. et al. 2010) VS	0.06	0.48	8	0.25	2	8	0.6%	-0.12 [-1.10, 0.86]
CAR5 (Jirkof, P. et al. 2012) VS	0.15	2.4	16	0.48	7.68	4	0.6%	-0.08 [-1.18, 1.01]
INDO10 (Brusco, I. et al. 2017) SP	4.06	0.48	6	1.44	1.27	6	0.4%	2.52 [0.85, 4.19]
INDO10 (Brusco, I. et al. 2017) SP	5	21.9	6	8.94	6.89	6	0.6%	-0.20 [-1.33, 0.94]
KET20 (Matsumya L. C, et al. 2012) MGS	0.19	3.04	16	0.48	7.68	4	0.6%	-0.07 [-1.16, 1.03]
LID+BUV (Durst, M. S. 2021) MGS	0.41	4.1	3	0.58	6.3	3	0.4%	-0.03 [-1.63, 1.57]
LID+BUV (Durst, M. S. 2021) MGS	0.26	2.08	3	0.35	4.4	3	0.4%	-0.02 [-1.62, 1.58]
LID+BUV (Durst, M. S. 2021) NEST	0.4	1.41	3	1.11	1.38	3	0.4%	-0.41 [-2.06, 1.24]
LID+BUV (Durst, M. S. 2021) NEST	0.89	2.187	3	0.77	2.15	3	0.4%	0.04 [-1.56, 1.65]
MEL10 (Roughan, V. J. et al. 2009) VS	6.37	4.48	8	3.76	5.13	9	0.6%	0.51 [-0.46, 1.48]
MEL10 (Roughan, V. J. et al. 2016) VS	24.27	26.33	8	10.97	27.26	8	0.6%	0.47 [-0.53, 1.47]
MEL2(Cho, C. et al. 2019) MGS	0.11	0.88	8	0.42	3.36	8	0.6%	-0.12 [-1.10, 0.86]
MEL2 (Cho, C. et al. 2019) MGS	0.14	1.12	8	0.38	3.04	8	0.6%	-0.10 [-1.08, 0.88]
MEL2 (Cho, C. et al. 2019) MGS	0.08	0.64	8	0.38	3.04	8	0.6%	-0.13 [-1.11, 0.85]
MEL2 (Cho, C. et al. 2019) MGS	0.01	0.04	8	0.25	2	8	0.6%	-0.16 [-1.14, 0.82]
MEL20 (Leach, M. C. et al. 2012) MGS	2.35	14.1	6	3.86	23.16	6	0.6%	-0.07 [-1.20, 1.06]
MEL20 (Roughan, V. J. et al. 2009) VS	16.95	14.11	8	10.97	27.26	8	0.6%	0.26 [-0.72, 1.25]
MEL20 (Wright-Williams, S. L. 2009) VS	8.5	9.8	8	17.1	30.8	8	0.6%	-0.36 [-1.35, 0.63]
MEL5 (Cho, C. et al. 2019) MGS	0.2	1.6	8	0.42	3.36	8	0.6%	-0.08 [-1.06, 0.90]
MEL5 (Cho, C. et al. 2019) MGS	0.08	0.64	8	0.38	3.04	8	0.6%	-0.13 [-1.11, 0.85]
MEL5 (Cho, C. et al. 2019) MGS	0.06	0.48	8	0.28	2.24	8	0.6%	-0.13 [-1.11, 0.85]
MEL5 (Cho, C. et al. 2019) MGS	0.12	0.96	8	0.25	2	8	0.6%	-0.08 [-1.06, 0.90]
MEL5 (Roughan, V. J. et al. 2009) VS	50.54	20.7	8	10.97	27.26	8	0.6%	1.55 [0.39, 2.70]
MOR1 (Dallazen, J. L et al. 2022) SP	6.01	16.9	6	6.01	16.04	6	0.6%	0.00 [-1.13, 1.13]
MOR10 (Tonello, R. et al. 2020) SP	0.14	0.29	6	7.63	2.94	6	0.3%	-3.31 [-5.28, -1.34]
MOR3 (Tonello, R. et al. 2020) SP	6.33	3.42	15	1.89	1.13	9	0.6%	1.52 [0.57, 2.48]
OXYM-LE 2 (Clark, M. D. et al. 2004) VS	1.43	0.24	6	1.19	0.13	5	0.5%	1.10 [-0.22, 2.42]
TRA+ACE (Jirkof, P. 2018) MGS	2.3	18.4	8	3.1	24.8	8	0.6%	-0.03 [-1.01, 0.95]
TRA+ACE (Jirkof, P. 2018) VS	2.35	1.89	8	3.86	3.16	8	0.6%	-0.55 [-1.55, 0.46]
TRA+ACE (Jirkof, P. 2018) VS	3.36	2.17	8	4.54	2	8	0.6%	-0.53 [-1.54, 0.47]
TRA0.1 (Jirkof, P. et al. 2019) MGS	3.2	12.8	8	3.49	15.2	4	0.5%	-0.02 [-1.22, 1.18]
TRA0.1 (Jirkof, P. et al. 2019) MGS	2.93	11.72	8	2.21	14.72	4	0.5%	0.05 [-1.15, 1.25]
TRA1 (Jirkof, P. et al. 2019) MGS	3.66	14.64	8	3.22	10.08	4	0.5%	0.03 [-1.17, 1.23]
TRA1 (Jirkof, P. et al. 2019) MGS	3	12	8	3.18	19.36	4	0.5%	-0.01 [-1.21, 1.19]
TRA25 (Evangelista-Vaz, R. et al. 2018) VS	0.7	0.66	10	1.65	0.79	10	0.6%	-1.25 [-2.23, -0.27]
TRA80 (Wolfe, A. M. et al. 2015) VS	1.14	0.15	10	0.36	0.18	10	0.4%	4.51 [2.72, 6.30]
FLU2.5 (Goecke, J. C. et al. 2005) VS	0.23	0.13	7	0.18	0.09	6	0.6%	0.41 [-0.70, 1.52]
FLU2.5 (Goecke, J. C. et al. 2005) VS	0.27	0.15	6	0.18	0.09	6	0.6%	0.67 [-0.51, 1.85]
BUP2 (Goecke, J. C. et al. 2005) VS	0.38	0.27	6	0.18	0.09	6	0.5%	0.92 [-0.30, 2.13]
BUP 0.024 (Faller, K.M.E, 2015)	3.17	1.48	13	1.39	0.58	13	0.7%	1.53 [0.64, 2.43]
ACE 100 (Matsumya L. C, et al. 2012) MGS	0.57	0.56	16	0.48	7.68	4	0.6%	0.03 [-1.07, 1.12]
ACE 300 (Matsumya L. C, et al. 2012) MGS	0.37	0.72	16	0.48	7.68	4	0.6%	-0.03 [-1.13, 1.06]
ACE 450 (Matsumya L. C, et al. 2012) MGS	0.32	0.68	16	0.48	7.68	4	0.6%	-0.05 [-1.14, 1.05]
BUP0.001 (Matsumya L. C, et al. 2012) MGS	0.37	0.68	16	0.48	7.68	4	0.6%	-0.03 [-1.13, 1.06]
BUP0.01 (Matsumya L. C, et al. 2012) MGS	0.22	0.44	16	0.48	7.68	4	0.6%	-0.08 [-1.17, 1.02]
BUP0.1 (Matsumya L. C, et al. 2012) MGS	0.11	1.76	16	0.48	7.68	4	0.6%	-0.10 [-1.20, 1.00]
CAR 10 (Matsumya L. C, et al. 2012) MGS	0.52	0.2	16	0.48	7.68	4	0.6%	0.01 [-1.08, 1.11]
CAR 15 (Matsumya L. C, et al. 2012) MGS	0.55	0.36	16	0.48	7.68	4	0.6%	0.02 [-1.07, 1.12]
CAR 5 (Matsumya L. C, et al. 2012) MGS	0.43	0.52	16	0.48	7.68	4	0.6%	-0.02 [-1.11, 1.08]
KETO 1 (Matsumya L. C, et al. 2012) MGS	0.53	0.56	16	0.48	7.68	4	0.6%	0.02 [-1.08, 1.11]
KETO 10 (Matsumya L. C, et al. 2012) MGS	0.64	0.6	16	0.48	7.68	4	0.6%	0.05 [-1.05, 1.14]
KETO 15 (Matsumya L. C, et al. 2012) MGS	0.18	0.44	16	0.48	7.68	4	0.6%	-0.09 [-1.19, 1.01]
KETO 5 (Matsumya L. C, et al. 2012) MGS	0.43	0.2	16	0.48	7.68	4	0.6%	-0.02 [-1.11, 1.08]
**Subtotal (95% CI)**			**765**			**491**	**45.8%**	**0.12 [-0.03, 0.27]**
Heterogeneity: Tau² = 0.13; Chi² = 112.61, df = 80 (P = 0.010); I² = 29%								
Test for overall effect: Z = 1.62 (P = 0.10)								
**Total (95% CI)**			**1588**			**1257**	**100.0%**	**0.48 [0.33, 0.62]**
Heterogeneity: Tau² = 0.63; Chi² = 535.35, df = 181 (P < 0.00001); I² = 66%								
Test for overall effect: Z = 6.46 (P < 0.00001)								
Test for subgroup differences: Chi² = 40.21, df = 2 (P < 0.00001), I² = 95.0%								

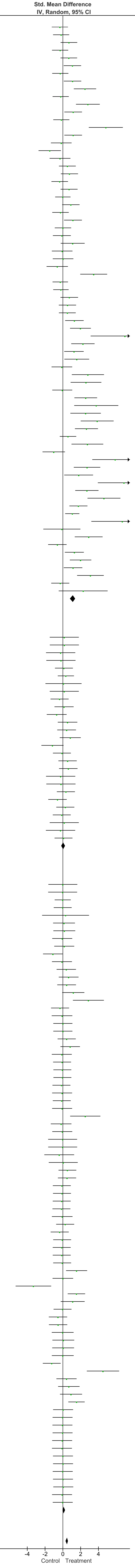

Supplementary figure 4. Meta-analysis based on the studies included in the meta-analysis of all analgesic treatments subgrouped by pain modality. Forest plot showing pooled SMDs (95% CI) for all analgesic treatments grouped by pain modality (evoked, spontaneous, and behaviors suppressed by pain). The analysis synthesizes all treatment categories and identifies differences among modalities.

Study or Subgroup	Treatment Mean	Treatment SD	Treatment Total	Control Mean	Control SD	Control Total	Weight	Std. Mean Difference IV, Random, 95% CI
**2.2.1 Mechanically Evoked**								
ACE30 (Zhu, Q. 2016)	21.6	19.38	10	100	316.23	10	1.6%	-0.34 [-1.22, 0.55]
ACE50 (Cabañero & Maldonado, 2021)	0.52	0.22	10	0.38	0.13	10	1.6%	0.74 [-0.17, 1.66]
ACE60(Zhu, Q. 2016)	28.32	34.15	10	100	316.23	10	1.6%	-0.31 [-1.19, 0.58]
ACE100 (Cabañero & Maldonado, 2021)	0.58	0.19	10	0.38	0.13	10	1.6%	1.18 [0.21, 2.14]
ACE120 (Zhu, Q. 2016)	33.28	16.63	10	100	316.23	10	1.6%	-0.29 [-1.17, 0.60]
ACE240 (Zhu, Q. 2016)	43.21	30.45	10	100	316.23	10	1.6%	-0.24 [-1.12, 0.64]
ACE200 (Cabañero & Maldonado, 2021)	0.89	0.22	10	0.38	0.13	10	1.4%	2.70 [1.42, 3.98]
ACE300 (Cabañero & Maldonado, 2021)	1.03	0.25	10	0.38	0.13	10	1.3%	3.12 [1.73, 4.52]
ACE360 (Zhu, Q. 2016)	60.14	26.78	10	100	316.23	10	1.6%	-0.17 [-1.05, 0.71]
ACE400 (Cabañero & Maldonado 2021)	1.28	0.19	10	0.38	0.13	10	1.0%	5.30 [3.26, 7.33]
BUP0.1 (Oliver, V. L; 2018)	4.31	1.1	8	3.05	1.13	8	1.5%	1.07 [-0.00, 2.14]
BUP0.2 (Reddyjarugu, B. 2015)	2.83	0.79	10	1.1	2.7	9	1.6%	0.85 [-0.10, 1.80]
BUP1 SR (Navarro K. 2020)	5.35	1.73	12	6.26	2.66	8	1.6%	-0.41 [-1.31, 0.50]
BUP1.67 (Lu, F. 2022)	3.99	0.07	11	3.99	0.1	11	1.6%	0.00 [-0.84, 0.84]
BUP3.25 XR (Navarro K, et al. 2020)	5.45	2.46	12	6.26	2.66	8	1.6%	-0.31 [-1.21, 0.60]
BUP6.5 XR (Navarro K, et al. 2020)	6.29	1.97	12	6.26	2.66	8	1.6%	0.01 [-0.88, 0.91]
CAR10 (Oliver, V. L; 2018)	3.9	1.33	8	3.05	1.13	8	1.5%	0.65 [-0.36, 1.67]
CEL1 (Muratani T. 2005)	5.65	1.91	6	5.89	2.65	6	1.5%	-0.10 [-1.23, 1.04]
CEL1 (Muratani T. 2005)	6.2	2.45	6	8.58	3.18	6	1.4%	-0.77 [-1.97, 0.42]
CEL10 (Muratani T. 2005)	5.96	1.32	6	5.89	2.65	6	1.5%	0.03 [-1.10, 1.16]
CEL10 (Muratani T. 2005)	6.5	1.89	6	8.58	3.18	6	1.5%	-0.73 [-1.92, 0.45]
DIC0.25 (Lu, F. 2022)	3.84	0.56	11	3.99	0.1	11	1.6%	-0.36 [-1.20, 0.49]
DIC0.25 (Lu, F. 2022)	1.29	0.8	11	1.57	1.56	11	1.6%	-0.22 [-1.06, 0.62]
DKET100 (Romero-Alejo, E., et al. 2016)	0.9	0.09	10	0.36	0.16	10	1.2%	3.98 [2.35, 5.62]
FIR10 (Reddyjarugu, B. 2015)	2.41	1.2	9	1.1	2.7	9	1.6%	0.60 [-0.35, 1.55]
FIR20 (Reddyjarugu, B. 2015)	2.24	0.41	10	1.1	2.7	9	1.6%	0.58 [-0.34, 1.50]
GABA20 (Yang J. 2019)	0.28	0.13	10	0.051	0.03	8	1.4%	2.19 [0.96, 3.43]
GABA50 (Yang J. 2019)	0.53	0.07	6	0.051	0.03	8	0.5%	8.85 [4.84, 12.86]
IBU100 (Saad, S. S. T. 2016)	2.58	0.84	9	0.74	0.4	8	1.4%	2.60 [1.22, 3.98]
IBU300 (Saad, S. S. T. 2016)	2.98	1.98	9	0.74	0.4	8	1.5%	1.44 [0.34, 2.55]
INDO1 (Brusco, I. 2017)	3.23	0.69	6	1.44	1.1	6	1.3%	1.80 [0.37, 3.23]
INDO3 (Brusco, I. 2017)	4.06	0.42	6	1.44	1.1	6	1.1%	2.90 [1.09, 4.72]
INDO10 (Brusco, I. 2017)	4.23	0.51	6	1.44	1.1	6	1.1%	3.00 [1.15, 4.86]
INDO10-post (Brusco, I. 2017)	4.3	0.34	6	1.77	0.76	6	0.9%	3.97 [1.72, 6.21]
KET50 (Minville, V. 2008)	3.99	6.36	10	1.03	1.58	10	1.6%	0.61 [-0.29, 1.51]
MOR1 (Clark, J. D. 2007)	2.47	0.63	5	0.22	0.22	5	0.8%	4.31 [1.58, 7.03]
MOR1 (Dallazen, J. L. 2022)	0.97	0.2	8	0.04	0.11	8	0.9%	5.45 [3.06, 7.83]
MOR3 (Minville, V. 2008)	3.98	5.76	10	1.03	1.58	10	1.6%	0.67 [-0.24, 1.58]
MOR3 (Tonello, R. 2020)	6.05	1.3	6	2.7	0.91	6	1.2%	2.76 [1.00, 4.51]
MOR3* (Wei, S. 2021)	2.79	0.4	8	0.32	0.31	8	0.7%	6.53 [3.74, 9.31]
MOR5* (Wei, S. 2021)	3.49	1.36	8	0.32	0.31	8	1.3%	3.04 [1.48, 4.60]
MOR10 (Clark, J. D. 2007)	0.66	0.16	5	0.22	0.22	5	1.2%	2.07 [0.37, 3.77]
MOR10 (Minville, V. 2008)	8	0.79	10	1.03	1.58	10	1.0%	5.34 [3.30, 7.39]
MOR10 (Pogatzki & Raja 2003)	7.81	0.001	15	2.16	6	15	1.6%	1.30 [0.50, 2.09]
MOR10 (Tonello, R. 2020)	7.99	0.49	6	2.7	0.91	6	0.6%	6.68 [3.23, 10.13]
MOR10* (Wei, S. 2021)	5.52	0.85	8	0.32	0.31	8	0.6%	7.68 [4.47, 10.90]
OXY3 (Mueller, A. 2019)	1.43	0.17	6	1.19	0.09	5	1.3%	1.57 [0.12, 3.01]
PGA20 (Park, J. H. 2020)	6.5	2.83	8	8.59	1.5	8	1.5%	-0.87 [-1.91, 0.17]
ROPI5% (Yamada, T. 2016)	1.97	0.3	9	0.16	0.81	9	1.3%	2.82 [1.43, 4.22]
ROPI5% (Yamada, T. 2016)	1.88	1.38	9	0.1	0.54	9	1.5%	1.62 [0.52, 2.72]
TRA100 (Romero-Alejo, E. 2016)	1.14	0.13	10	0.36	0.16	10	1.1%	5.12 [3.15, 7.10]
**Subtotal (95% CI)**			**447**			**430**	**68.4%**	**1.52 [1.10, 1.95]**
Heterogeneity: Tau² = 1.86; Chi² = 320.91, df = 50 (P < 0.00001); I² = 84%								
Test for overall effect: Z = 7.04 (P < 0.00001)								
**2.2.2 Heat Evoked**								
ACE50 (Cabañero & Maldonado, 2021)	3.2	2.01	10	3.58	1.7	10	1.6%	-0.20 [-1.07, 0.68]
ACE100 (Cabañero & Maldonado, 2021)	4.65	1.35	10	3.58	1.7	10	1.6%	0.67 [-0.24, 1.57]
ACE200 (Cabañero & Maldonado, 2021)	6.3	2.94	10	3.58	1.7	10	1.6%	1.08 [0.13, 2.04]
ACE300 (Cabañero & Maldonado, 2021)	6.02	2.29	10	3.58	1.7	10	1.6%	1.16 [0.19, 2.12]
ACE400 (Cabañero & Maldonado 2021)	6.49	2.94	10	3.58	1.7	10	1.6%	1.16 [0.20, 2.12]
BUP 0.1; 0.15 (Molina-Cimadevila, M. J. 2014)	13.76	29.75	6	-63.21	199.05	6	1.5%	0.50 [-0.66, 1.66]
BUP 0.1; 0.3 (Molina-Cimadevila, M. J. 2014)	35.2	36.25	6	-63.21	199.05	6	1.5%	0.63 [-0.54, 1.81]
BUP0.2 (Reddyjarugu, B. 2015)	4.76	4.35	10	2.97	1.66	9	1.6%	0.51 [-0.41, 1.43]
BUP1 SR (Navarro K. 2020)	6.45	3.76	12	3.22	5.29	8	1.6%	0.70 [-0.23, 1.63]
BUP3.25 XR (Navarro K, et al. 2020)	9.07	6.72	12	3.22	5.29	8	1.6%	0.90 [-0.04, 1.85]
BUP6.5 XR (Navarro K, et al. 2020)	8.47	3.76	12	3.22	5.29	8	1.6%	1.14 [0.16, 2.12]
FIR10 (Reddyjarugu, B. 2015)	5.59	4.61	9	2.97	1.66	9	1.6%	0.72 [-0.24, 1.68]
FIR20 (Reddyjarugu, B. 2015)	7.14	3.91	10	2.97	1.66	9	1.5%	1.30 [0.29, 2.31]
KET50 (Minville, V. 2008)	11.91	1.24	10	5.95	2.88	10	1.4%	2.57 [1.33, 3.82]
MOR1 (Dallazen, J. L. 2022)	12.27	3.74	6	4.48	1.35	6	1.2%	2.56 [0.87, 4.24]
MOR3 (Minville, V. 2008)	11.96	1.06	10	5.95	2.88	10	1.4%	2.65 [1.38, 3.92]
MOR3 (Pogatzki & Raja 2003)	6.33	3.42	15	1.89	1.14	9	1.6%	1.52 [0.57, 2.48]
MOR10 (Minville, V. 2008)	11.95	0.88	10	5.95	2.88	10	1.4%	2.70 [1.42, 3.98]
MOR10 (Pogatzki & Raja 2003)	13.27	7.83	15	1.89	1.14	9	1.6%	1.75 [0.76, 2.74]
ROPI5% (Yamada, T. 2016)	20.4	2.88	9	9.77	3.61	9	1.3%	3.10 [1.63, 4.57]
ROPI5% (Yamada, T. 2016)	18.11	7.21	9	10.54	3.22	9	1.5%	1.29 [0.25, 2.33]
**Subtotal (95% CI)**			**211**			**185**	**31.6%**	**1.25 [0.91, 1.58]**
Heterogeneity: Tau² = 0.33; Chi² = 43.44, df = 20 (P = 0.002); I² = 54%								
Test for overall effect: Z = 7.24 (P < 0.00001)								
**Total (95% CI)**			**658**			**615**	**100.0%**	**1.40 [1.09, 1.71]**
Heterogeneity: Tau² = 1.36; Chi² = 371.01, df = 71 (P < 0.00001); I² = 81%								
Test for overall effect: Z = 8.87 (P < 0.00001)								
Test for subgroup differences: Chi² = 1.00, df = 1 (P = 0.32), I² = 0.3%								

Std. Mean Difference
IV, Random, 95% CI

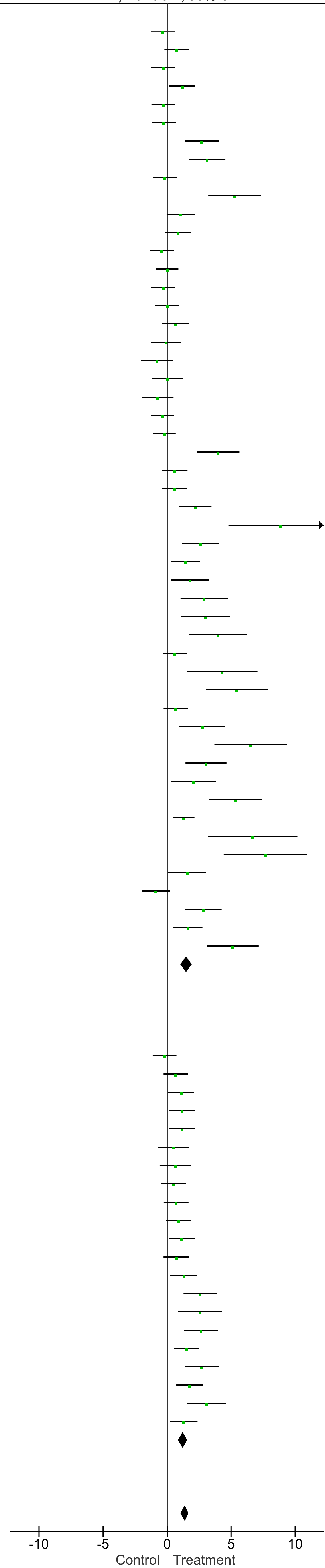

Supplementary figure 5. Meta-analysis based on the studies included in the meta-analysis of all analgesic treatments subgrouped by evoked pain modality. Forest plot showing pooled SMDs (95% CI) for all analgesic treatments evaluated exclusively within the evoked pain modality.

Study or Subgroup	Treatment Mean	Treatment SD	Treatment Total	Control Mean	Control SD	Control Total	Weight	Std. Mean Difference IV, Random, 95% CI
**2.3.1 Mouse Grimace Scale**								
ACE200 (Durst, M. S. 2021)	0.54	4.32	8	0.55	4.4	8	1.8%	-0.00 [-0.98, 0.98]
ACE200 (Durst, M. S. 2021)	0.63	6.3	10	0.68	6.8	10	2.0%	-0.01 [-0.88, 0.87]
BUP0.009 (Jirkof, P. 2019)	4.8	19.2	8	2.06	19.04	4	1.3%	0.13 [-1.07, 1.33]
BUP0.009 (Jirkof, P. 2019)	3.85	15.4	8	1.28	12.4	4	1.3%	0.16 [-1.04, 1.37]
BUP0.05 (Matsumya L. C. 2012)	0.18	2.88	16	0.48	7.68	4	1.5%	-0.07 [-1.17, 1.03]
BUP0.1 (Cho, C. 2019)	0.18	1.44	8	0.42	3.36	8	1.8%	-0.09 [-1.07, 0.89]
BUP0.1 (Cho, C. 2019)	0.07	0.56	8	0.28	2.24	8	1.8%	-0.12 [-1.10, 0.86]
BUP0.1 (Cho, C. 2019)	0.13	1.04	8	0.38	3.04	8	1.8%	-0.10 [-1.08, 0.88]
BUP 0.1 (Cho, C. 2019)	0.04	0.32	8	0.25	2	8	1.8%	-0.14 [-1.12, 0.84]
BUP0.1 (Matsumya L. C. 2012)	0.11	1.76	16	0.48	7.68	4	1.5%	-0.10 [-1.20, 1.00]
BUV5 (Leach, M. C. 2012)	1.99	11.94	6	3.86	23.16	6	1.5%	-0.09 [-1.23, 1.04]
CAR10 (Cho, C. 2019)	0.18	1.44	8	0.42	3.36	8	1.8%	-0.09 [-1.07, 0.89]
CAR10 (Cho, C. 2019)	0.17	1.36	8	0.28	2.24	8	1.8%	-0.06 [-1.04, 0.92]
CAR10 (Cho, C. 2019)	0.24	1.92	8	0.38	3.04	8	1.8%	-0.05 [-1.03, 0.93]
CAR10 (Cho, C. 2019)	0.001	0.001	8	0.25	2	8	1.8%	-0.17 [-1.15, 0.82]
CAR20 (Matsumya L. C. 2012)	0.07	1.12	16	0.48	7.68	4	1.5%	-0.12 [-1.22, 0.98]
CAR25 (Cho, C. 2019)	0.05	0.4	8	0.42	3.36	8	1.8%	-0.15 [-1.13, 0.84]
CAR25 (Cho, C. 2019)	0.09	0.72	8	0.28	2.24	8	1.8%	-0.11 [-1.09, 0.87]
CAR 25 (Cho, C. 2019)	0.11	0.88	8	0.38	3.04	8	1.8%	-0.11 [-1.10, 0.87]
CAR 25 (Cho, C. 2019)	0.06	0.48	8	0.25	2	8	1.8%	-0.12 [-1.10, 0.86]
CAR25 (Matsumya L. C. 2012)	0.15	2.4	16	0.48	7.68	4	1.5%	-0.08 [-1.18, 1.01]
KET20 (Matsumya L. C. 2012)	0.19	3.04	16	0.48	7.68	4	1.5%	-0.07 [-1.16, 1.03]
MEL2 (Cho, C. 2019)	0.11	0.88	8	0.42	3.36	8	1.8%	-0.12 [-1.10, 0.86]
MEL 2 (Cho, C. 2019)	0.08	0.64	8	0.28	2.24	8	1.8%	-0.11 [-1.10, 0.87]
MEL 2 (Cho, C. 2019)	0.14	1.12	8	0.38	3.04	8	1.8%	-0.10 [-1.08, 0.88]
MEL 2 (Cho, C. 2019)	0.005	0.04	8	0.25	2	8	1.8%	-0.16 [-1.15, 0.82]
MEL5 (Cho, C. 2019)	0.2	1.6	8	0.42	3.36	8	1.8%	-0.08 [-1.06, 0.90]
MEL5 (Cho, C. 2019)	0.06	0.48	8	0.28	2.24	8	1.8%	-0.13 [-1.11, 0.85]
MEL 5 (Cho, C. 2019)	0.08	0.64	8	0.38	3.04	8	1.8%	-0.13 [-1.11, 0.85]
MEL 5 (Cho, C. 2019)	0.12	0.96	8	0.25	2	8	1.8%	-0.08 [-1.06, 0.90]
MEL20 (Leach, M. C. 2012)	2.35	14.1	6	3.86	23.16	6	1.5%	-0.07 [-1.20, 1.06]
TRA0.1 (Jirkof, P. 2019)	2.93	11.72	8	2.21	14.72	4	1.4%	0.05 [-1.15, 1.25]
TRA0.1 (Jirkof, P. 2019)	3.2	12.8	8	3.49	15.2	4	1.4%	-0.02 [-1.22, 1.18]
TRA1 (Jirkof, P. 2019)	3	12	8	3.18	19.36	4	1.4%	-0.01 [-1.21, 1.19]
TRA1 (Jirkof, P. 2019)	3.66	14.64	8	3.22	10.08	4	1.4%	0.03 [-1.17, 1.23]
BUP 0.024 (Faller, K.M.E, 2015) MGS	3.17	1.48	13	1.39	0.58	13	2.0%	1.53 [0.64, 2.43]
**Subtotal (95% CI)**			**331**			**247**	**59.8%**	**-0.02 [-0.19, 0.15]**
Heterogeneity: Tau² = 0.00; Chi² = 12.66, df = 35 (P = 1.00); I² = 0%								
Test for overall effect: Z = 0.22 (P = 0.83)								
**2.3.2 Spontaneous/Guarding Assessment**								
INDO1 (Brusco, I. 2017)	5.5	21.89	6	2.05	16.87	6	1.5%	0.16 [-0.97, 1.30]
INDO10-post (Brusco, I. 2017)	0.93	12.46	6	1.49	1.2	6	1.5%	-0.06 [-1.19, 1.07]
MOR1 (Dallazen, J. L. 2022)	6.01	16.99	8	16.04	45.36	8	1.8%	-0.28 [-1.26, 0.71]
MOR3 (Tonello, R. 2020)	2.62	5.68	6	7.63	2.93	6	1.3%	-1.02 [-2.26, 0.21]
MOR10 (Tonello, R. 2020)	0.14	0.29	6	7.63	2.93	6	0.6%	-3.32 [-5.30, -1.34]
**Subtotal (95% CI)**			**32**			**32**	**6.6%**	**-0.67 [-1.57, 0.23]**
Heterogeneity: Tau² = 0.64; Chi² = 10.60, df = 4 (P = 0.03); I² = 62%								
Test for overall effect: Z = 1.47 (P = 0.14)								
**2.3.3 Clinical and Behavioral Investigation**								
BUP0.05 (Wright-Williams, S. L. 2013)	2	2.72	8	4.97	2.26	8	1.6%	-1.12 [-2.20, -0.05]
BUP0.01 (Wright-Williams, S. L. 2013)	1	0.79	8	4.97	2.26	8	1.2%	-2.22 [-3.54, -0.90]
BUP0.1 (Kendal, L. V. 2016)	1.25	0.5	7	1	0.7	7	1.6%	0.38 [-0.68, 1.45]
BUP0.2 (Adamson, T. W. 2010)	1.71	1.08	7	1.15	0.37	7	1.6%	0.65 [-0.44, 1.73]
BUP0.1 (Jirkof, P. 2015)	0.37	0.76	8	0.88	0.85	8	1.7%	-0.60 [-1.61, 0.41]
BUP0.2 (Clark, M. D. 2004)	46.71	32.82	10	34.6	10.61	6	1.7%	0.42 [-0.60, 1.45]
BUP0.6 SR (Kendal, L. V. 2016)	1.7	0.3	6	1	0.7	7	1.3%	1.17 [-0.04, 2.39]
BUP2.2 SR (Jirkof, P. 2015)	1.32	1.95	8	0.88	0.85	8	1.8%	0.28 [-0.71, 1.26]
BUP2 (Goecke, J. C. 2005)	0.59	0.16	7	0.18	0.1	6	0.8%	2.80 [1.11, 4.49]
BUP2 (Goecke, J. C. 2005)	0.38	0.27	6	0.18	0.1	6	1.3%	0.91 [-0.31, 2.12]
OXY2 (Clark, M. D. 2004)	104.67	27.35	10	34.6	10.61	6	0.9%	2.90 [1.36, 4.44]
TRA25 (Evangelista-Vaz, R. 2018)	2.13	3.42	8	3.64	6.05	8	1.8%	-0.29 [-1.28, 0.70]
TRA80 (Wolfe, A. M. 2015)	0.7	0.66	10	1.65	0.79	10	1.8%	-1.25 [-2.23, -0.27]
CAR5 (Adamson, T. W. 2010)	0.96	0.48	7	1.15	0.37	7	1.6%	-0.42 [-1.48, 0.65]
CAR5 (Jirkof, P. 2010)	248.38	110.34	8	192	136.44	8	1.7%	0.43 [-0.57, 1.42]
FLU2.5 (Goecke, J. C. 2005)	0.23	0.13	7	0.18	0.1	6	1.5%	0.40 [-0.71, 1.50]
FLU2.5 (Goecke, J. C. 2005)	0.27	0.15	6	0.18	0.1	6	1.4%	0.65 [-0.52, 1.83]
MEL20 (Roughan, V. J. 2009)	16.95	14.11	8	11	27.27	8	1.8%	0.26 [-0.73, 1.24]
MEL20 (Wright-Williams, S. L. 2007)	8.5	9.8	8	17.1	30.8	8	1.7%	-0.36 [-1.35, 0.63]
MEL5 (Roughan, V. J. 2009)	50.54	20.7	8	11	27.27	8	1.4%	1.54 [0.39, 2.70]
MEL10 (Roughan, V. J. 2009)	24.27	26.33	8	11	27.27	8	1.7%	0.47 [-0.53, 1.47]
MEL20 (Roughan, V. J. 2016)	6.37	4.86	8	3.76	5.13	9	1.8%	0.49 [-0.48, 1.47]
**Subtotal (95% CI)**			**171**			**163**	**33.6%**	**0.27 [-0.14, 0.69]**
Heterogeneity: Tau² = 0.65; Chi² = 65.40, df = 21 (P < 0.00001); I² = 68%								
Test for overall effect: Z = 1.30 (P = 0.19)								
**Total (95% CI)**			**534**			**442**	**100.0%**	**0.03 [-0.14, 0.19]**
Heterogeneity: Tau² = 0.15; Chi² = 94.63, df = 62 (P = 0.005); I² = 34%								
Test for overall effect: Z = 0.32 (P = 0.75)								
Test for subgroup differences: Chi² = 3.90, df = 2 (P = 0.14), I² = 48.7%								

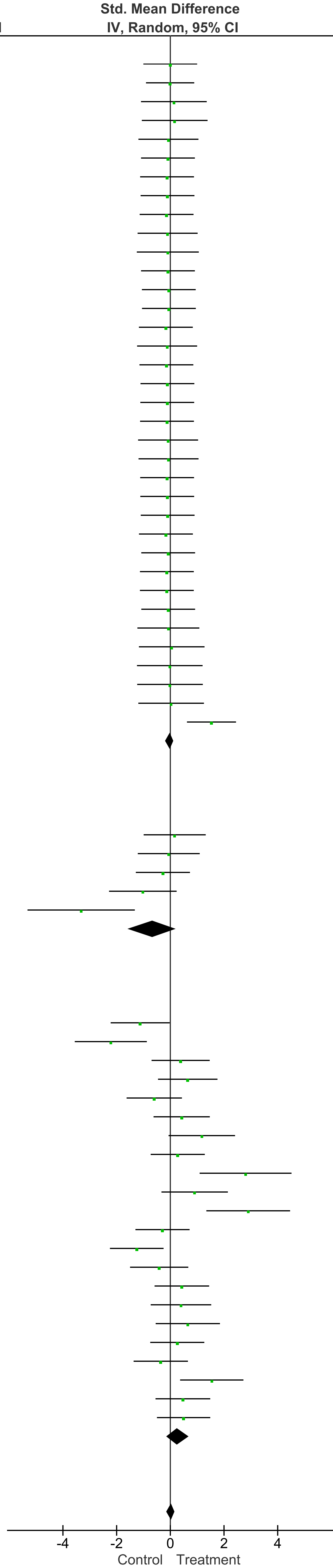

Supplementary figure 6. Meta-analysis based on the studies included in the meta-analysis of all analgesic treatments subgrouped by spontaneous pain modality. Forest plot presenting pooled SMDs (95% CI) for all analgesic treatments assessed in spontaneous pain outcomes.

Study or Subgroup	Experimental Mean	Experimental SD	Experimental Total	Control Mean	Control SD	Control Total	Weight	Std. Mean Difference IV, Random, 95% CI
**2.4.1 Burrowing**								
ACE200 (Durst, M. S. 2021)	90.36	180.595	8	68.67	156.751431	8	7.0%	0.12 [-0.86, 1.10]
BUP0.1 (Jirkof, P. 2015)	3.02	1.32936	8	5.75	3.59210245	8	6.9%	-0.95 [-2.00, 0.10]
BUP2.2 SR (Jirkof, P. 2015)	4.08	2.57387	8	5.75	3.59210245	8	7.0%	-0.51 [-1.51, 0.50]
CAR5 (Jirkof, P. 2010)	35	41.5383	7	10.3	26.3043723	8	6.9%	0.68 [-0.37, 1.73]
TRA25 (Evangelista-Vaz, R. 2018)	214	383.818	8	191	156.41202	8	7.0%	0.07 [-0.91, 1.05]
**Subtotal (95% CI)**			**39**			**40**	**34.9%**	**-0.11 [-0.65, 0.42]**
Heterogeneity: Tau² = 0.11; Chi² = 5.58, df = 4 (P = 0.23); I² = 28%								
Test for overall effect: Z = 0.42 (P = 0.68)								
**2.4.2 Locomotor Activity**								
BUP0.1 (Kendal, L. V. 2016)	21.1	2.64	7	43.3	2.65	7	2.6%	-7.86 [-11.45, -4.27]
BUP0.2 (Adamson, T. W. 2010)	0.5	0.87	7	0.15	0.13	7	6.9%	0.53 [-0.55, 1.60]
BUP0.2 (Clark, M. D. 2004)	46.71	3.16	10	34.6	2.44	6	5.2%	3.92 [2.05, 5.78]
BUP0.6 SR (Kendal, L. V. 2016)	9.5	2.44	6	43.2	2.64	7	1.3%	-12.29 [-18.05, -6.53]
CAR5 (Adamson, T. W. 2010)	0.31	0.26	7	0.15	0.13	7	6.8%	0.73 [-0.37, 1.82]
OXY2 (Clark, M. D. 2004)	104.67	3.16	10	34.6	2.44	6	0.6%	22.66 [13.56, 31.76]
**Subtotal (95% CI)**			**47**			**40**	**23.2%**	**-0.04 [-3.34, 3.26]**
Heterogeneity: Tau² = 13.25; Chi² = 75.29, df = 5 (P < 0.00001); I² = 93%								
Test for overall effect: Z = 0.02 (P = 0.98)								
**2.4.3 Nest Building**								
ACE200 (Durst, M. S. 2021)	0.52	1.549516	10	0.77	2.150349	10	7.2%	-0.13 [-1.01, 0.75]
ACE200 (Durst, M. S. 2021)	0.5	1.499066	8	1.11	1.385929	8	7.0%	-0.40 [-1.39, 0.59]
BUP0.1 (Kendal, L. V. 2016)	69.3	41.5	7	62.9	46.9	8	7.0%	0.14 [-0.88, 1.15]
BUP0.6 SR (Kendal, L. V. 2016)	94.1	8.6	6	62.8	46.8	7	6.7%	0.83 [-0.32, 1.99]
CAR5 (Jirkof, P. 2013)	0.04	0.226274	8	0.01	0.002828	8	7.0%	0.18 [-0.81, 1.16]
CAR50 (Jirkof, P. 2013)	0.68	1.272792	8	0.01	0.002828	8	7.0%	0.70 [-0.32, 1.72]
**Subtotal (95% CI)**			**47**			**49**	**41.9%**	**0.17 [-0.23, 0.58]**
Heterogeneity: Tau² = 0.00; Chi² = 4.02, df = 5 (P = 0.55); I² = 0%								
Test for overall effect: Z = 0.83 (P = 0.41)								
**Total (95% CI)**			**133**			**129**	**100.0%**	**0.10 [-0.60, 0.81]**

Heterogeneity: Tau² = 1.57; Chi² = 88.70, df = 16 (P < 0.00001); I² = 82%
Test for overall effect: Z = 0.29 (P = 0.77)
Test for subgroup differences: Chi² = 0.70, df = 2 (P = 0.70), I² = 0%

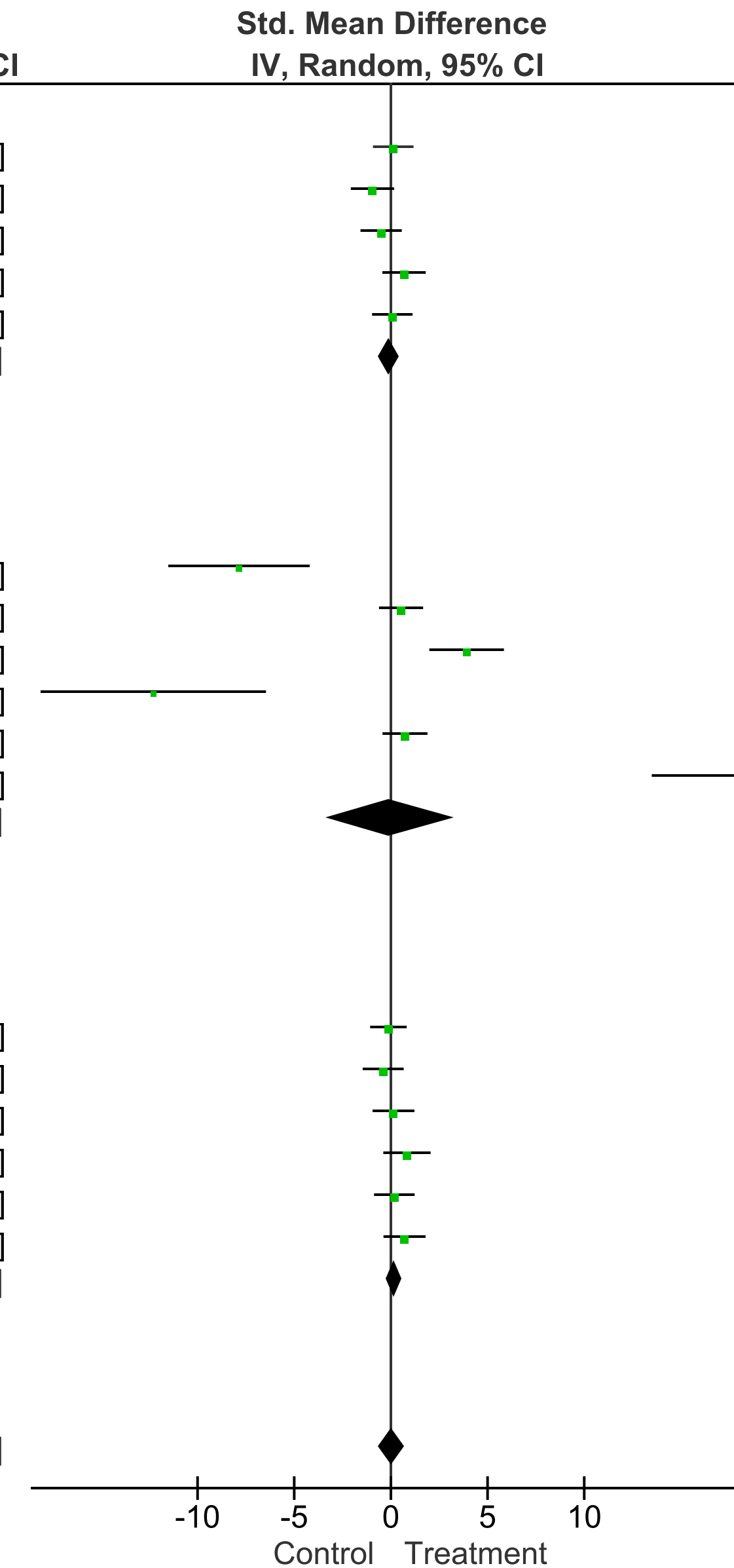

Supplementary figure 7. Meta-analysis based on the studies included in the meta-analysis of all analgesic treatments subgrouped by behaviors suppressed by pain modality. Forest plot summarizing SMDs (95% CI) for all analgesic treatments evaluated using behavioral endpoints suppressed by pain.

Study or Subgroup	Treatment Mean	Treatment SD	Treatment Total	Control Mean	Control SD	Control Total	Weight	Std. Mean Difference IV, Random, 95% CI
**2.1.1 Opioids**								
BUP0.001 (Matsumya L. C, et al. 2012) MGS	0.37	0.68	16	0.48	7.68	4	0.6%	-0.03 [-1.13, 1.06]
BUP0.009 (Jirkof, P. et al. 2019) MGS	4.8	19.2	8	2.06	19.04	4	0.5%	0.13 [-1.07, 1.33]
BUP0.009 (Jirkof, P. et al. 2019) MGS	3.85	15.4	8	1.28	12.4	4	0.5%	0.16 [-1.04, 1.37]
BUP0.01 (Matsumya L. C, et al. 2012) MGS	0.22	0.44	16	0.48	7.68	4	0.6%	-0.08 [-1.17, 1.02]
BUP0.01 (Wright-Williams, S. L. 2013) VS	0.18	2.88	16	0.48	7.68	4	0.6%	-0.07 [-1.17, 1.03]
BUP 0.024 (Faller, K.M.E, 2015)	3.17	1.48	13	1.39	0.58	13	0.6%	1.53 [0.64, 2.43]
BUP0.05 (Matsumya L. C, et al. 2012) MGS	1	0.79	16	0.48	7.68	4	0.6%	0.15 [-0.94, 1.25]
BUP0.05 (Wright-Williams, S. L. 2013) VS	2	2.72	8	4.97	2.26	8	0.6%	-1.12 [-2.20, -0.05]
BUP0.1 (Cho, C. et al. 2019) MGS	0.13	1.04	8	0.38	3.04	8	0.6%	-0.10 [-1.08, 0.88]
BUP0.1 (Cho, C. et al. 2019) MGS	0.18	1.44	8	0.42	3.36	8	0.6%	-0.09 [-1.07, 0.89]
BUP0.1 (Cho, C. et al. 2019) MGS	0.04	0.32	8	0.25	2	8	0.6%	-0.14 [-1.12, 0.84]
BUP0.1 (Cho C. et al. 2019) MGS	0.07	0.56	8	0.28	2.24	8	0.6%	-0.12 [-1.10, 0.86]
BUP0.1 (Furumoto, K. et al. 2021) NEST	3.02	1.33	8	10.3	26.3	8	0.6%	-0.37 [-1.36, 0.62]
BUP0.1 (Jirkof, P. et al. 2015) BR	0.37	0.76	8	0.88	0.84	8	0.6%	-0.60 [-1.61, 0.41]
BUP0.1 (Jirkof, P. et al. 2015) VS	0.11	1.76	16	0.48	7.68	4	0.6%	-0.10 [-1.20, 1.00]
BUP0.1 (Matsumya L. C, et al. 2012) MGS	4.31	1.35	8	3.05	1.39	8	0.6%	0.87 [-0.17, 1.91]
BUP0.1(Oliver V; Thurston S; Logfren J. 2018) VF	13.76	29.75	6	63	199	6	0.6%	-0.32 [-1.46, 0.82]
BUP0.1-0.15(MolinaCimadevila M. J. et al. 2014) HT	35.2	36.25	6	63	199	6	0.6%	-0.18 [-1.31, 0.96]
BUP0.1-0.3(MolinaCimadevila, M. J. et al. 2014) HT	1.3	0.47	7	3.17	1.61	7	0.5%	-1.48 [-2.71, -0.25]
BUP0.1 HCl (Kendal, L. V. et al. 2016) NEST	69.3	41.5	7	62.9	46.8	7	0.6%	0.14 [-0.91, 1.18]
BUP0.1 HCl (Kendal, L. V. et al. 2016) RW	21.1	26.1	7	43.2	33.2	7	0.6%	-0.69 [-1.78, 0.40]
BUP0.1 HCl (Kendal, L. V. et al. 2016) VS	1.25	0.5	7	1	0.7	7	0.6%	0.38 [-0.68, 1.45]
BUP0.2 (Adamson, T. W. et al. 2010) RW	0.5	0.87	7	0.15	0.13	7	0.6%	0.53 [-0.55, 1.60]
BUP0.2 (Adamson, T. W. et al. 2010) VS	1.71	1.08	7	1.15	0.37	7	0.6%	0.65 [-0.44, 1.73]
BUP0.2 (Clark, M. D. et al. 2004) RW	46.71	32.82	10	34.6	10.6	6	0.6%	0.42 [-0.60, 1.45]
BUP0.2 (Clark, M. D. et al. 2004) VS	46.71	32.82	10	34.6	10.6	6	0.6%	0.42 [-0.60, 1.45]
BUP0.2 (Reddyjarugu, B. et al. 2015) HT	4.76	4.35	10	2.97	1.66	9	0.6%	0.51 [-0.41, 1.43]
BUP0.2 (Reddyjarugu, B. et al. 2015) VF	2.83	0.91	10	1.1	3.12	9	0.6%	0.74 [-0.20, 1.68]
BUP0.3 (Kendal, L. V. et al. 2016) NEST	94.1	8.6	6	62.8	46.8	7	0.5%	0.83 [-0.32, 1.99]
BUP0.6 (Kendal, L. V. et al. 2016) RW	9.5	16.1	6	43.2	33.2	7	0.5%	-1.17 [-2.39, 0.05]
BUP0.6 (Kendal, L. V. et al. 2016) VS	1.7	0.3	6	1	0.7	7	0.5%	1.17 [-0.04, 2.39]
BUP1.67x10³(Lu, F. et al. 2022) VF	5.35	2	12	6.26	3.07	8	0.6%	-0.35 [-1.26, 0.55]
BUP2 (Goecke, J. C. et al. 2005) VS	0.59	0.16	7	0.18	0.09	6	0.4%	2.87 [1.16, 4.58]
BUP2 (Goecke, J. C. et al. 2005) VS	0.38	0.27	6	0.18	0.09	6	0.5%	0.92 [-0.30, 2.13]
BUP-SR1 (Navarro K, et al. 2020) HT	6.45	3.76	12	3.22	5.29	8	0.6%	0.70 [-0.23, 1.63]
BUP-SR1 (Navarro K, et al. 2020) VF	3.99	0.08	11	3.99	0.12	11	0.7%	0.00 [-0.84, 0.84]
BUP-SR2.2 (Jirkof, P. et al. 2015) BR	1.32	1.95	8	0.88	0.84	8	0.6%	0.28 [-0.71, 1.26]
BUP-SR2.2 (Jirkof, P. et al. 2015) VS	4.08	2.57	8	10.3	26.3	8	0.6%	-0.31 [-1.30, 0.67]
BUP-XR3.25 (Navarro K, et al. 2020) HT	9.07	6.72	12	3.22	5.29	8	0.6%	0.90 [-0.04, 1.85]
BUP-XR3.25 (Navarro K, et al. 2020) VF	5.45	2.84	12	6.26	3.07	8	0.6%	-0.26 [-1.16, 0.63]
BUP-XR6.25 (Navarro K, et al. 2020) HT	8.47	3.76	12	3.22	5.29	8	0.6%	1.14 [0.16, 2.12]
BUP-XR6.25 (Navarro K, et al. 2020) VF	6.29	2.28	12	6.26	3.07	8	0.6%	0.01 [-0.88, 0.91]
MOR1 (Clark, J. D. et al. 2007) VF	2.47	0.72	5	0.22	0.26	5	0.3%	3.75 [1.30, 6.20]
MOR1 (Dallazen, J. L et al. 2022) HT	12.27	3.74	6	4.48	1.35	6	0.4%	2.56 [0.87, 4.24]
MOR1 (Dallazen, J. L et al. 2022) SP	6.01	16.9	6	6.01	16.04	6	0.6%	0.00 [-1.13, 1.13]
MOR1 (Dallazen, J. L et al. 2022) VF	0.97	0.28	8	0.04	0.16	8	0.4%	3.86 [2.03, 5.68]
MOR10 (Clark, J. D. et al. 2007) VF	0.66	0.18	5	0.22	0.26	5	0.4%	1.78 [0.18, 3.37]
MOR10 (Minville, V. et al. 2008) HT	11.95	0.88	10	5.95	2.88	10	0.5%	2.70 [1.42, 3.98]
MOR10 (Minville, V. et al. 2008) VF	8	0.91	10	1.03	1.83	10	0.4%	4.62 [2.80, 6.44]
MOR10 (Pogatzki, E. M. & Raja, S. N. 2003) HT	13.27	7.83	15	1.89	1.13	9	0.6%	1.75 [0.76, 2.74]
MOR10 (Pogatzki, E. M & Raja, S. N. 2003) VF	7.81	0.001	15	2.16	7.35	15	0.7%	1.06 [0.29, 1.83]
MOR10 (Tonello, R. et al. 2020) SP	0.14	0.29	6	7.63	2.94	6	0.3%	-3.31 [-5.28, -1.34]
MOR10 (Tonello, R. et al. 2020) VF	7.99	0.49	6	2.7	0.91	6	0.1%	6.68 [3.23, 10.13]
MOR10* (Wei, S. et al. 2021) VF	5.52	0.95	8	0.32	0.35	8	0.2%	6.87 [3.96, 9.78]
MOR3 (Minville, V. et al. 2008) HT	11.96	1.06	10	5.95	2.88	10	0.5%	2.65 [1.38, 3.92]
MOR3 (Minville, V. et al. 2008) VF	3.98	6.65	10	1.03	1.83	10	0.6%	0.58 [-0.32, 1.48]
MOR3 (Pogatzki, E. M. & Raja, S. N. 2003) HT	6.05	1.3	6	2.7	0.91	6	0.4%	2.76 [1.00, 4.51]
MOR3 (Tonello, R. et al. 2020) SP	6.33	3.42	15	1.89	1.13	9	0.6%	1.52 [0.57, 2.48]
MOR3 (Tonello, R. et al. 2020) VF	2.62	5.68	6	7.63	2.94	6	0.5%	-1.02 [-2.26, 0.21]
MOR3* (Wei, S. et al. 2021) VF	2.79	0.44	8	0.32	0.35	8	0.2%	5.87 [3.33, 8.41]
MOR5* (Wei, S. et al. 2021) VF	3.49	1.52	8	0.32	0.35	8	0.5%	2.72 [1.26, 4.18]
OXY3 (Mueller, A. et al. 2019) VF	104.67	27.35	10	34.6	10.6	6	0.4%	2.90 [1.36, 4.44]
OXYM-LE 2 (Clark, M. D. et al. 2004) VS	1.43	0.24	6	1.19	0.13	5	0.5%	1.10 [-0.22, 2.42]
TRA0.1 (Jirkof, P. et al. 2019) MGS	3.2	12.8	8	3.49	15.2	4	0.5%	-0.02 [-1.22, 1.18]
TRA0.1 (Jirkof, P. et al. 2019) MGS	2.93	11.72	8	2.21	14.72	4	0.5%	0.05 [-1.15, 1.25]
TRA1 (Jirkof, P. et al. 2019) MGS	3.66	14.64	8	3.22	10.08	4	0.5%	0.03 [-1.17, 1.23]
TRA1 (Jirkof, P. et al. 2019) MGS	3	12	8	3.18	19.36	4	0.5%	-0.01 [-1.21, 1.19]
TRA100 (Romero-Alejo, E. et al. 2016) VF	2.13	3.42	8	3.64	6.05	8	0.6%	-0.29 [-1.28, 0.70]
TRA25 (Evangelista-Vaz, R. et al. 2018) BR	214	383.82	8	191	156.41	8	0.6%	0.07 [-0.91, 1.05]
TRA25 (Evangelista-Vaz, R. et al. 2018) VS	0.7	0.66	10	1.65	0.79	10	0.6%	-1.25 [-2.23, -0.27]
TRA80 (Wolfe, A. M. et al. 2015) VS	1.14	0.15	10	0.36	0.18	10	0.4%	4.51 [2.72, 6.30]
**Subtotal (95% CI)**			**644**			**511**	**37.9%**	**0.65 [0.37, 0.92]**
Heterogeneity: Tau² = 1.05; Chi² = 295.87, df = 70 (P < 0.00001); I² = 76%								
Test for overall effect: Z = 4.54 (P < 0.00001)								
**2.1.2 NSAIDS**								
CAR10 (Cho, C. et al. 2019) MGS	8	0.38	7	8	0.13	7	0.6%	0.00 [-1.05, 1.05]
CAR10 (Cho, C. et al. 2019) MGS	8	0.25	7	8	0.37	7	0.6%	0.00 [-1.05, 1.05]
CAR10 (Cho, C. et al. 2019) MGS	248.38	110.34	8	191.93	136.44	8	0.6%	0.43 [-0.56, 1.43]
CAR10 (Cho, C. et al. 2019) MGS	35	41	7	10.41	5.06	8	0.6%	0.82 [-0.25, 1.89]
CAR 10 (Matsumya L. C, et al. 2012) MGS	0.52	0.2	16	0.48	7.68	4	0.6%	0.01 [-1.08, 1.11]
CAR 15 (Matsumya L. C, et al. 2012) MGS	0.55	0.36	16	0.48	7.68	4	0.6%	0.02 [-1.07, 1.12]
CAR20 (Matsumya L. C, et al. 2012) MGS	0.04	0.23	16	0.48	7.68	4	0.6%	-0.13 [-1.23, 0.96]
CAR25 (Cho, C. et al. 2019) MGS	0.18	1.44	8	0.42	3.36	8	0.6%	-0.09 [-1.07, 0.89]
CAR25 (Cho, C. et al. 2019) MGS	0.17	1.36	8	0.28	2.24	8	0.6%	-0.06 [-1.04, 0.92]
CAR25 (Cho, C. et al. 2019) MGS	0.24	1.92	8	0.38	3.04	8	0.6%	-0.05 [-1.03, 0.93]
CAR25 (Cho, C. et al. 2019) MGS	0.01	0.001	8	0.25	2	8	0.6%	-0.16 [-1.14, 0.82]
CAR25 (Matsumya L. C, et al. 2012) MGS	0.07	1.12	16	0.48	7.68	4	0.6%	-0.12 [-1.22, 0.98]
CAR30 (Oliver V; Thurston S; Logfren J. 2018) VF	0.11	0.88	8	0.38	3.04	8	0.6%	-0.11 [-1.10, 0.87]
CAR5 (Adamson, T. W. et al. 2010) RW	0.09	0.72	8	0.28	2.24	8	0.6%	-0.11 [-1.09, 0.87]
CAR5 (Adamson, T. W. et al. 2010) VS	0.06	0.48	8	0.25	2	8	0.6%	-0.12 [-1.10, 0.86]
CAR5 (Jirkof, P. et al. 2010) BR	0.06	0.48	8	0.25	2	8	0.6%	-0.12 [-1.10, 0.86]
CAR5 (Jirkof, P. et al. 2012) VS	0.15	2.4	16	0.48	7.68	4	0.6%	-0.08 [-1.18, 1.01]
CAR5 (Jirkof, P. et al. 2013) NEST	3.9	1.63	8	3.05	1.39	8	0.6%	0.53 [-0.47, 1.53]
CAR 5 (Matsumya L. C, et al. 2012) MGS	0.43	0.52	16	16	0.48	4	0.0%	-29.04 [-39.14, -18.94]
CAR50 (Jirkof, P. et al. 2013) NEST	0.68	1.27	8	0.1	0.002	8	0.6%	0.61 [-0.40, 1.62]
CEL1 (Muratani T. et al. 2005) VF	1.43	0.24	6	1.19	0.13	5	0.5%	1.10 [-0.22, 2.42]
CEL1 (Muratani T. et al. 2005) VF	5.65	2.34	6	5.89	3.24	6	0.6%	-0.08 [-1.21, 1.05]
CEL10 (Muratani T. et al. 2005) VF	5.96	1.62	6	5.89	3.24	6	0.6%	0.03 [-1.11, 1.16]
CEL10 (Muratani T. et al. 2005) VF	6.2	3	6	8.58	3.9	6	0.5%	-0.63 [-1.80, 0.54]
DEX100 (Romero-Alejo, E. et al. 2016) VF	0.9	0.11	10	0.36	0.18	10	0.4%	3.47 [1.98, 4.95]
DIC0.25 (Lu, F. et al. 2022) VF	3.84	0.69	11	3.99	0.12	11	0.7%	-0.29 [-1.13, 0.55]
DIC0.25 (Lu, F. et al. 2022) VF	1.29	0.8	11	1.57	1.56	11	0.7%	-0.22 [-1.06, 0.62]
FIR10 (Reddyjarugu, B. et al. 2015) HT	5.59	4.61	9	2.97	1.66	9	0.6%	0.72 [-0.24, 1.68]
FIR10 (Reddyjarugu, B. et al. 2015) VF	2.41	1.39	9	1.1	3.12	9	0.6%	0.52 [-0.43, 1.46]
FIR20 (Reddyjarugu, B. et al. 2015) HT	2.24	0.47	10	1.1	3.12	9	0.6%	0.50 [-0.42, 1.42]
FIR20 (Reddyjarugu, B. et al. 2015) VF	7.14	3.91	10	2.97	1.66	9	0.6%	1.30 [0.29, 2.31]
FLU2.5 (Goecke, J. C. et al. 2005) VS	0.23	0.13	7	0.18	0.09	6	0.6%	0.41 [-0.70, 1.52]
FLU2.5 (Goecke, J. C. et al. 2005) VS	0.27	0.15	6	0.18	0.09	6	0.5%	0.67 [-0.51, 1.85]
IBU100 (Saad, S. S. T. et al. 2016) VF	2.58	0.97	9	0.74	0.46	8	0.5%	2.25 [0.97, 3.54]
IBU300 (Saad, S. S. T. et al. 2016) VF	2.98	2.29	9	0.74	0.46	8	0.6%	1.25 [0.18, 2.32]
INDO1 (Brusco, I. et al. 2017) VF	3.23	0.79	6	1.44	1.27	6	0.5%	1.56 [0.20, 2.93]
INDO10 (Brusco, I. et al. 2017) SP	4.06	0.48	6	1.44	1.27	6	0.4%	2.52 [0.85, 4.19]
INDO10 (Brusco, I. et al. 2017) SP	5.5	21.9	6	8.94	6.89	6	0.6%	-0.20 [-1.33, 0.94]
INDO10 (Brusco, I. et al. 2017) VF	0.93	12.47	6	1.98	0.49	6	0.6%	-0.11 [-1.24, 1.02]
INDO10 (Brusco, I. et al. 2017) VF	4.3	0.48	6	1.77	1.07	6	0.4%	2.82 [1.04, 4.60]
INDO3 (Brusco, I. et al. 2017) VF	4.23	0.59	6	1.44	1.27	6	0.4%	2.60 [0.90, 4.30]
KET20 (Matsumya L. C, et al. 2012) MGS	0.19	3.04	16	0.48	7.68	4	0.6%	-0.07 [-1.16, 1.03]
KET50 (Minville, V. et al. 2008) HT	11.91	1.24	10	5.95	2.88	10	0.5%	2.57 [1.33, 3.82]
KET50 (Minville, V. et al. 2008) VF	3.99	7.34	10	1.03	1.83	10	0.6%	0.53 [-0.37, 1.43]
KETO 1 (Matsumya L. C, et al. 2012) MGS	0.53	0.56	16	0.48	7.68	4	0.6%	0.02 [-1.08, 1.11]
KETO 10 (Matsumya L. C, et al. 2012) MGS	0.64	0.6	16	0.48	7.68	4	0.6%	0.05 [-1.05, 1.14]
KETO 15 (Matsumya L. C, et al. 2012) MGS	0.18	0.44	16	0.48	7.68	4	0.6%	-0.09 [-1.19, 1.01]
KETO 5 (Matsumya L. C, et al. 2012) MGS	0.43	0.2	16	0.48	7.68	4	0.6%	-0.02 [-1.11, 1.08]
MEL10 (Roughan, V. J. et al. 2009) VS	6.37	4.48	8	3.76	5.13	9	0.6%	0.51 [-0.46, 1.48]
MEL10 (Roughan, V. J. et al. 2016) VS	24.27	26.33	8	10.97	27.26	8	0.6%	0.47 [-0.53, 1.47]
MEL2(Cho, C. et al. 2019) MGS	0.11	0.88	8	0.42	3.36	8	0.6%	-0.12 [-1.10, 0.86]
MEL2 (Cho, C. et al. 2019) MGS	0.14	1.12	8	0.38	3.04	8	0.6%	-0.10 [-1.08, 0.88]
MEL2 (Cho, C. et al. 2019) MGS	0.08	0.64	8	0.38	3.04	8	0.6%	-0.13 [-1.11, 0.85]
MEL2 (Cho, C. et al. 2019) MGS	0.01	0.04	8	0.25	2	8	0.6%	-0.16 [-1.14, 0.82]
MEL20 (Leach, M. C. et al. 2012) MGS	2.35	14.1	6	3.86	23.16	6	0.6%	-0.07 [-1.20, 1.06]
MEL20 (Roughan, V. J. et al. 2009) VS	16.95	14.11	8	10.97	27.26	8	0.6%	0.26 [-0.72, 1.25]
MEL20 (Wright-Williams, S. L. 2009) VS	8.5	9.8	8	17.1	30.8	8	0.6%	-0.36 [-1.35, 0.63]
MEL5 (Cho, C. et al. 2019) MGS	0.2	1.6	8	0.42	3.36	8	0.6%	-0.08 [-1.06, 0.90]
MEL5 (Cho, C. et al. 2019) MGS	0.08	0.64	8	0.38	3.04	8	0.6%	-0.13 [-1.11, 0.85]
MEL5 (Cho, C. et al. 2019) MGS	0.06	0.48	8	0.28	2.24	8	0.6%	-0.13 [-1.11, 0.85]
MEL5 (Cho, C. et al. 2019) MGS	0.12	0.96	8	0.25	2	8	0.6%	-0.08 [-1.06, 0.90]
MEL5 (Roughan, V. J. et al. 2009) VS	50.54	20.7	8	10.97	27.26	8	0.5%	1.55 [0.39, 2.70]
**Subtotal (95% CI)**			**576**			**440**	**35.0%**	**0.33 [0.12, 0.53]**
Heterogeneity: Tau² = 0.37; Chi² = 137.38, df = 61 (P < 0.00001); I² = 56%								
Test for overall effect: Z = 3.10 (P = 0.002)								
**2.1.3 Acetaminophen**								
ACE100 (Cabañero & Maldonado. 2021) HT	4.65	1.35	10	3.58	1.69	10	0.6%	0.67 [-0.24, 1.58]
ACE100 (Cabañero & Maldonado. 2021) VF	0.58	0.21	10	0.38	0.14	10	0.6%	1.07 [0.12, 2.03]
ACE 100 (Matsumya L. C, et al. 2012) MGS	0.57	0.56	16	0.48	7.68	4	0.6%	0.03 [-1.07, 1.12]
ACE120 (Zhu, Q. et al. 2016) VF	33.28	18.22	10	100	314	10	0.6%	-0.29 [-1.17, 0.59]
ACE200 (Cabañero & Maldonado. 2021) HT	6.3	2.94	10	3.58	1.69	10	0.6%	1.09 [0.13, 2.04]
ACE200 (Cabañero & Maldonado. 2021) VF	0.89	0.24	10	0.38	0.14	10	0.5%	2.49 [1.26, 3.71]
ACE200 (Durst, M. S. et al. 2021) BR	90.36	180.6	8	68.67	156.75	8	0.6%	0.12 [-0.86, 1.10]
ACE200 (Durst, M. S. et al. 2021) BR	126.06	149.48	10	63.03	203.14	10	0.6%	0.34 [-0.55, 1.22]
ACE200 (Durst, M. S. et al. 2021) MGS	0.63	6.3	10	0.73	6.3	10	0.6%	-0.02 [-0.89, 0.86]
ACE200 (Durst, M. S. et al. 2021) MGS	0.54	4.32	8	0.62	4.4	8	0.6%	-0.02 [-1.00, 0.96]
ACE200 (Durst, M. S. et al. 2021) NEST	0.52	1.55	10	0.77	2.15	10	0.6%	-0.13 [-1.01, 0.75]
ACE200 (Durst, M. S. et al. 2021) NEST	0.5	1.5	8	1.11	1.38	8	0.6%	-0.40 [-1.39, 0.59]
ACE240 (Zhu, Q. et al. 2016) VF	43.21	33.36	10	100	314	10	0.6%	-0.24 [-1.12, 0.64]
ACE30 (Zhu, Q. et al. 2016) VF	21.6	21.23	10	100	314	10	0.6%	-0.34 [-1.22, 0.55]
ACE300 (Cabañero & Maldonado. 2021) HT	6.02	2.29	10	3.58	1.69	10	0.6%	1.16 [0.20, 2.13]
ACE300 (Cabañero & Maldonado. 2021) VF	1.03	0.28	10	0.38	0.14	10	0.5%	2.81 [1.50, 4.12]
ACE 300 (Matsumya L. C, et al. 2012) MGS	0.37	0.72	16	0.48	7.68	4	0.6%	-0.03 [-1.13, 1.06]
ACE360 (Zhu, Q. et al. 2016) VF	60.14	29.34	10	100	314	10	0.6%	-0.17 [-1.05, 0.71]
ACE400 (Cabañero & Maldonado. 2021) HT	6.49	2.94	10	3.58	1.69	10	0.6%	1.16 [0.20, 2.13]
ACE400 (Cabañero & Maldonado. 2021) VF	1.28	0.21	10	0.38	0.14	10	0.3%	4.83 [2.94, 6.72]
ACE 450 (Matsumya L. C, et al. 2012) MGS	0.32	0.68	16	0.48	7.68	4	0.6%	-0.05 [-1.14, 1.05]
ACE50 (Cabañero & Maldonado. 2021) HT	3.2	2.01	10	3.58	1.69	10	0.6%	-0.20 [-1.08, 0.68]
ACE50 (Cabañero & Maldonado. 2021) VF	0.52	0.24	10	0.38	0.14	10	0.6%	0.68 [-0.23, 1.59]
ACE60 (Zhu, Q. et al. 2016) VF	28.32	37.41	10	100	314	10	0.6%	-0.31 [-1.19, 0.58]
**Subtotal (95% CI)**			**252**			**216**	**14.4%**	**0.47 [0.11, 0.83]**
Heterogeneity: Tau² = 0.55; Chi² = 74.92, df = 23 (P < 0.00001); I² = 69%								
Test for overall effect: Z = 2.56 (P = 0.01)								
**2.1.4 Gabapentinoids**								
GABA20 (Yang J. et al. 2019) VF	0.28	0.15	10	0.05	0.03	9	0.6%	1.98 [0.83, 3.12]
GABA50 (Yang J. et al. 2019) VF	0.53	0.08	6	0.05	0.03	5	0.1%	6.98 [3.15, 10.81]
PRE30 (Park, J. H et al. 2020) VF	6.5	4	8	8.59	2.12	8	0.6%	-0.62 [-1.63, 0.39]
**Subtotal (95% CI)**			**24**			**22**	**1.3%**	**2.16 [-0.72, 5.04]**
Heterogeneity: Tau² = 5.34; Chi² = 21.62, df = 2 (P < 0.0001); I² = 91%								
Test for overall effect: Z = 1.47 (P = 0.14)								
**2.1.5 LA**								
BUV5 (Leach, M. C. et al. 2012) MGS	1.99	11.94	6	3.86	23.16	6	0.6%	-0.09 [-1.23, 1.04]
ROP5% (Yamada, T. et al. 2016) HT	18.11	7.21	9	10.54	3.22	9	0.6%	1.29 [0.25, 2.33]
ROP5% (Yamada, T. et al. 2016) HT	1.97	0.42	9	0.16	1.15	9	0.5%	1.99 [0.81, 3.17]
ROP5% (Yamada, T. et al. 2016) VF	1.88	1.95	9	0.1	0.76	9	0.6%	1.15 [0.13, 2.16]
ROP5% (Yamada, T. et al. 2016) VF	20.4	2.88	9	9.77	3.61	9	0.5%	3.10 [1.63, 4.57]
**Subtotal (95% CI)**			**42**			**42**	**2.7%**	**1.42 [0.49, 2.35]**
Heterogeneity: Tau² = 0.77; Chi² = 12.96, df = 4 (P = 0.01); I² = 69%								
Test for overall effect: Z = 3.00 (P = 0.003)								
**2.1.6 multimodal**								
ACE+LID+BUV (Durst, M. S. 2021) BR	101.81	187.83	3	63	203.1	3	0.4%	0.16 [-1.45, 1.77]
ACE+LID+BUV (Durst, M. S. 2021) BR	101.2	153.35	3	68.7	156.8	3	0.4%	0.17 [-1.44, 1.78]
ACE+LID+BUV (Durst, M. S. 2021) MGS	0.58	2.18	3	0.59	6.3	3	0.4%	-0.00 [-1.60, 1.60]
ACE+LID+BUV (Durst, M. S. 2021) MGS	0.61	4.88	3	0.75	4.4	3	0.4%	-0.02 [-1.62, 1.58]
ACE+LID+BUV (Durst, M. S. 2021) NEST	0.7	1.27	3	1.11	1.38	3	0.4%	-0.25 [-1.87, 1.37]
ACE+LID+BUV (Durst, M. S. 2021) NEST	0.28	1.39	3	0.77	2.15	3	0.4%	-0.22 [-1.83, 1.40]
BUP+CAR (Adamsom, T, W) 2010 RW	0.17	0.18	2	0.15	0.13	2	0.3%	0.07 [-1.93, 2.08]
BUP+CAR (Adamsom, T. W. 2010) VS	1.4	0.55	2	1.15	0.37	2	0.2%	0.30 [-2.31, 2.92]
BUP+MEL+BUV (Furumoto, K. 2021) NEST	1.3	0.47	3	3.17	1.61	3	0.3%	-1.26 [-3.27, 0.75]
LID+BUV (Durst, M. S. 2021) BR	103.03	203.14	3	63	203.1	3	0.4%	0.16 [-1.45, 1.77]
LID+BUV (Durst, M. S. 2021) BR	26.5	95.43	3	68.7	156.8	3	0.4%	-0.26 [-1.88, 1.36]
LID+BUV (Durst, M. S. 2021) MGS	0.41	4.1	3	0.58	6.3	3	0.4%	-0.03 [-1.63, 1.57]
LID+BUV (Durst, M. S. 2021) MGS	0.26	2.08	3	0.35	4.4	3	0.4%	-0.02 [-1.62, 1.58]
LID+BUV (Durst, M. S. 2021) NEST	0.4	1.41	3	1.11	1.38	3	0.4%	-0.41 [-2.06, 1.24]
LID+BUV (Durst, M. S. 2021) NEST	0.89	2.187	3	0.77	2.15	3	0.4%	0.04 [-1.56, 1.65]
MEL+BUP (Molina-Cimadevila, M. J. 2014) HT	35.36	37.48	2	63	199	2	0.3%	-0.11 [-2.17, 1.95]
TRA+ACE (Jirkof, P. 2018) MGS	2.3	18.4	8	3.1	24.8	8	0.6%	-0.03 [-1.01, 0.95]
TRA+ACE (Jirkof, P. 2018) NEST	2.97	1.32	8	2.57	0.84	8	0.6%	0.34 [-0.65, 1.33]
TRA+ACE (Jirkof, P. 2018) VS	2.35	1.89	8	3.86	3.16	8	0.6%	-0.55 [-1.55, 0.46]
TRA+ACE (Jirkof, P. 2018) VS	3.36	2.17	8	4.54	2	8	0.6%	-0.53 [-1.54, 0.47]
TRA+DEXK (Romero-Alejo, E. 2016) VF	0.82	0.14	3	0.36	0.18	3	0.2%	2.28 [-0.44, 5.01]
**Subtotal (95% CI)**			**80**			**80**	**8.8%**	**-0.10 [-0.42, 0.22]**
Heterogeneity: Tau² = 0.00; Chi² = 7.21, df = 20 (P = 1.00); I² = 0%								
Test for overall effect: Z = 0.62 (P = 0.54)								
**Total (95% CI)**			**1618**			**1311**	**100.0%**	**0.47 [0.32, 0.61]**
Heterogeneity: Tau² = 0.67; Chi² = 575.89, df = 185 (P < 0.00001); I² = 68%								
Test for overall effect: Z = 6.22 (P < 0.00001)								
Test for subgroup differences: Chi² = 18.91, df = 5 (P = 0.002), I² = 73.6%								

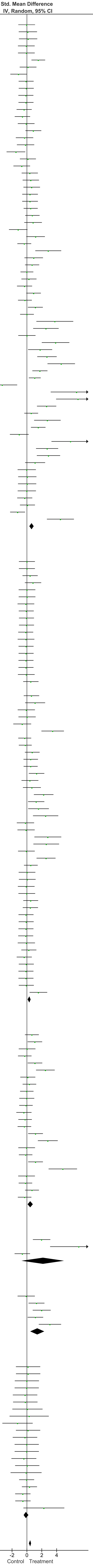

Supplementary figure 8. Meta-analysis based on the studies included in the meta-analysis of different analgesic treatments on all pain modalities. Forest plot presenting pooled SMDs (95% CI) for analgesic classes (opioids, NSAIDs, acetaminophen, gabapentinoids, local anesthetics, and multimodal therapy), considering all pain modalities.

Study or Subgroup	Experimental Mean	Experimental SD	Experimental Total	Control Mean	Control SD	Control Total	Weight	Std. Mean Difference IV, Random, 95% CI
**2.3.2 Buprenorphine**								
BUP0.009 (Jirkof, P. et al. 2019) MGS	4.8	19.2	8	2.06	19.04	4	1.5%	0.13 [-1.07, 1.33]
BUP0.009 (Jirkof, P. et al. 2019) MGS	3.85	15.4	8	1.28	12.4	4	1.5%	0.16 [-1.04, 1.37]
BUP0.01 (Wright-Williams, S. L. 2013) VS	1	0.79	8	4.97	2.26	8	1.4%	-2.22 [-3.54, -0.90]
BUP0.05 (Matsumya L. C, et al. 2012) MGS	2	2.72	8	4.97	2.26	8	1.5%	-1.12 [-2.20, -0.05]
BUP0.05 (Wright-Williams, S. L. 2013) VS	0.18	2.88	16	0.48	7.68	4	1.5%	-0.07 [-1.17, 1.03]
BUP0.1 (Cho, C. et al. 2019) MGS	0.13	1.04	8	0.38	3.04	8	1.6%	-0.10 [-1.08, 0.88]
BUP0.1 (Cho, C. et al. 2019) MGS	0.18	1.44	8	0.42	3.36	8	1.6%	-0.09 [-1.07, 0.89]
BUP0.1 (Cho, C. et al. 2019) MGS	0.04	0.32	8	0.25	2	8	1.6%	-0.14 [-1.12, 0.84]
BUP0.1 (Cho C. et al. 2019) MGS	0.07	0.56	8	0.28	2.24	8	1.6%	-0.12 [-1.10, 0.86]
BUP0.1 (Furumoto, K. et al. 2021) NEST	3.02	1.33	8	10.3	26.3	8	1.6%	-0.37 [-1.36, 0.62]
BUP0.1 (Jirkof, P. et al. 2015) BR	0.37	0.76	8	0.88	0.84	8	1.6%	-0.60 [-1.61, 0.41]
BUP0.1 (Jirkof, P. et al. 2015) VS	0.11	1.76	16	0.48	7.68	4	1.5%	-0.10 [-1.20, 1.00]
BUP0.1 (Matsumya L. C, et al. 2012) MGS	4.31	1.35	8	3.05	1.39	8	1.6%	0.87 [-0.17, 1.91]
BUP0.1(Oliver V; Thurston S; Logfren J. 2018) VF	13.76	29.75	6	63	199	6	1.5%	-0.32 [-1.46, 0.82]
BUP0.1-0.15(MolinaCimadevila M. J. et al. 2014) HT	35.2	36.25	6	63	199	6	1.5%	-0.18 [-1.31, 0.96]
BUP0.1-0.3(MolinaCimadevila, M. J. et al. 2014) HT	1.3	0.47	7	3.17	1.61	7	1.4%	-1.48 [-2.71, -0.25]
BUP0.1 HCl (Kendal, L. V. et al. 2016) NEST	69.3	41.5	7	62.9	46.8	7	1.6%	0.14 [-0.91, 1.18]
BUP0.1 HCl (Kendal, L. V. et al. 2016) RW	21.1	26.1	7	43.2	33.2	7	1.5%	-0.69 [-1.78, 0.40]
BUP0.1 HCl (Kendal, L. V. et al. 2016) VS	1.25	0.5	7	1	0.7	7	1.6%	0.38 [-0.68, 1.45]
BUP0.2 (Adamson, T. W. et al. 2010) RW	0.5	0.87	7	0.15	0.13	7	1.5%	0.53 [-0.55, 1.60]
BUP0.2 (Adamson, T. W. et al. 2010) VS	1.71	1.08	7	1.15	0.37	7	1.5%	0.65 [-0.44, 1.73]
BUP0.2 (Clark, M. D. et al. 2004) RW	46.71	32.82	10	34.6	10.6	6	1.6%	0.42 [-0.60, 1.45]
BUP0.2 (Clark, M. D. et al. 2004) VS	46.71	32.82	10	34.6	10.6	6	1.6%	0.42 [-0.60, 1.45]
BUP0.2 (Reddyjarugu, B. et al. 2015) HT	4.76	4.35	10	2.97	1.66	9	1.6%	0.51 [-0.41, 1.43]
BUP0.2 (Reddyjarugu, B. et al. 2015) VF	2.83	0.91	10	1.1	3.12	9	1.6%	0.74 [-0.20, 1.68]
BUP0.6 (Kendal, L. V. et al. 2016) NEST	94.1	8.6	6	62.8	46.8	7	1.5%	0.83 [-0.32, 1.99]
BUP0.6 (Kendal, L. V. et al. 2016) RW	9.5	16.1	6	43.2	33.2	7	1.5%	-1.17 [-2.39, 0.05]
BUP0.6 (Kendal, L. V. et al. 2016) VS	1.7	0.3	6	1	0.7	7	1.5%	1.17 [-0.04, 2.39]
BUP1.67x10³(Lu, F. et al. 2022) VF	5.35	2	12	6.26	3.07	8	1.6%	-0.35 [-1.26, 0.55]
BUP2 (Goecke, J. C. et al. 2005) VS	6.45	3.76	12	3.22	5.29	8	1.6%	0.70 [-0.23, 1.63]
BUP2 (Goecke, J. C. et al. 2005) VS	3.99	0.08	11	3.99	0.12	11	1.7%	0.00 [-0.84, 0.84]
BUP-SR1 (Navarro K, et al. 2020) HT	0.59	0.16	7	0.18	0.09	6	1.2%	2.87 [1.16, 4.58]
BUP-SR1 (Navarro K, et al. 2020) VF	0.38	0.27	6	0.18	0.09	6	1.5%	0.92 [-0.30, 2.13]
BUP-SR2.2 (Jirkof, P. et al. 2015) BR	1.32	1.95	8	0.88	0.84	8	1.6%	0.28 [-0.71, 1.26]
BUP-SR2.2 (Jirkof, P. et al. 2015) VS	4.08	2.57	8	10.3	26.3	8	1.6%	-0.31 [-1.30, 0.67]
BUP-XR3.25 (Navarro K, et al. 2020) HT	9.07	6.72	12	3.22	5.29	8	1.6%	0.90 [-0.04, 1.85]
BUP-XR3.25 (Navarro K, et al. 2020) VF	5.45	2.84	12	6.26	3.07	8	1.6%	-0.26 [-1.16, 0.63]
BUP-XR6.25 (Navarro K, et al. 2020) HT	8.47	3.76	12	3.22	5.29	8	1.6%	1.14 [0.16, 2.12]
BUP-XR6.25 (Navarro K, et al. 2020) VF	6.29	2.28	12	6.26	3.07	8	1.7%	0.01 [-0.88, 0.91]
BUP 0.024 (Faller, K.M.E, 2015)	3.17	1.48	13	1.39	0.58	13	1.7%	1.53 [0.64, 2.43]
BUP0.001 (Matsumya L. C, et al. 2012) MGS	0.37	0.68	16	0.48	7.68	4	1.5%	-0.03 [-1.13, 1.06]
BUP0.01 (Matsumya L. C, et al. 2012) MGS	0.22	0.44	16	0.48	7.68	4	1.5%	-0.08 [-1.17, 1.02]
**Subtotal (95% CI)**			**389**			**301**	**65.0%**	**0.13 [-0.10, 0.35]**
Heterogeneity: Tau² = 0.26; Chi² = 78.40, df = 41 (P = 0.0004); I² = 48%								
Test for overall effect: Z = 1.10 (P = 0.27)								
**2.3.3 Morphine**								
MOR1 (Clark, J. D. et al. 2007) VF	2.47	0.72	5	0.22	0.26	5	0.8%	3.75 [1.30, 6.20]
MOR1 (Dallazen, J. L et al. 2022) HT	12.27	3.74	6	4.48	1.35	6	1.2%	2.56 [0.87, 4.24]
MOR1 (Dallazen, J. L et al. 2022) SP	6.01	16.9	6	6.01	16.04	6	1.5%	0.00 [-1.13, 1.13]
MOR1 (Dallazen, J. L et al. 2022) VF	0.97	0.28	8	0.04	0.16	8	1.1%	3.86 [2.03, 5.68]
MOR10 (Clark, J. D. et al. 2007) VF	11.96	1.06	10	5.95	2.88	10	1.4%	2.65 [1.38, 3.92]
MOR10 (Minville, V. et al. 2008) HT	3.98	6.65	10	1.03	1.83	10	1.6%	0.58 [-0.32, 1.48]
MOR10 (Minville, V. et al. 2008) VF	6.33	3.42	15	1.89	1.13	9	1.6%	1.52 [0.57, 2.48]
MOR10 (Pogatzki, E. M. & Raja, S. N. 2003) HT	2.62	5.68	6	7.63	2.94	6	1.4%	-1.02 [-2.26, 0.21]
MOR10 (Pogatzki, E. M & Raja, S. N. 2003) VF	6.05	1.3	6	2.7	0.91	6	1.1%	2.76 [1.00, 4.51]
MOR10 (Tonello, R. et al. 2020) SP	2.79	0.44	8	0.32	0.35	8	0.8%	5.87 [3.33, 8.41]
MOR10 (Tonello, R. et al. 2020) VF	3.49	1.52	8	0.32	0.35	8	1.3%	2.72 [1.26, 4.18]
MOR10* (Wei, S. et al. 2021) VF	5.52	0.95	8	0.32	0.35	8	0.7%	6.87 [3.96, 9.78]
MOR3 (Minville, V. et al. 2008) HT	0.66	0.18	5	0.22	0.26	5	1.2%	1.78 [0.18, 3.37]
MOR3 (Minville, V. et al. 2008) VF	11.95	0.88	10	5.95	2.88	10	1.4%	2.70 [1.42, 3.98]
MOR3 (Pogatzki, E. M. & Raja, S. N. 2003) HT	8	0.91	10	1.03	1.83	10	1.1%	4.62 [2.80, 6.44]
MOR3 (Tonello, R. et al. 2020) SP	13.27	7.83	15	1.89	1.13	9	1.6%	1.75 [0.76, 2.74]
MOR3 (Tonello, R. et al. 2020) VF	7.81	0.001	15	2.16	7.35	15	1.7%	1.06 [0.29, 1.83]
MOR3* (Wei, S. et al. 2021) VF	0.14	0.29	6	7.63	2.94	6	1.0%	-3.31 [-5.28, -1.34]
MOR5* (Wei, S. et al. 2021) VF	7.99	0.49	6	2.7	0.91	6	0.5%	6.68 [3.23, 10.13]
**Subtotal (95% CI)**			**163**			**151**	**23.2%**	**2.21 [1.38, 3.04]**
Heterogeneity: Tau² = 2.66; Chi² = 117.54, df = 18 (P < 0.00001); I² = 85%								
Test for overall effect: Z = 5.23 (P < 0.00001)								
**2.3.4 Tramadol**								
TRA0.1 (Jirkof, P. et al. 2019) MGS	3.2	12.8	8	3.49	15.2	4	1.5%	-0.02 [-1.22, 1.18]
TRA0.1 (Jirkof, P. et al. 2019) MGS	2.93	11.72	8	2.21	14.72	4	1.5%	0.05 [-1.15, 1.25]
TRA1 (Jirkof, P. et al. 2019) MGS	3.66	14.64	8	3.22	10.08	4	1.5%	0.03 [-1.17, 1.23]
TRA1 (Jirkof, P. et al. 2019) MGS	3	12	8	3.18	19.36	4	1.5%	-0.01 [-1.21, 1.19]
TRA100 (Romero-Alejo, E. et al. 2016) VF	2.13	3.42	8	3.64	6.05	8	1.6%	-0.29 [-1.28, 0.70]
TRA25 (Evangelista-Vaz, R. et al. 2018) BR	214	383.82	8	191	156.41	8	1.6%	0.07 [-0.91, 1.05]
TRA25 (Evangelista-Vaz, R. et al. 2018) VS	0.7	0.66	10	1.65	0.79	10	1.6%	-1.25 [-2.23, -0.27]
TRA80 (Wolfe, A. M. et al. 2015) VS	1.14	0.15	10	0.36	0.18	10	1.1%	4.51 [2.72, 6.30]
**Subtotal (95% CI)**			**68**			**52**	**11.8%**	**0.24 [-0.62, 1.10]**
Heterogeneity: Tau² = 1.17; Chi² = 31.05, df = 7 (P < 0.0001); I² = 77%								
Test for overall effect: Z = 0.55 (P = 0.58)								
**Total (95% CI)**			**620**			**504**	**100.0%**	**0.58 [0.30, 0.87]**
Heterogeneity: Tau² = 1.10; Chi² = 299.96, df = 68 (P < 0.00001); I² = 77%								
Test for overall effect: Z = 3.97 (P < 0.0001)								
Test for subgroup differences: Chi² = 22.68, df = 2 (P < 0.0001), I² = 91.2%								

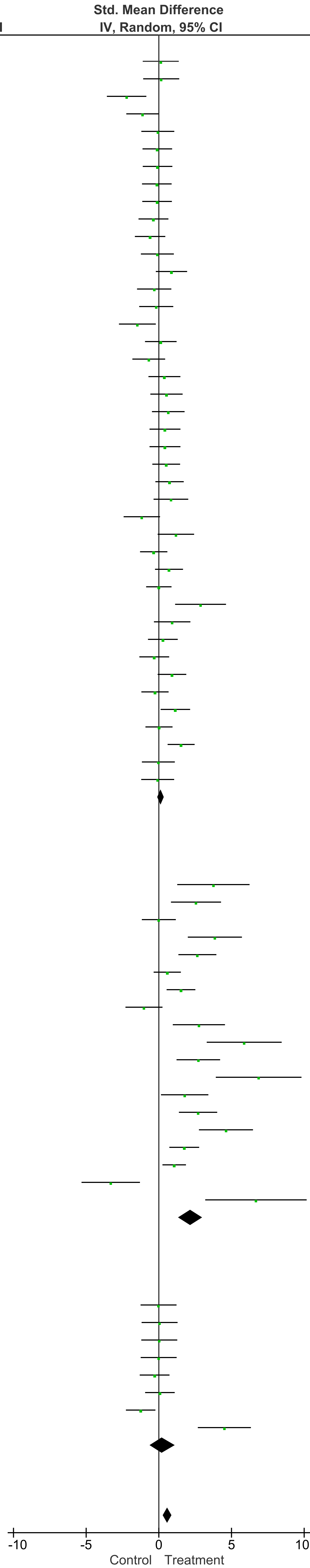

Supplementary figure 9. Meta-analysis based on the studies included in the meta-analysis of opioid analgesic treatments. Forest plot showing pooled SMDs (95% CI) for all opioid treatments (buprenorphine, morphine, oxycodone, oxymorphone, tramadol).

Study or Subgroup	Experimental Mean	Experimental SD	Experimental Total	Control Mean	Control SD	Control Total	Weight	Std. Mean Difference IV, Random, 95% CI
**2.5.1 Carprofen**								
CAR5 (Adamson, T. W. et al. 2010) RW	8	0.38	7	8	0.13	7	2.3%	0.00 [-1.05, 1.05]
CAR5 (Adamson, T. W. et al. 2010) VS	8	0.25	7	8	0.37	7	2.3%	0.00 [-1.05, 1.05]
CAR5 (Jirkof, P. et al. 2012) VS	248.38	110.34	8	191.93	136.44	8	2.5%	0.43 [-0.56, 1.43]
CAR5 (Jirkof, P. et al. 2010) BR	35	41	7	10.41	5.06	8	2.3%	0.82 [-0.25, 1.89]
CAR5 (Jirkof, P. et al. 2013) NEST	0.04	0.23	8	0.1	0.002	8	2.5%	-0.35 [-1.34, 0.64]
CAR10 (Cho, C. et al. 2019) MGS	0.18	1.44	8	0.42	3.36	8	2.5%	-0.09 [-1.07, 0.89]
CAR10 (Cho, C. et al. 2019) MGS	0.17	1.36	8	0.28	2.24	8	2.5%	-0.06 [-1.04, 0.92]
CAR10 (Cho, C. et al. 2019) MGS	0.24	1.92	8	0.38	3.04	8	2.5%	-0.05 [-1.03, 0.93]
CAR10 (Cho, C. et al. 2019) MGS	0.01	0.001	8	0.25	2	8	2.5%	-0.16 [-1.14, 0.82]
CAR20 (Matsumya L. C, et al. 2012) MGS	0.07	1.12	16	0.48	7.68	4	2.2%	-0.12 [-1.22, 0.98]
CAR25 (Cho, C. et al. 2019) MGS	0.11	0.88	8	0.38	3.04	8	2.5%	-0.11 [-1.10, 0.87]
CAR25 (Cho, C. et al. 2019) MGS	0.09	0.72	8	0.28	2.24	8	2.5%	-0.11 [-1.09, 0.87]
CAR25 (Cho, C. et al. 2019) MGS	0.06	0.48	8	0.25	2	8	2.5%	-0.12 [-1.10, 0.86]
CAR25 (Cho, C. et al. 2019) MGS	0.06	0.48	8	0.25	2	8	2.5%	-0.12 [-1.10, 0.86]
CAR25 (Matsumya L. C, et al. 2012) MGS	0.15	2.4	16	0.48	7.68	4	2.2%	-0.08 [-1.18, 1.01]
CAR30 (Oliver V; Thurston S; Logfren J. 2018) VF	3.9	1.63	8	3.05	1.39	8	2.5%	0.53 [-0.47, 1.53]
CAR50 (Jirkof, P. et al. 2013) NEST	0.68	1.27	8	0.1	0.002	8	2.4%	0.61 [-0.40, 1.62]
**Subtotal (95% CI)**			**149**			**126**	**41.6%**	**0.05 [-0.19, 0.30]**
Heterogeneity: Tau² = 0.00; Chi² = 6.21, df = 16 (P = 0.99); I² = 0%								
Test for overall effect: Z = 0.43 (P = 0.67)								
**2.5.2 Celecoxib**								
CEL1 (Muratani T. et al. 2005) VF	1.43	0.24	6	1.19	0.13	5	1.7%	1.10 [-0.22, 2.42]
CEL1 (Muratani T. et al. 2005) VF	5.65	2.34	6	5.89	3.24	6	2.1%	-0.08 [-1.21, 1.05]
CEL10 (Muratani T. et al. 2005) VF	5.96	1.62	6	5.89	3.24	6	2.1%	0.03 [-1.11, 1.16]
CEL10 (Muratani T. et al. 2005) VF	6.2	3	6	8.58	3.9	6	2.0%	-0.63 [-1.80, 0.54]
**Subtotal (95% CI)**			**24**			**23**	**7.9%**	**0.06 [-0.61, 0.72]**
Heterogeneity: Tau² = 0.10; Chi² = 3.80, df = 3 (P = 0.28); I² = 21%								
Test for overall effect: Z = 0.17 (P = 0.87)								
**2.5.3 Indometacin**								
INDO1 (Brusco, I. et al. 2017) VF	3.23	0.79	6	1.44	1.27	6	1.6%	1.56 [0.20, 2.93]
INDO3 (Brusco, I. et al. 2017) VF	4.06	0.48	6	1.44	1.27	6	1.2%	2.52 [0.85, 4.19]
INDO10 (Brusco, I. et al. 2017) SP	5.5	21.9	6	8.94	6.89	6	2.1%	-0.20 [-1.33, 0.94]
INDO10 (Brusco, I. et al. 2017) SP	0.93	12.47	6	1.98	0.49	6	2.1%	-0.11 [-1.24, 1.02]
INDO10 (Brusco, I. et al. 2017) VF	4.3	0.48	6	1.77	1.07	6	1.0%	2.82 [1.04, 4.60]
INDO10 (Brusco, I. et al. 2017) VF	4.23	0.59	6	1.44	1.27	6	1.1%	2.60 [0.90, 4.30]
**Subtotal (95% CI)**			**36**			**36**	**9.1%**	**1.41 [0.27, 2.55]**
Heterogeneity: Tau² = 1.46; Chi² = 19.13, df = 5 (P = 0.002); I² = 74%								
Test for overall effect: Z = 2.43 (P = 0.02)								
**2.5.4 Ketoprofen**								
KET20 (Matsumya L. C, et al. 2012) MGS	0.19	3.04	16	0.48	7.68	4	2.2%	-0.07 [-1.16, 1.03]
KET50 (Minville, V. et al. 2008) HT	11.91	1.24	10	5.95	2.88	10	1.8%	2.57 [1.33, 3.82]
KET50 (Minville, V. et al. 2008) VF	3.99	7.34	10	1.03	1.83	10	2.8%	0.53 [-0.37, 1.43]
**Subtotal (95% CI)**			**36**			**24**	**6.9%**	**0.97 [-0.45, 2.39]**
Heterogeneity: Tau² = 1.27; Chi² = 10.49, df = 2 (P = 0.005); I² = 81%								
Test for overall effect: Z = 1.34 (P = 0.18)								
**2.5.5 Meloxicam**								
MEL2(Cho, C. et al. 2019) MGS	0.11	0.88	8	0.42	3.36	8	2.5%	-0.12 [-1.10, 0.86]
MEL2 (Cho, C. et al. 2019) MGS	0.14	1.12	8	0.38	3.04	8	2.5%	-0.10 [-1.08, 0.88]
MEL2 (Cho, C. et al. 2019) MGS	0.08	0.64	8	0.38	3.04	8	2.5%	-0.13 [-1.11, 0.85]
MEL2 (Cho, C. et al. 2019) MGS	0.01	0.04	8	0.25	2	8	2.5%	-0.16 [-1.14, 0.82]
MEL5 (Cho, C. et al. 2019) MGS	0.2	1.6	8	0.42	3.36	8	2.5%	-0.08 [-1.06, 0.90]
MEL5 (Cho, C. et al. 2019) MGS	0.08	0.64	8	0.38	3.04	8	2.5%	-0.13 [-1.11, 0.85]
MEL5 (Cho, C. et al. 2019) MGS	0.06	0.48	8	0.28	2.24	8	2.5%	-0.13 [-1.11, 0.85]
MEL5 (Cho, C. et al. 2019) MGS	0.12	0.96	8	0.25	2	8	2.5%	-0.08 [-1.06, 0.90]
MEL5 (Roughan, V. J. et al. 2009) VS	50.54	20.7	8	10.97	27.26	8	2.0%	1.55 [0.39, 2.70]
MEL10 (Roughan, V. J. et al. 2009) VS	6.37	4.48	8	3.76	5.13	9	2.6%	0.51 [-0.46, 1.48]
MEL10 (Roughan, V. J. et al. 2016) VS	24.27	26.33	8	10.97	27.26	8	2.5%	0.47 [-0.53, 1.47]
MEL20 (Leach, M. C. et al. 2012) MGS	2.35	14.1	6	3.86	23.16	6	2.1%	-0.07 [-1.20, 1.06]
MEL20 (Roughan, V. J. et al. 2009) VS	16.95	14.11	8	10.97	27.26	8	2.5%	0.26 [-0.72, 1.25]
MEL20 (Wright-Williams, S. L. 2009) VS	8.5	9.8	8	17.1	30.8	8	2.5%	-0.36 [-1.35, 0.63]
**Subtotal (95% CI)**			**110**			**111**	**34.6%**	**0.08 [-0.19, 0.34]**
Heterogeneity: Tau² = 0.00; Chi² = 9.68, df = 13 (P = 0.72); I² = 0%								
Test for overall effect: Z = 0.55 (P = 0.58)								
**Total (95% CI)**			**355**			**320**	**100.0%**	**0.22 [0.03, 0.42]**
Heterogeneity: Tau² = 0.15; Chi² = 64.69, df = 43 (P = 0.02); I² = 34%								
Test for overall effect: Z = 2.23 (P = 0.03)								
Test for subgroup differences: Chi² = 6.73, df = 4 (P = 0.15), I² = 40.5%								

Std. Mean Difference
IV, Random, 95% CI

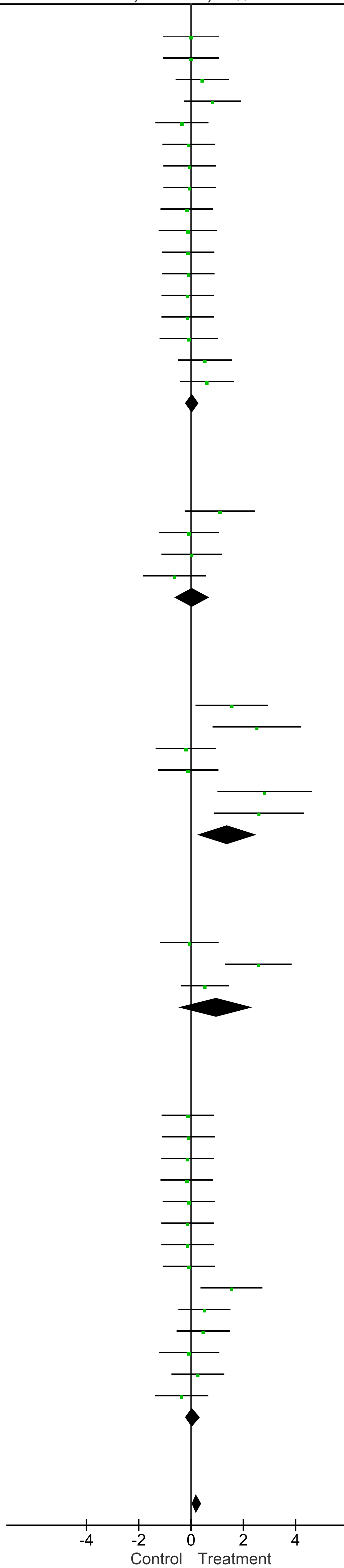

Supplementary figure 10. Meta-analysis based on the studies included in the meta-analysis of NSAID analgesic treatments. Forest plot presenting pooled SMDs (95% CI) for all NSAIDs included (carprofen, meloxicam, flunixin, diclofenac, ibuprofen, ketoprofen, celecoxib, dexketoprofen, firocoxib, indomethacin).

Supplementary Table 1 | All studies in the systematic review and meta-analysis.

Refer ence	Autor	Year	Pain Model	Strain	Sex	Treatment	Control	Dose	Route	Dos ing*	Elegible for MA?	Test	Evaluation time point	N of comparis ons
60	Adamson, T. W. et al.	2010	Mamary fat pad removal	FVB	F	Buprenorphine	Surgery+a naesthesia	0,2 mg/kg	SC	2	yes	RW	24h	3
60	Adamson, T. W. et al.	2010	Mamary fat pad removal	FVB	F	Carprofen	Surgery+a naesthesia	5 mg/kg	SC	1	yes	RW	24h	3
60	Adamson, T. W. et al.	2010	Mamary fat pad removal	FVB	F	Buprenorphine+C arprofen	Surgery+a naesthesia	5+0,2 mg/kg	SC	2	yes	RW	24h	3
60	Adamson, T. W. et al.	2010	Mamary fat pad removal	FVB	F	Buprenorphine	Surgery+a naesthesia	0,2 mg/kg	SC	2	yes	VS	24h	3
60	Adamson, T. W. et al.	2010	Mamary fat pad removal	FVB	F	Carprofen	Surgery+a naesthesia	5 mg/kg	SC	1	yes	VS	24h	3
60	Adamson, T. W. et al.	2010	Mamary fat pad removal	FVB	F	Buprenorphine+C arprofen	Surgery+a naesthesia	5+0,2 mg/kg	SC	2	yes	VS	24h	3
61	Brusco, I. et al.	2017	Plantar Incision	Swiss	M	Indometacin	Vehicle	1 mg/kg	PO	1	yes	VF	1h	3
61	Brusco, I. et al.	2017	Plantar Incision	Swiss	M	Indometacin	Vehicle	3 mg/kg	PO	1	yes	VF	1h	3
61	Brusco, I. et al.	2017	Plantar Incision	Swiss	M	Indometacin	Vehicle	10 mg/kg	PO	1	yes	SP	1h	2
61	Brusco, I. et al.	2017	Plantar Incision	Swiss	M	Indometacin post	Vehicle	10 mg/kg	PO	1	yes	SP	1h	2
61	Brusco, I. et al.	2017	Plantar Incision	Swiss	M	Indometacin	Vehicle	10 mg/kg	PO	1	yes	VF	1h	3
61	Brusco, I. et al.	2017	Plantar Incision	Swiss	M	Indometacin post	Vehicle	10 mg/kg	PO	1	yes	VF	1h	1
62	Cabañero & Maldonado	2021	Plantar Incision	CD1	M	Paracetamol	Vehicle	50 mg/kg	PO	1	yes	HT	2d	5
62	Cabañero & Maldonado	2021	Plantar Incision	CD1	M	Paracetamol	Vehicle	100 mg/kg	PO	1	yes	HT	2d	5
62	Cabañero & Maldonado	2021	Plantar Incision	CD1	M	Paracetamol	Vehicle	200 mg/kg	PO	1	yes	HT	2d	5
62	Cabañero & Maldonado	2021	Plantar Incision	CD1	M	Paracetamol	Vehicle	300 mg/kg	PO	1	yes	HT	2d	5
62	Cabañero & Maldonado	2021	Plantar Incision	CD1	M	Paracetamol	Vehicle	400 mg/kg	PO	1	yes	HT	2d	5

62	Cabañero & Maldonado	2021	Plantar Incision	CD1	M	Paracetamol	Vehicle	50 mg/kg	PO	1	yes	VF	2d	5
62	Cabañero & Maldonado	2021	Plantar Incision	CD1	M	Paracetamol	Vehicle	100 mg/kg	PO	1	yes	VF	2d	5
62	Cabañero & Maldonado	2021	Plantar Incision	CD1	M	Paracetamol	Vehicle	200 mg/kg	PO	1	yes	VF	2d	5
62	Cabañero & Maldonado	2021	Plantar Incision	CD1	M	Paracetamol	Vehicle	300 mg/kg	PO	1	yes	VF	2d	5
62	Cabañero & Maldonado	2021	Plantar Incision	CD1	M	Paracetamol	Vehicle	400 mg/kg	PO	1	yes	VF	2d	5
10	Cho, C. et al.	2019	Craniotomy	CD1	M	Carprofen	Vehicle	10 mg/kg	SC	>2	yes	MGS	6h	5
10	Cho, C. et al.	2019	Craniotomy	CD1	M	Carprofen	Vehicle	25 mg/kg	SC	>2	yes	MGS	6h	5
10	Cho, C. et al.	2019	Craniotomy	CD1	M	Meloxicam	Vehicle	2 mg/kg	SC	>2	yes	MGS	6h	5
10	Cho, C. et al.	2019	Craniotomy	CD1	M	Meloxicam	Vehicle	5 mg/kg	SC	>2	yes	MGS	6h	5
10	Cho, C. et al.	2019	Craniotomy	CD1	M	Buprenorphine	Vehicle	0,1 mg/kg	SC	>2	yes	MGS	6h	5
10	Cho, C. et al.	2019	Craniotomy	CD1	F	Carprofen	Vehicle	10 mg/kg	SC	>2	yes	MGS	6h	5
10	Cho, C. et al.	2019	Craniotomy	CD1	F	Carprofen	Vehicle	25 mg/kg	SC	>2	yes	MGS	6h	5
10	Cho, C. et al.	2019	Craniotomy	CD1	F	Meloxicam	Vehicle	2 mg/kg	SC	>2	yes	MGS	6h	5
10	Cho, C. et al.	2019	Craniotomy	CD1	F	Meloxicam	Vehicle	5 mg/kg	SC	>2	yes	MGS	6h	5
10	Cho, C. et al.	2019	Craniotomy	CD1	F	Buprenorphine	Vehicle	0,1 mg/kg	SC	>2	yes	MGS	6h	5
10	Cho, C. et al.	2019	Craniotomy	CD1	M	Carprofen	Vehicle	10 mg/kg	PO	1	yes	MGS	24h	5
10	Cho, C. et al.	2019	Craniotomy	CD1	M	Carprofen	Vehicle	25 mg/kg	PO	1	yes	MGS	24h	5
10	Cho, C. et al.	2019	Craniotomy	CD1	M	Meloxicam	Vehicle	2 mg/kg	PO	1	yes	MGS	24h	5
10	Cho, C. et al.	2019	Craniotomy	CD1	M	Meloxicam	Vehicle	5 mg/kg	PO	1	yes	MGS	24h	5
10	Cho, C. et al.	2019	Craniotomy	CD1	M	Buprenorphine	Vehicle	0,1 mg/kg	PO	1	yes	MGS	24h	5
10	Cho, C. et al.	2019	Craniotomy	CD1	F	Carprofen	Vehicle	10 mg/kg	PO	1	yes	MGS	24h	5
10	Cho, C. et al.	2019	Craniotomy	CD1	F	Carprofen	Vehicle	25 mg/kg	PO	1	yes	MGS	24h	5
10	Cho, C. et al.	2019	Craniotomy	CD1	F	Meloxicam	Vehicle	2 mg/kg	PO	1	yes	MGS	24h	5
10	Cho, C. et al.	2019	Craniotomy	CD1	F	Meloxicam	Vehicle	5 mg/kg	PO	1	yes	MGS	24h	5
10	Cho, C. et al.	2019	Craniotomy	CD1	F	Buprenorphine	Vehicle	0,1 mg/kg	PO	1	yes	MGS	24h	5
63	Clark, J. D. et al.	2007	Plantar Incision	C57bl/6	M	Morphine	Vehicle	0,1 mg/kg	SC	1	no	data		

63	Clark, J. D. et al.	2007	Plantar Incision	C57bl/6	M	Morphine	Vehicle	1 mg/kg	SC	2	yes	VF	2h	3
63	Clark, J. D. et al.	2007	Plantar Incision	C57bl/6	M	Morphine	Vehicle	10 mg/kg	SC	>2	yes	VF	2h	3
64	Clark, J. D. et al.	2004	Splenectomy	ICR	M	Oxymorphone LE	Surgery+anaesthesia	2 mg/kg	SC	>2	yes	VS	2h	1
64	Clark, J. D. et al.	2004	Splenectomy	ICR	M	Buprenorphine	Surgery+anaesthesia	0,2 mg/kg	SC	>2	yes	VS	2h	1
64	Clark, J. D. et al.	2004	Splenectomy	ICR	M	Buprenorphine	Surgery+anaesthesia	0,2 mg/kg	SC	>2	yes	RW	2h	1
65	Dallazen, J. L et al.	2022	Plantar Incision	Swiss	M	Morphine	Vehicle	1 mg/kg	SC	1	yes	VF	1h	1
65	Dallazen, J. L et al.	2022	Plantar Incision	Swiss	M	Morphine	Vehicle	1 mg/kg	SC	1	yes	HT	1h	1
65	Dallazen, J. L et al.	2022	Plantar Incision	Swiss	M	Morphine	Vehicle	1 mg/kg	SC	1	yes	SP	1h	1
66	Dickinson, A. L . et al.	2009	Vasectomy - Abdominal	CBA and DBA	M	Paracetamol	Vehicle	65.8 mg/kg	PO	1	no	Lacking control group		
66	Dickinson, A. L . et al.	2009	Vasectomy - Abdominal	CBA and DBA	M	Paracetamol	Vehicle	127.5 mg/kg	PO	1	no	Lacking control group		
66	Dickinson, A. L . et al.	2009	Vasectomy - Abdominal	CBA and DBA	M	Paracetamol	Vehicle	246.0 mg/kg	PO	1	no	Lacking control group		
67	Dorsch, M. M. et al.	2004	Laparotomy	C57bl/6J	F	Metamizol	Vehicle	200 mg/kg	SC	1	no	Insufficient Quantitative Data		
68	Durst, M. S. et al.	2021	Laparotomy	C57bl/6J	M	Paracetamol	Surgery+anaesthesia	200 mg/kg	PO	1	yes	NEST	2h	3
68	Durst, M. S. et al.	2021	Laparotomy	C57bl/6J	M	Lidocaine+bupivacaine	Surgery+anaesthesia	3+10 mg/kg	SC	1	yes	NEST	2h	3
68	Durst, M. S. et al.	2021	Laparotomy	C57bl/6J	M	Paracetamol+lidocaine+bupivacaine	Surgery+anaesthesia	200,3 and 10 mg/kg	SC	1	yes	NEST	2h	3
68	Durst, M. S. et al.	2021	Laparotomy	C57bl/6J	F	Paracetamol	Surgery+anaesthesia	200 mg/kg	PO	1	yes	NEST	2h	3
68	Durst, M. S. et al.	2021	Laparotomy	C57bl/6J	F	Lidocaine+bupivacaine	Surgery+anaesthesia	3+10 mg/kg	SC	1	yes	NEST	2h	3

68	Durst, M. S. et al.	2021	Laparotomy	C57bl/6J	F	Paracetamol+lidocaine+bupivacaine	Surgery+anaesthesia	200,3 and 10 mg/kg	SC	1	yes	NEST	2h	3
68	Durst, M. S. et al.	2021	Laparotomy	C57bl/6J	M	Paracetamol	Surgery+anaesthesia	200 mg/kg	PO	1	yes	BR	2h	3
68	Durst, M. S. et al.	2021	Laparotomy	C57bl/6J	M	Lidocaine+bupivacaine	Surgery+anaesthesia	3+10 mg/kg	SC	1	yes	BR	2h	3
68	Durst, M. S. et al.	2021	Laparotomy	C57bl/6J	M	Paracetamol+lidocaine+bupivacaine	Surgery+anaesthesia	200,3 and 10 mg/kg	SC	1	yes	BR	2h	3
68	Durst, M. S. et al.	2021	Laparotomy	C57bl/6J	F	Paracetamol	Surgery+anaesthesia	200 mg/kg	PO	1	yes	BR	2h	3
68	Durst, M. S. et al.	2021	Laparotomy	C57bl/6J	F	Lidocaine+bupivacaine	Surgery+anaesthesia	3,10 mg/kg	SC	1	yes	BR	2h	3
68	Durst, M. S. et al.	2021	Laparotomy	C57bl/6J	F	Paracetamol+lidocaine+bupivacaine	Surgery+anaesthesia	200,3 and 10 mg/kg	SC	1	yes	BR	2h	3
68	Durst, M. S. et al.	2021	Laparotomy	C57bl/6J	M	Paracetamol	Surgery+anaesthesia	200 mg/kg	PO	1	yes	MGS	1h	3
68	Durst, M. S. et al.	2021	Laparotomy	C57bl/6J	M	Lidocaine+bupivacaine	Surgery+anaesthesia	3+10 mg/kg	SC	1	yes	MGS	1h	3
68	Durst, M. S. et al.	2021	Laparotomy	C57bl/6J	M	Paracetamol+lidocaine+bupivacaine	Surgery+anaesthesia	200,3 and 10 mg/kg	SC	1	yes	MGS	1h	3
68	Durst, M. S. et al.	2021	Laparotomy	C57bl/6J	F	Paracetamol	Surgery+anaesthesia	200 mg/kg	PO	1	yes	MGS	1h	3
68	Durst, M. S. et al.	2021	Laparotomy	C57bl/6J	F	Lidocaine+bupivacaine	Surgery+anaesthesia	3+10 mg/kg	SC	1	yes	MGS	1h	3
68	Durst, M. S. et al.	2021	Laparotomy	C57bl/6J	F	Paracetamol+lidocaine+bupivacaine	Surgery+anaesthesia	200,3 and 10 mg/kg	SC	1	yes	MGS	1h	3
69	Evangelista-Vaz, R. et al.	2018	Laparotomy	C57bl/6J	F	Tramadol	Vehicle	25 mg/kg	SC/PO	1	yes	VS	1h	1
69	Evangelista-Vaz, R. et al.	2018	Laparotomy	C57bl/6J	F	Tramadol	Vehicle	25 mg/kg	SC/PO	1	yes	BR	2h	1

44	Faller, K. M. E. et al.	2015	Thoracotomy	C57bl/6J	F	Buprenorphine	Vehicle	1,1 mg/kg	SC	1	yes	MGS	24h	1
21	Furumoto, K. et al.	2021	Laparotomy	ICR	M	Buprenorphine + meloxicam + bupivacaine	Vehicle	0,1 mg/kg	SC	>2	yes	NEST	2h	2
45	Gallo, M. S. et al.	2020	Carotid artery catheterization	CD1	M	Buprenorphine	Vehicle	0,05 mg/kg	SC	2	no	Insufficient Quantitative Data		
70	Goecke, J. C. et al.	2005	Laparotomy	ICR	F	Flunixin Meglumine	Vehicle	2,5 mg/kg	SC	1	yes	VS	24h	4
70	Goecke, J. C. et al.	2005	Laparotomy	ICR	F	Flunixin Meglumine	Vehicle	2,5 mg/kg	SC	>2	yes	VS	24h	4
70	Goecke, J. C. et al.	2005	Laparotomy	ICR	F	Buprenorphine	Vehicle	2 mg/kg	SC	1	yes	VS	24h	4
70	Goecke, J. C. et al.	2005	Laparotomy	ICR	F	Buprenorphine	Vehicle	2 mg/kg	SC	>2	yes	VS	24h	4
11	Jirkof, P et al.	2010	Laparotomy	C57bl/6J	M/F	Carprofen	Surgery+anaesthesia	5 mg/kg	SC	1	yes	BR	2h	1
71	Jirkof, P et al.	2012	Laparotomy	C57bl/6J	F	Carprofen	Surgery+anaesthesia	5 mg/kg	SC	1	yes	VS	24h	1
72	Jirkof, P et al.	2013	Laparotomy	C57BL/6J and DBA	M/F	Carprofen	Surgery+anaesthesia	5 mg/kg	SC	1	yes	NEST	9h	2
72	Jirkof, P et al.	2013	Laparotomy	C57BL/6J and DBA	M/F	Carprofen	Surgery+anaesthesia	50 mg/kg	SC	1	yes	NEST	9h	2
73	Jirkof, P et al.	2015	Laparotomy	C57bl/6J	F	Buprenorphine	Vehicle	0,1 mg/kg	SC	>2	yes	VS	12h	2
73	Jirkof, P et al.	2015	Laparotomy	C57bl/6J	F	Buprenorphine SR	Vehicle	2,2 mg/kg	SC	1	yes	VS	12h	2
73	Jirkof, P et al.	2015	Laparotomy	C57bl/6J	F	Buprenorphine	Vehicle	0,1 mg/kg	SC	>2	yes	BR	12h	1
73	Jirkof, P et al.	2015	Laparotomy	C57bl/6J	F	Buprenorphine SR	Vehicle	2,2 mg/kg	SC	1	yes	BR	12h	1
74	Jirkof, P et al.	2018	Laparotomy	C57bl/6J	F	Tramadol+Paracetamol	Surgery+anaesthesia	1, 5 mg/kg	PO	1	yes	NEST	4h	1
74	Jirkof, P et al.	2018	Laparotomy	C57bl/6J	F	Tramadol+Paracetamol	Surgery+anaesthesia	1, 5 mg/kg	PO	1	yes	VS	1h	1
74	Jirkof, P et al.	2018	Laparotomy	C57bl/6J	F	Tramadol+Paracetamol	Surgery+anaesthesia	1, 5 mg/kg	PO	1	yes	VS	3h	1
74	Jirkof, P et al.	2018	Laparotomy	C57bl/6J	F	Tramadol+Paracetamol	Surgery+anaesthesia	1, 5 mg/kg	PO	1	yes	MGS	1h	1
75	Jirkof, P et al.	2019	Osteotomy	C57bl/6N	F	Tramadol	Surgery+anaesthesia	0,1 mg/ml	SC	1	yes	MGS	1h	1
75	Jirkof, P et al.	2019	Osteotomy	C57bl/6N	F	Tramadol	Surgery+anaesthesia	1 mg/ml	SC	1	yes	MGS	1h	1

75	Jirkof, P et al.	2019	Osteotomy	C57bl/6N	F	Buprenorphine	Surgery+a naesthesia	0,009 mg/ml	PO	1	yes	MGS	1h	1
75	Jirkof, P et al.	2019	Osteotomy	C57bl/6N	F	Tramadol	Surgery+a naesthesia	0,1 mg/ml	SC	1	yes	MGS	6h	1
75	Jirkof, P et al.	2019	Osteotomy	C57bl/6N	F	Tramadol	Surgery+a naesthesia	1 mg/ml	SC	1	yes	MGS	6h	1
75	Jirkof, P et al.	2019	Osteotomy	C57bl/6N	F	Buprenorphine	Surgery+a naesthesia	0,009 mg/ml	PO	1	yes	MGS	6h	1
76	Kendal, L. V. et al.	2016	Laparotom y	ICR	F	Buprenorphine SR	Vehicle	0,6 kg/kg	SC	1	yes	RW	48h	1
76	Kendal, L. V. et al.	2016	Laparotom y	ICR	F	Buprenorphine HCl	Vehicle	0,1 mg/kg	SC	>2	yes	RW	48h	1
76	Kendal, L. V. et al.	2016	Laparotom y	ICR	F	Buprenorphine SR	Vehicle	0,6 kg/kg	SC	1	yes	NEST	6h	2
76	Kendal, L. V. et al.	2016	Laparotom y	ICR	F	Buprenorphine HCl	Vehicle	0,1 mg/kg	SC	>2	yes	NEST	6h	2
76	Kendal, L. V. et al.	2016	Laparotom y	ICR	F	Buprenorphine SR	Vehicle	0,6 kg/kg	SC	1	yes	VS	6h	1
76	Kendal, L. V. et al.	2016	Laparotom y	ICR	F	Buprenorphine HCl	Vehicle	0,1 mg/kg	SC	>2	yes	VS	6h	1
32	Leach, M. C. et al.	2012	Vasectomy - Scrotal	CD1	M	Bupivacaine	Vehicle	5 mg/kg	SC	1	yes	MGS	1h	2
32	Leach, M. C. et al.	2012	Vasectomy - Scrotal	CD1	M	Meloxicam	Vehicle	20 mg/kg	SC	1	yes	MGS	1h	2
77	Lu, F. et al.	2022	Plantar Incision	C57bl/6J	M	Diclofenac	Vehicle	0,25 mg/250 µL	SC	>2	yes	VF	6h	2
77	Lu, F. et al.	2022	Plantar Incision	C57bl/6J	M	Buprenorphine	Vehicle	1.67×10^{-3} mg/250 µL	SC	>2	yes	VF	6h	2
77	Lu, F. et al.	2022	Plantar Incision	C57bl/6J	M	Diclofenac	Vehicle	0,25 mg/250 µL	SC	>2	yes	VF	3d	1
22	Matsumya L. C, et al.	2012	Laparotom y	CD1	M/F	Carprofen	Vehicle	20 mg/kg	SC	>2	yes	MGS	1h	4
22	Matsumya L. C, et al.	2012	Laparotom y	CD1	M/F	Carprofen	Vehicle	25 mg/kg	SC	>2	yes	MGS	1h	4
22	Matsumya L. C, et al.	2012	Laparotom y	CD1	M/F	Ketoprofen	Vehicle	20 mg/kg	SC	>2	yes	MGS	1h	4
22	Matsumya L. C, et al.	2012	Laparotom y	CD1	M/F	Buprenorphine	Vehicle	0,05 mg/kg	SC	>2	yes	MGS	1h	4
22	Matsumya L. C, et al.	2012	Laparotom y	CD1	M/F	Buprenorphine	Vehicle	0,1 mg/kg	SC	>2	yes	MGS	1h	4
78	Minville, V. et al.	2008	Osteotomy	C57bl/6J	M	Ketoprofen	Vehicle	50 mg/kg	SC	1	yes	HT	1h	3
78	Minville, V. et al.	2008	Osteotomy	C57bl/6J	M	Morphine	Vehicle	3 mg/kg	SC	1	yes	HT	1h	3

78	Minville, V. et al.	2008	Osteotomy	C57bl/6J	M	Morphine	Vehicle	10 mg/kg	SC	1	yes	HT	1h	3
78	Minville, V. et al.	2008	Osteotomy	C57bl/6J	M	Ketoprofen	Vehicle	50 mg/kg	SC	1	yes	VF	1,5h	3
78	Minville, V. et al.	2008	Osteotomy	C57bl/6J	M	Morphine	Vehicle	3 mg/kg	SC	1	yes	VF	30 min	3
78	Minville, V. et al.	2008	Osteotomy	C57bl/6J	M	Morphine	Vehicle	10 mg/kg	SC	1	yes	VF	30 min	3
79	Molina-Cimadevila, M. J. et al.	2014	Laparotomy	CD1	F	Meloxicam+buprenorphine	Vehicle	Bup 0,1 mg/kg + Mel 0,25 mg/pellet	SC	2	yes	HT	4h	3
79	Molina-Cimadevila, M. J. et al.	2014	Laparotomy	CD1	F	Buprenorphine	Vehicle	0Bup 0,1 mg/kg + 0,3 mg/pellet	SC	2	yes	HT	4h	3
79	Molina-Cimadevila, M. J. et al.	2014	Laparotomy	CD1	F	Buprenorphine	Vehicle	0Bup 0,1 mg/kg + 0,15 mg/pellet	SC	2	yes	HT	4h	3
80	Miller, A. L et al.	2012	Vasectomy - Laparotomy	CD1	M	Meloxicam	Vehicle	5 mg/kg	SC	1	no	Lacking control group		
80	Miller, A. L et al.	2012	Vasectomy - Laparotomy	CD1	M	Meloxicam+Paracetamol	Vehicle	5+50 mg/kg	SC/IP	1	no	Lacking control group		
81	Mueller, A. et al.	2019	Plantar Incision	C57bl/6	M	Oxycodone	Vehicle	3 mg/kg	IP	1	yes	VF	24h	1
82	Muratani T. et al.	2005	Plantar Incision	DDY	M	Celecoxibe	Vehicle	1 mg/kg	PO	1	yes	VF	3d pré	2
82	Muratani T. et al.	2005	Plantar Incision	DDY	M	Celecoxibe	Vehicle	1 mg/kg	PO	1	yes	VF	3d pré	2
82	Muratani T. et al.	2005	Plantar Incision	DDY	M	Celecoxibe	Vehicle	10 mg/kg	PO	1	yes	VF	3d post	2
82	Muratani T. et al.	2005	Plantar Incision	DDY	M	Celecoxibe	Vehicle	10 mg/kg	PO	1	yes	VF	3d post	2
83	Navarro K, et al.	2020	Plantar Incision	C57bl/6	M	Buprenorphine SR	Vehicle	SR 1 mg/kg	SC	1	yes	HT	4h	3
83	Navarro K, et al.	2020	Plantar Incision	C57bl/6	M	Buprenorphine XR	Vehicle	XR 3,25 mg/kg	SC	1	yes	HT	4h	3
83	Navarro K, et al.	2020	Plantar Incision	C57bl/6	M	Buprenorphine XR	Vehicle	XR 6,5 mg/kg	SC	1	yes	HT	4h	3
83	Navarro K, et al.	2020	Plantar Incision	C57bl/6	M	Buprenorphine SR	Vehicle	SR 1 mg/kg	SC	1	yes	VF	4h	3
83	Navarro K, et al.	2020	Plantar Incision	C57bl/6	M	Buprenorphine XR	Vehicle	XR 3,25 mg/kg	SC	1	yes	VF	4h	3

83	Navarro K, et al.	2020	Plantar Incision	C57bl/6	M	Buprenorphine XR	Vehicle	XR 6,5 mg/kg	SC	1	yes	VF	4h	3
84	Oliver, V. L. et al.	2018	Laparotomy	C57bl/6	M/F	Buprenorphine	Vehicle	0,1 mg/kg	SC	>2	yes	VF	4h	2
84	Oliver, V. L. et al.	2018	Laparotomy	C57bl/6	M/F	Carprofen	Vehicle	30 mg/kg	PO	1	yes	VF	4h	2
85	Park, J. H et al.	2020	Plantar Incision	BalbC	M	Pregabalin	Vehicle	30 mg/kg	IP	1	yes	VF	2h	1
86	Pogatzki, E. M. & Raja, S. N.	2003	Plantar Incision	C3H	M	Morphine	Vehicle	3 mg/kg	SC	1	yes	HT	30 min	2
86	Pogatzki, E. M. & Raja, S. N.	2003	Plantar Incision	C3H	M	Morphine	Vehicle	10 mg/kg	SC	1	yes	HT	30 min	2
86	Pogatzki, E. M. & Raja, S. N.	2003	Plantar Incision	C3H	M	Morphine	Vehicle	10 mg/kg	SC	1	yes	VF	30 min	1
87	Reddyjarugu, B. et al.	2015	Plantar Incision	C57BL/6N CrL	F	Buprenorphine	Vehicle	0,2 mg/kg	SC	>2	yes	HT	4h	3
87	Reddyjarugu, B. et al.	2015	Plantar Incision	C57BL/6N CrL	F	Firocoxibe	Vehicle	10 mg/kg	IP	>2	yes	HT	4h	3
87	Reddyjarugu, B. et al.	2015	Plantar Incision	C57BL/6N CrL	F	Firocoxibe	Vehicle	20 mg/kg	IP	>2	yes	HT	4h	3
87	Reddyjarugu, B. et al.	2015	Plantar Incision	C57BL/6N CrL	F	Buprenorphine	Vehicle	0,2 mg/kg	SC	>2	yes	VF	3d	3
87	Reddyjarugu, B. et al.	2015	Plantar Incision	C57BL/6N CrL	F	Firocoxibe	Vehicle	10 mg/kg	IP	>2	yes	VF	2 d	3
87	Reddyjarugu, B. et al.	2015	Plantar Incision	C57BL/6N CrL	F	Firocoxibe	Vehicle	20 mg/kg	IP	>2	yes	VF	3d	3
88	Romero-Alejo, E., et al.	2016	Plantar Incision	CD1	M	Tramadol+dexketoprofen	Vehicle	TRA82, DEX60	SC	1	yes	VF	4h	3
88	Romero-Alejo, E., et al.	2016	Plantar Incision	CD1	M	Dexketoprofen	Vehicle	100 mg/kg	SC	1	yes	VF	4h	3
88	Romero-Alejo, E., et al.	2016	Plantar Incision	CD1	M	Tramadol	Vehicle	100 mg/kg	SC	1	yes	VF	4h	3
89	Roughan, V. J. et al.	2009	Vasectomy	C3H and C57BL/6	M	Meloxicam	Vehicle	5 mg/kg	SC	1	yes	VS	1h	3
89	Roughan, V. J. et al.	2009	Vasectomy	C3H and C57BL/7	M	Meloxicam	Vehicle	10 mg/kg	SC	1	yes	VS	1h	3
89	Roughan, V. J. et al.	2009	Vasectomy	C3H and C57BL/8	M	Meloxicam	Vehicle	20 mg/kg	SC	1	yes	VS	1h	3
90	Roughan, V. J. et al.	2016	Laparotomy	BalbC	M	Meloxicam	Vehicle	1 mg/kg	SC	1	yes	VS	1h	3
90	Roughan, V. J. et al.	2016	Laparotomy	BalbC	M	Meloxicam	Vehicle	5 mg/kg	SC	1	yes	VS	1h	3
90	Roughan, V. J. et al.	2016	Laparotomy	BalbC	M	Meloxicam	Vehicle	10 mg/kg	SC	1	yes	VS	1h	3

91	Saad, S. S. T. et al.	2016	Plantar incision	Swiss	M	Ibuprofen	Vehicle	30 mg/kg	IP	1	no	data		
91	Saad, S. S. T. et al.	2016	Plantar incision	Swiss	M	Ibuprofen	Vehicle	100 mg/kg	IP	1	yes	VF	4h	3
91	Saad, S. S. T. et al.	2016	Plantar incision	Swiss	M	Ibuprofen	Vehicle	300 mg/kg	IP	1	yes	VF	4h	3
92	Tonello, R. et al.	2020	Plantar Incision	Swiss	F	Morphine	Vehicle	1 mg/kg	SC	1	no	data		
92	Tonello, R. et al.	2020	Plantar Incision	Swiss	F	Morphine	Vehicle	3 mg/kg	SC	1	yes	SP	1h	3
92	Tonello, R. et al.	2020	Plantar Incision	Swiss	F	Morphine	Vehicle	10 mg/kg	SC	1	yes	SP	1h	3
92	Tonello, R. et al.	2020	Plantar Incision	Swiss	F	Morphine	Vehicle	3 mg/kg	SC	1	yes	VF	1h	3
92	Tonello, R. et al.	2020	Plantar Incision	Swiss	F	Morphine	Vehicle	10 mg/kg	SC	1	yes	VF	1h	3
93	Wei, S. et al.	2021	Plantar Incision	Kunming	M	Morphine	Vehicle	1 nmol	IT	1	no	data		
93	Wei, S. et al.	2021	Plantar Incision	Kunming	M	Morphine	Vehicle	5 nmol	IT	>2	yes	VF	10 min	4
93	Wei, S. et al.	2021	Plantar Incision	Kunming	M	Morphine	Vehicle	3 nmol	IT	1	yes	VF	10 min	4
93	Wei, S. et al.	2021	Plantar Incision	Kunming	M	Morphine	Vehicle	10 nmol	IT	1	yes	VF	10 min	4
94	Wolfe, A. M. et al.	2015	Laparotomy	C57/bl6	F	Tramadol	Vehicle	20 mg/kg	SC	>2	yes	VS	24h	3
94	Wolfe, A. M. et al.	2015	Laparotomy	C57/bl6	F	Tramadol	Vehicle	40 mg/kg	SC	>2	yes	VS	24h	3
94	Wolfe, A. M. et al.	2015	Laparotomy	C57/bl6	F	Tramadol	Vehicle	80 mg/kg	SC	>2	yes	VS	24h	3
94	Wolfe, A. M. et al.	2015	Laparotomy	C57/bl6	F	Tramadol	Vehicle	80 mg/kg	SC	>2	yes	VS	24h	3
95	Wright-Williams, S. L.	2007	Vasectomy - Abdominal	C57bl/6J and C3H	M	Meloxicam	Vehicle	5 mg/kg	SC	1	yes	VS	1h	3
95	Wright-Williams, S. L.	2007	Vasectomy - Abdominal	C57bl/6J and C3H	M	Meloxicam	Vehicle	10 mg/kg	SC	1	yes	VS	1h	3
95	Wright-Williams, S. L.	2007	Vasectomy - Abdominal	C57bl/6J and C3H	M	Meloxicam	Vehicle	20 mg/kg	SC	1	yes	VS	1h	3
96	Wright-Williams, S. L.	2013	Vasectomy - Abdominal	C57bl/6J and C3H	M	Buprenorphine	Vehicle	0,01 mg/kg	SC	1	yes	VS	1h	2

96	Wright-Williams, S. L.	2013	Vasectomy - Abdominal	C57bl/6J and C3H	M	Buprenorphine	Vehicle	0,05 mg/kg	SC	1	yes	VS	1h	2
97	Yang J. et al.	2019	SMIR	C57bl/6	M	Gabapentin	Vehicle	10 mg/kg	IP	1	no	data		
97	Yang J. et al.	2019	SMIR	C57bl/6	M	Gabapentin	Vehicle	20 mg/kg	IP	1	yes	VF	30 min	3
97	Yang J. et al.	2019	SMIR	C57bl/6	M	Gabapentin	Vehicle	50 mg/kg	IP	1	yes	VF	30 min	3
98	Yamada, T. et al.	2016	Plantar Incision	C57bl/6	M	Ropivacaine	Vehicle	0,05	NB	1	yes	HT	30 min	1
98	Yamada, T. et al.	2016	Plantar Incision	C57bl/6	M	Ropivacaine	Vehicle	0,05	NB	1	yes	HT	1h	1
98	Yamada, T. et al.	2016	Plantar Incision	C57bl/6	M	Ropivacaine	Vehicle	0,05	NB	1	yes	VF	30 min	1
98	Yamada, T. et al.	2016	Plantar Incision	C57bl/6	M	Ropivacaine	Vehicle	0,05	NB	1	yes	VF	1h	1
99	Zhao W. et al.	2018	Knee artroplasty	Kunming	NR	Bupivacaine	Vehicle	45 mM	NB	1	no	Insufficient Quantitative Data		
99	Zhao W. et al.	2018	Knee artroplasty	Kunming	NR	levobupivacaine	Vehicle	10 mM	NB	1	no	Insufficient Quantitative Data		
100	Zhu, Q. et al.	2016	Plantar Incision	ICR	M	Paracetamol	Vehicle	30 mg/kg	IP	>2	yes	VF	2h	5
100	Zhu, Q. et al.	2016	Plantar Incision	ICR	M	Paracetamol	Vehicle	60 mg/kg	IP	>2	yes	VF	2h	5
100	Zhu, Q. et al.	2016	Plantar Incision	ICR	M	Paracetamol	Vehicle	120 mg/kg	IP	>2	yes	VF	2h	5
100	Zhu, Q. et al.	2016	Plantar Incision	ICR	M	Paracetamol	Vehicle	240 mg/kg	IP	>2	yes	VF	2h	5
100	Zhu, Q. et al.	2016	Plantar Incision	ICR	M	Paracetamol	Vehicle	360 mg/kg	IP	>2	yes	VF	2h	5
