# Supplementary material for: Analgesic drug efficacy in mouse postoperative pain: a systematic review and meta-analysis
Source: Lab Anim (NY). 2026 Jun 23;55(9):334–43. doi: 10.1038/s41684-026-01750-5 (PMC13518225; doi:10.1038/s41684-026-01750-5)
Supplement: Supplementary file 2 [file 41684_2026_1750_MOESM3_ESM.pdf]

Multilevel Meta-analysis R Script

Author: Roberta Schran

1. Load required packages

```
install.packages(c(
  "readxl", "dplyr", "stringr", "metafor",
  "tidyr", "ggplot2", "forestplot"
))

library(readxl)
library(dplyr)
library(stringr)
library(metafor)
library(tidyr)
library(ggplot2)
library(forestplot)
```

2. Import dataset

```
file <- "Mouse_POP_RSMA_DATASET.xlsx"
sheet <- "All_comparisons"

data_raw <- read_excel(path = file, sheet = sheet)

head(data_raw)
glimpse(data_raw)
```

3. Clean and standardize numeric variables

```
num_cols <- c("Treated_mean", "Treated_SD", "Treated_N",
              "Control_mean", "Control_SD", "Control_N")

data <- data_raw %>%
  mutate(across(all_of(num_cols), as.character)) %>%
  mutate(across(all_of(num_cols), ~ str_replace_all(.x, ",", "."))) %>%
  mutate(across(all_of(num_cols), ~ str_replace_all(.x, "[^0-9eE.\\-\\+]", "")))
%>%
  mutate(across(all_of(num_cols), ~ na_if(.x, ""))) %>%
  mutate(across(all_of(num_cols), as.numeric))

summary(data[num_cols])
```

4. Replace invalid values (e.g., SD = 0)

```
data <- data %>%
  mutate(
    Treated_SD = ifelse(Treated_SD == 0, 0.001, Treated_SD),
    Control_SD = ifelse(Control_SD == 0, 0.001, Control_SD)
  )
```

5. Compute effect sizes (Hedges' g)

```
data_es <- escalc(
  measure = "SMDH",
  m1i = Treated_mean, sd1i = Treated_SD, n1i = Treated_N,
  m2i = Control_mean, sd2i = Control_SD, n2i = Control_N,
  data = data,
  var.names = c("yi", "vi")
)
dados_final <- data_es %>%
```

```
  mutate(Comparison_ID = row_number())
head(data_es %>% select(Study_ID, Treatment, yi, vi))
```

6. Multilevel random-effects model

```
data_es <- data_es %>%
  mutate(Comparison_ID = row_number())
model_ml <- rma.mv(
  yi, vi,
  random = ~ 1 | Study_ID / Comparison_ID,
  data = data_es,
  method = "REML"
)
summary(model_ml)
```

7. Egger’s regression test

```
data_es <- data_es %>%
  mutate(se = sqrt(vi))

egger <- rma(
  yi = yi, sei = se,
  mods = ~ se,
  data = data_es,
  method = "REML"
)
summary(egger)
```

8. Funnel plot

```
ggplot(data_es, aes(x = yi, y = 1/se)) +
```

```
  geom_point(alpha = 0.6) +
  geom_vline(xintercept = model_ml$b[1],
             color = "red", linetype = "dashed") +
  theme_minimal() +
  labs(
    title = "Funnel Plot (Multilevel Meta-analysis)",
    x = "Effect size (Hedges' g)",
    y = "Precision (1/SE)"
  )
```

9. Function for subgroup analyses

```
overall_DL  <- rma(yi, vi, data = dados_final, method = "DL")
dados_final$Comparison_ID <- seq_len(nrow(dados_final))
overall_MV <- rma.mv(
  yi, vi,
  random = ~ 1 | Study_ID/Comparison_ID,
  data = dados_final,
  method = "REML"
)
meta_subgroup_DL <- function(data, group_var){
  data %>%
    group_by(.data[[group_var]]) %>%
    summarise(
      k = n(),
      model = list(rma(yi, vi, data = pick(everything()), method = "DL")),
      SMD   = model[[1]]$b,
      CI_lb = model[[1]]$ci.lb,
      CI_ub = model[[1]]$ci.ub,
```

```
      I2    = model[[1]]$I2
    ) %>% ungroup()
  }
  meta_subgroup_mv <- function(data, group_var){
   data %>%
    group_by(.data[[group_var]]) %>%
    summarise(
     k = n(),
     model = list(rma.mv(
      yi, vi,
      random = ~ 1 | Study_ID/Comparison_ID,
      data = pick(everything()),
      method = "REML"
     )),
     SMD   = model[[1]]$b,
     CI_lb = model[[1]]$ci.lb,
     CI_ub = model[[1]]$ci.ub
    ) %>% ungroup()
  }
# Sex
  sex_DL <- meta_subgroup_DL(dados_final, "Sex")
  sex_MV <- meta_subgroup_mv(dados_final, "Sex")
# Severity
  severity_DL <- meta_subgroup_DL(dados_final, "Severity")
  severity_MV <- meta_subgroup_mv(dados_final, "Severity")
# Analgesic classes
  class_DL <- meta_subgroup_DL(dados_final, "Treatment_class")
  class_MV <- meta_subgroup_mv(dados_final, "Treatment_class")
```

```
# PAIN MODALITY (Evoked / Spont / Suppressed)
	modality_DL <- meta_subgroup_DL(dados_final, "Test_type")
	modality_MV <- meta_subgroup_mv(dados_final, "Test_type")


	cat("\n==== Overall (DL) ====\n")
	print(overall_DL)
	cat("\n==== Overall (Multilevel) ====\n")
	print(summary(overall_MV))
	cat("\n==== SEX – DL ====\n"); print(sex_DL)
	cat("\n==== SEX – Multilevel ====\n"); print(sex_MV)
	cat("\n==== SEVERITY – DL ====\n"); print(severity_DL)
	cat("\n==== SEVERITY – Multilevel ====\n"); print(severity_MV)
	cat("\n==== ANALGESIC CLASS – DL ====\n"); print(class_DL)
	cat("\n==== ANALGESIC CLASS – Multilevel ====\n"); print(class_MV)
	cat("\n==== PAIN MODALITY – DL ====\n"); print(modality_DL)
	cat("\n==== PAIN MODALITY – Multilevel ====\n"); print(modality_MV)
```
